# *CELSR2* is a candidate susceptibility gene in idiopathic scoliosis

**DOI:** 10.1371/journal.pone.0189591

**Published:** 2017-12-14

**Authors:** Elisabet Einarsdottir, Anna Grauers, Jingwen Wang, Hong Jiao, Stefan A. Escher, Aina Danielsson, Ane Simony, Mikkel Andersen, Steen Bach Christensen, Kristina Åkesson, Ikuyo Kou, Anas M. Khanshour, Acke Ohlin, Carol Wise, Shiro Ikegawa, Juha Kere, Paul Gerdhem

**Affiliations:** 1 Folkhälsan Institute of Genetics, and Molecular Neurology Research Program, University of Helsinki, Helsinki, Finland; 2 Department of Biosciences and Nutrition, Karolinska Institutet, Huddinge, Sweden; 3 Department of Orthopaedics, Sundsvall and Härnösand County Hospital, Sundsvall, Sweden; 4 Department of Clinical Science, Intervention and Technology (CLINTEC), Karolinska Institutet, Stockholm, Sweden; 5 Genetic and Molecular Epidemiology Unit, Lund University Diabetes Centre, Department of Clinical Sciences, Lund University, Malmö, Sweden; 6 Department of Orthopaedics, Institute of Clinical Sciences, Sahlgren Academy at Gothenburg University, Göteborg, Sweden; 7 Department of Orthopaedics, Sahlgren University Hospital, Göteborg, Sweden; 8 Sector for Spine Surgery & Research, Middelfart Hospital, Middelfart, Denmark; 9 Lund University, Department of Clinical Sciences Malmö, Clinical and Molecular Osteoporosis Research Unit, Malmö, Sweden; 10 Skåne University Hospital, Department of Orthopedics, Malmö, Sweden; 11 Laboratory of Bone and Joint Diseases, Center for Integrative Medical Sciences, RIKEN, Tokyo, Japan; 12 Sarah M. and Charles E. Seay Center for Musculoskeletal Research, Texas Scottish Rite Hospital for Children, Dallas, Texas, United States of America; 13 Department of Orthopaedics, Skåne University Hospital, Malmö, Sweden; 14 McDermott Center for Human Growth and Development and Departments of Pediatrics and Orthopaedic Surgery, University of Texas Southwestern Medical Center at Dallas, Dallas, Texas, United States of America; 15 Department of Medical & Molecular Genetics, King’s College London, Guy’s Hospital, London, United Kingdom; 16 Department of Orthopaedics, Karolinska University Hospital, Huddinge, Sweden; Huashan Hospital Fudan University, CHINA

## Abstract

A Swedish pedigree with an autosomal dominant inheritance of idiopathic scoliosis was initially studied by genetic linkage analysis, prioritising genomic regions for further analysis. This revealed a locus on chromosome 1 with a putative risk haplotype shared by all affected individuals. Two affected individuals were subsequently exome-sequenced, identifying a rare, non-synonymous variant in the *CELSR2* gene. This variant is rs141489111, a c.G6859A change in exon 21 (NM_001408), leading to a predicted p.V2287I (NP_001399.1) change. This variant was found in all affected members of the pedigree, but showed reduced penetrance. Analysis of tagging variants in *CELSR1-3* in a set of 1739 Swedish-Danish scoliosis cases and 1812 controls revealed significant association (p = 0.0001) to rs2281894, a common synonymous variant in *CELSR2*. This association was not replicated in case-control cohorts from Japan and the US. No association was found to variants in *CELSR1* or *CELSR3*. Our findings suggest a rare variant in *CELSR2* as causative for idiopathic scoliosis in a family with dominant segregation and further highlight common variation in *CELSR2* in general susceptibility to idiopathic scoliosis in the Swedish-Danish population. Both variants are located in the highly conserved GAIN protein domain, which is necessary for the auto-proteolysis of CELSR2, suggesting its functional importance.

## Introduction

Idiopathic scoliosis is the most common spinal deformity manifesting in children and adolescents, and is characterised by an abnormal structural curvature of the spine. The prevalence of idiopathic scoliosis is approximately 2–3% worldwide [1, 2]. The cause of this disorder remains elusive, but studies on twins have shown a heritability of approx. 40%, indicating the importance of genetic factors [3].

Genome wide association studies have identified several common genetic variants that modulate the susceptibility to this disorder [4–7] and linkage studies and exome sequencing in families with a high burden of idiopathic scoliosis have suggested some rare variants that modulate susceptibility to this disorder [8–13]. The function of the associated genes/variants is in most cases unclear and their role in the pathogenesis of idiopathic scoliosis unknown. It is furthermore clear that much of the underlying genetic risk factors and mechanisms still remain to be identified.

The aim of the current study was to identify the hypothesized single gene underlying an apparently dominant form of idiopathic scoliosis in a family from Sweden. An initial linkage analysis was combined with subsequent exome sequencing to enable prioritization of genome regions for risk variant search. Our analysis highlighted a rare non-synonymous variant in *CELSR2* as a plausible idiopathic scoliosis risk variant. The identification of *CELSR2* and understanding how it might contribute to the risk of idiopathic scoliosis may shed further light on the pathways and mechanisms involved in the pathogenesis of idiopathic scoliosis.

## Material & methods

All studies were carried out in accordance with the relevant guidelines and regulations and all participating individuals gave their written informed consent. The Ethical Committee at Lund University (LU 200–95, LU 280–99, LU 363–02), the Regional Ethical Review Board in Stockholm (290/2006, 2009/1124-31/2, 2012/1595-31/2) and Lund (567/2008, 2014/804), and the Regional Committees on Health Research Ethics for Southern Denmark (S-2011002) approved the study.

### Samples

#### Swedish family

**Fig 1:**
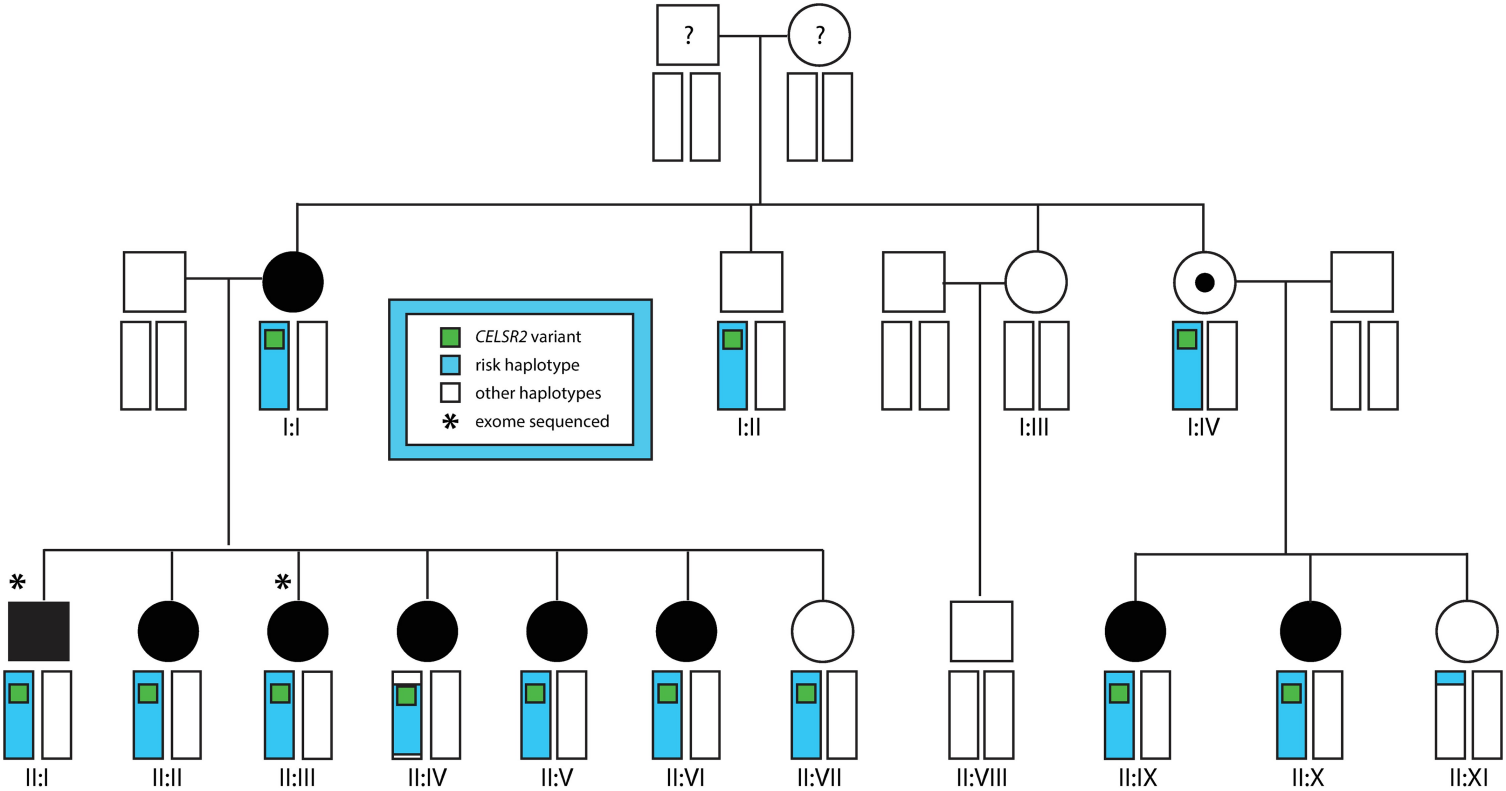
The pedigree included in the current study. Affected individuals are indicated in black, unaffected in white. An obligate carrier of the chromosome 1 putative scoliosis risk haplotype (denoted by blue bars) is marked with a black dot. All numbered individuals have been genotyped and included in the linkage analysis. All putative non-risk haplotypes are denoted by white bars. The two exome-sequenced individuals are marked with asterisks. Carriers of the rare *CELSR2* variant identified by exome sequencing are marked by a green box.

A three-generation family (Fig 1) with a high burden of idiopathic scoliosis participated in the study. All participating family members (n = 15) were blood-sampled and radiographed. All but one (I:II) were examined by a spine surgeon; however this individual had no scoliosis on radiographs. Individuals with a curve angle of 10 degrees or more, as measured by the Cobb method [14], were diagnosed with scoliosis. No one had any signs of a non-idiopathic scoliosis and all had an onset in adolescence, i.e. after the age of ten years (S1 Table). DNA was extracted from blood either by a salt precipitation method on the Autopure LS system (Qiagen, Hilden, Germany) or the QIAamp 96 DNA blood kit (Qiagen, Hilden, Germany) according to the manufacturer’s instructions.

#### Swedish-Danish case-control cohort

The Swedish-Danish scoliosis case-control dataset consisted of 1739 individuals with idiopathic scoliosis (Scoliosis and Genetics in Scandinavia; ScoliGeneS) and 1812 controls, described in more detail in [15].

#### Japanese case-control cohort

The Japanese case-control dataset consisted of 2,109 cases and 11,140 controls. Japanese AIS subjects were recruited from collaborating hospitals of the Japanese Scoliosis Clinical Research Group and were diagnosed through clinical and radiologic examinations by expert scoliosis surgeons. Control subjects were recruited from the BioBank Japan Project (https://biobankjp.org) and its related projects. It is described in more detail in [16].

#### US case-control cohort

The US case-control dataset consisted of an independent cohort of 9,312 Caucasian subjects (1,360 cases and 7,952 controls). Case subjects were recruited at Texas Scottish Rite Hospital for Children in Dallas, Texas as previously described [6] and genotyped using Illumina HumanCoreExome Beadchip array. Informed consents were obtained from all research subjects. The study was approved by the Institutional Review Board of the University of Texas Southwestern Medical Center. For controls, we utilized a single dataset of individuals downloaded from the dbGaP web site [17] from the Geisinger Health System-MyCode, eMERGE III Exome Chip Study under phs000957.v1.p1 (https://www.ncbi.nlm.nih.gov/projects/gap/cgi-bin/study.cgi?study_id=phs000957.v1.p1). The dbGaP controls were previously genotyped on the same microarray platform used for cases. Only subjects self-reported as Non-Hispanic White (NHW) were included in the present study. Phenotypes of all controls were reviewed to exclude any with associated musculoskeletal or neurological disorders.

### Family genotyping & linkage analysis

1 ug of genomic DNA from each of the 15 participating family members was used at the SNP&SEQ genotyping facility in Uppsala, Sweden for genotyping on the Illumina HumanOmniExpressExome-8v1-2 array according to standard protocols (Illumina, San Diego, US) and the results were analyzed using the software GenomeStudio 2011.1 from Illumina. Two controls were also run in parallel. Genotyping was based on cluster files generated from the signal intensities from more than 800 DNA samples processed in parallel to this project. Samples with low (<95%) genotyping rate, markers with low (<99%) success rate, and monomorphic markers were removed. Markers with any inheritance errors within the pedigree, as assessed by Pedcheck [18] were removed from subsequent analysis.

LD-based pruning of the genotype data was performed using the pairwise LD threshold function in PLINK (v1.07, http://www.pngu.mgh.harvard.edu/purcell/plink/, [19]), with a window of 50 markers, shifting by five markers at a time, and allowing the markers within each window to have an r^2^ of max 0.2 (—indep-pairwise 50 5 0.2). The Rutgers genetic map interpolator (available at compgen4.rutgers.edu/mapinterpolator), based on the genetic map by Matise *et al*. [20], was used to attain genetic map information for the markers in the linkage analysis. The data (genotypes, pedigree information, phenotype information) was stored in BCGenome, a genome data integration platform from BC Platforms (Espoo, Finland). Merlin (v1.1.2, available at csg.sph.umich.edu/abecasis/Merlin/index.html, [21]) was used to perform non-parametric linkage analysis, using the exponential model and assessing sharing of genetic regions between all affected individuals. Merlin was also used to predict the most likely haplotypes within the region of interest on chromosome 1.

### Exome sequencing

Two individuals, heterozygote carriers of the putative chromosome 1 risk haplotype, were chosen for exome sequencing. 120ng of genomic DNA was used to prepare AmpliSeq libraries run on Ion Torrent (Life Technologies, USA) sequencing according to standard protocols. The sequencing was performed at the Uppsala Genome Center, Uppsala, Sweden. The sequence reads were aligned to the hg19 genome reference assembly using the Ion Proton pipeline (Life Technologies, USA) and single nucleotide variants (SNVs) were identified in each sample using the Torrent Suite Software (Life Technologies, USA).

The list of variants was filtered to retain only non-synonymous/splice/stop variants with a read depth of at least 10x. We also removed any variant with a frequency of 1% or higher in the 1000Genomes dataset (either all populations or Europeans, http://browser.1000genomes.org/, [22]) or the ExAC dataset (all populations or Europeans, exac.broadinstitute.org, [23]).

Based on our linkage and haplotype analysis, we further limited our analysis to variants shared by both exome-sequenced individuals as heterozygous, and located within the linked region on chromosome 1 (GRCh37: 1:84,722,102–113,819,478). Finally, the sequence quality underlying any such variants was manually assessed using the IGV browser (http://www.broadinstitute.org/igv/, [24]) to eliminate false-positive variant calls due to e.g. read-end artifacts.

### Bioinformatic analysis

The tissue expression of any genes carrying putative candidate scoliosis risk variants was assessed through the FANTOM5 promoterome browser available at http://fantom.gsc.riken.jp/zenbu/, [25]. The potential effects of the identified variant were assessed by looking at the SIFT [26], PolyPhen [27], MutTaster [28] and CADD [29] scores of each variant. The conservation of the variant was also assessed through the prediction of GERP-scores. These analyses were performed through the ANNOVAR pipeline [30].

To analyse the effects of the p. V2287I mutation in *CELSR2*, we compared the predicted 3D structure of the protein either with a Valine (V) or Isoleucine (I) at position 2287 of the protein. We used the RaptorX tool (available at raptorx.uchicago.edu [31]) for 3D structure predictions with default parameters.

A Taqman assay C_166310564_10 from Life Technologies (Thermo Fisher, Waltham, US), following the manufacturer′s instructions, was used to assess the frequency of the rare *CELSR2* variant, identified through exome sequencing of the family, in a previously described cohort of Swedish-Danish individuals with idiopathic scoliosis [15].

### Genotyping of common *CELSR1*, *2* and *3* variants in a Swedish-Danish case-control cohort

The Agena (http://agenabio.com/, San Diego, US, previously Sequenom) MassARRAY^®^ System, combined with iPLEX^®^ chemistry, was used to genotype a set of common variants in *CELSR1*, *2 and 3*. The variants were chosen using the LD TAG SNP Selection tool at https://snpinfo.niehs.nih.gov/snpinfo/snptag.php, [32]) to tag each gene with an LD threshold of r^2^ = 0.8. For practical reasons, only the variants fitting into two iPLEX pools were selected. The 20 common variants in *CELSR1-3* were genotyped in the same cohort of Swedish-Danish cases and controls as used previously [15]. The HWE of each marker in controls was assessed, and any sample with <80% call-rate was removed.

### Replication studies of associated *CELSR2* variants

The genotyping and imputation analysis in the Japanese cohort were carried out as previously described [16].

In the US cohort, initial per-sample quality control measures were applied and we excluded sex inconsistencies and any with missing genotype rate per person more than 0.03. The remaining samples were merged using the default mode in PLINK.1.9 [33]. Duplicated or related individuals were removed as described in [34]. We used principal component analysis (PCA) [35] on the merged data projected onto HapMap3 samples as recommended by [36] to correct for possible stratification. We used PLINK for per-SNPs quality control including genotyping call-rate per marker (>95%) and deviation from Hardy-Weinberg equilibrium (cutoff p-value = 10^−4^). To check the association for common SNPs around the *CELSR2* gene we performed genotype imputation using minimac3 [37] with the 1000G-Phase3.V.5 reference panel as described in the instructions provided by the software developer available at (http://genome.sph.umich.edu/wiki/Minimac3_Imputation_Cookbook). Only common SNPs (MAF>0.05) with imputation quality r^2^ >0.3 were included for further analysis. Genetic association for the imputed allele dosages for the region around the *CELSR2* gene (2Mb each side) was performed by Mach2dat software [38] using logistic regression with gender and ten principal components as covariates.

## Results

### Identification of candidate genomic regions

**Fig 2:**
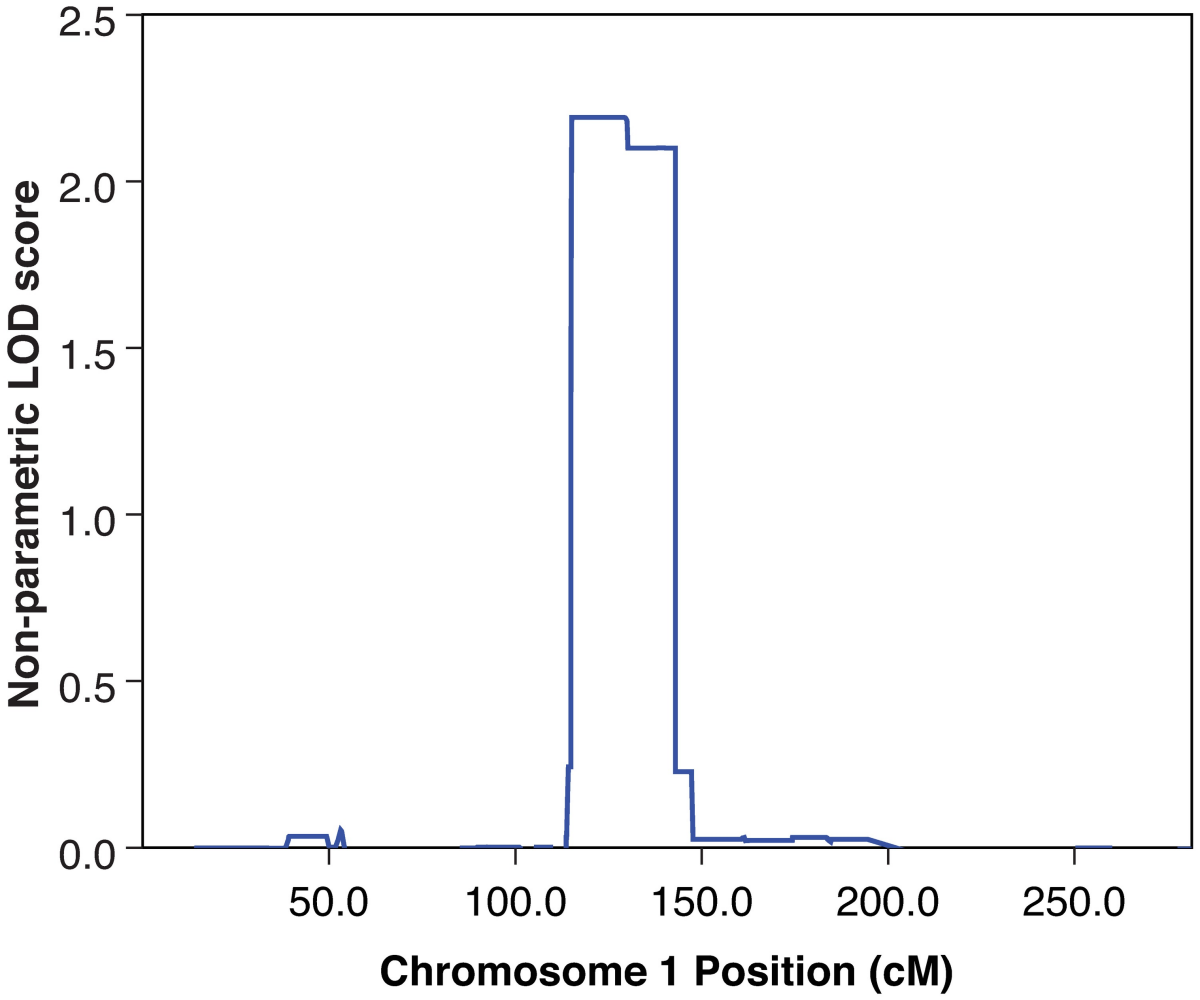
Genetic linkage peak on chromosome 1, indicating sharing in all affected individuals. The maximum K&C non-parametric LOD (NPL) score was attained through the exponential model. The x-axis shows the position on the chromosome (in cM), the y-axis shows the NPL score.

Fifteen family members (Fig 1) were genotyped using the Illumina HumanOmniExpressExome-8v1-2 array (genotyped individuals are marked with Roman numerals). The average genotyping call rate, per SNP with sample call rate > 0, was >99%. The average call rate per sample was >99%. A set of 19,030 pruned-in genetic markers with a 100% call rate was used for the subsequent non-parametric linkage analysis. S1 Fig shows the linkage for all chromosomes. The only region of the genome harboring an NPL linkage signal >1, indicating increased sharing of that chromosomal region by affected individuals, was at ca. 100–150 cM on chromosome 1 (Fig 2). This region thus emerged as the most plausible region harboring a putative scoliosis risk gene/variant, shared by the majority of affected family members.

We found that one haplotype co-segregated with idiopathic scoliosis in the pedigree, as shown in blue in Fig 1 (all other haplotypes are marked as white) and in further detail in S2 Table. This putative risk haplotype was found in one copy in all affected individuals, as well as in unaffected individuals I:II, I:IV and II:VII as shown in Fig 1, and most likely explains the linkage signal we identified. Based on this analysis, the putative disease variant was hypothesized to lie between 84,8–113,8 Mb (Genome Reference Consortium version 37, GRCh37), yielding a candidate region of circa 30Mb containing approx. 300 known genes. We restricted our subsequent search for scoliosis risk variants to this genomic region.

### Exome sequencing analysis & *CELSR2*

We exome sequenced II:I and II:III, two members of the pedigree who shared one copy each of the putative risk haplotype on chromosome 1, in order to look for shared rare variants within the haplotype. We obtained >35M reads from each individual, with >95% on target and an average base coverage depth of >100, >98% of target bases covered at least 1x, and >57M bases in target regions (S2 Fig).

In total, 64,948 variants were called in the exome analysis; the full set of variants is available as S1 Data. Of these, 40,818 were found in both individuals. 40,291 variants were autosomal, and 16,290 variants were exonic/affecting splice sites. Of these, 7162 variants were predicted to result in stop codon loss or gain, or non-synonymous changes and 61 of them were located within the region of linkage on chromosome 1. Filtering by frequency (allowing <1% minor allele frequency in 1000Genomes ALL/EUR or ExAC ALL/EUR), only three variants remained. Two of these variants were further excluded as being highly likely technical artifacts (S3 Fig).

The final filtered list of high-quality non-synonymous variants within the linkage region on chromosome 1 thus consisted of only one single-nucleotide variant. This finding was confirmed by Sanger sequencing (S4 Fig shows individual I:III (wildtype) and I:I (heterozygote carrier)). This variant was rs141489111, a non-synonymous variant resulting in a c.G6859A (NM_001408) change in exon 21 of the cadherin EGF lag seven-pass G-type receptor 2 (*CELSR2*) gene. Rs141489111 is predicted to result in a p.V2287I (NP_001399.1) change in the protein.

This variant was found on the genotyping array used for the initial linkage analysis. The co-segregation of this variant and the chromosome 1 risk haplotype with scoliosis within the entire pedigree could thus be assessed (green boxes in Fig 1).

The A allele of rs141489111 is rare; it is not found in any of the 1000 Genomes populations and it has a frequency of only 0.22% in the NHLBI-ESP European American Cohort (http://evs.gs.washington.edu/EVS/, Exome Variant Server, NHLBI GO Exome Sequencing Project (ESP), Seattle, WA, accessed 26.01.2017). It has a frequency of 0.1477% in the ExAC (http://exac.broadinstitute.org/, accessed 03.07.2017) non-Finnish European population, and 0.086% in the whole dataset. The frequency of this variant in the GnomAD database (http://gnomad.broadinstitute.org/, accessed 03.07.2017) is 0.1643% in non-Finnish Europeans and 0.089% in the whole dataset. The putative p.V2287I change replaces a hydrophobic side chain by a larger one. The SIFT and MutTaster scores (1 and 0.96, respectively) do not argue for this variant being directly damaging to the CELSR2 protein, whereas the PolyPhen score of 0.36 is more indeterminate and the CADD score of 22 indicates that this variant might be damaging. The rs141489111 GERP score of 4.06 indicates considerable conservation of this amino acid, arguing for a functional restraint on its function. S5 Fig shows clustalX alignments (available at http://www.uniprot.org) of the amino acids 2261–2320 of Q9HCU4 (human CELSR2) compared to those of several other vertebrate animals. Position 2287 in the human CELSR2 is marked in red. This position is strongly conserved, as well as the adjacent region.

**Fig 3:**
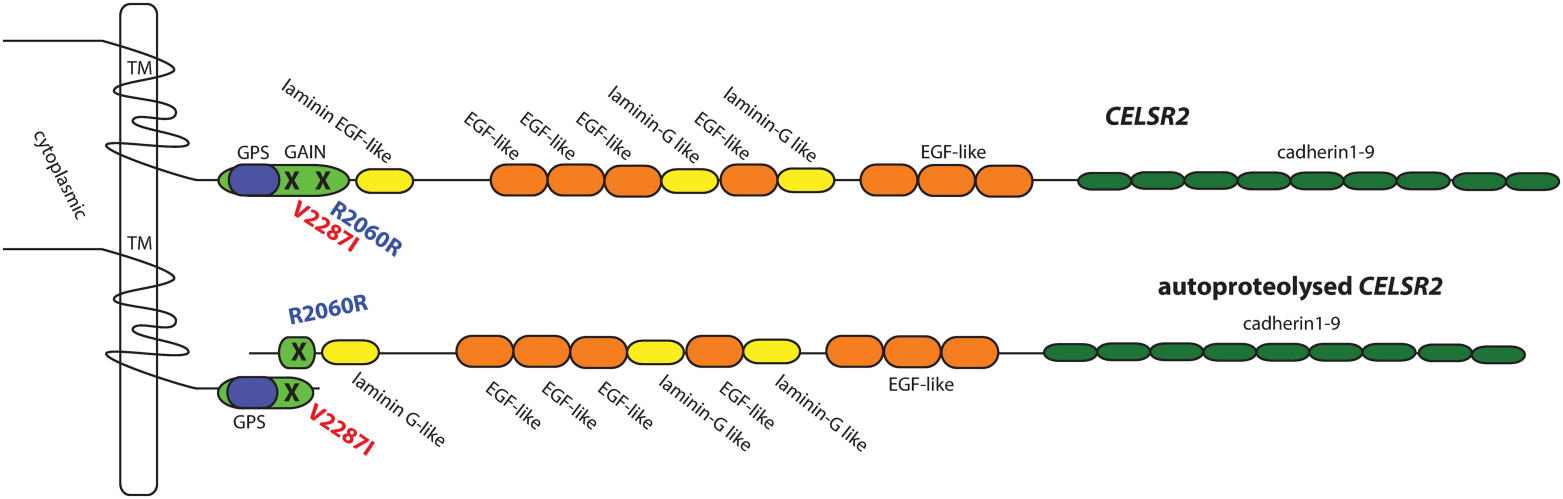
Schematic of the structure of the *CELSR2* protein. Shown are the cadherin, EGF-like, and laminin-G-like domains. Also the transmembrane (TM) 7-pass domain and the evolutionarily conserved GAIN domain. The GAIN domain contains within it a GPS domain and is the site of autoproteolytic cleavage of *CELSR2*. The rare V2287I variant and more common R2060R tagging variant are both located within the GAIN domain.

**Fig 4:**
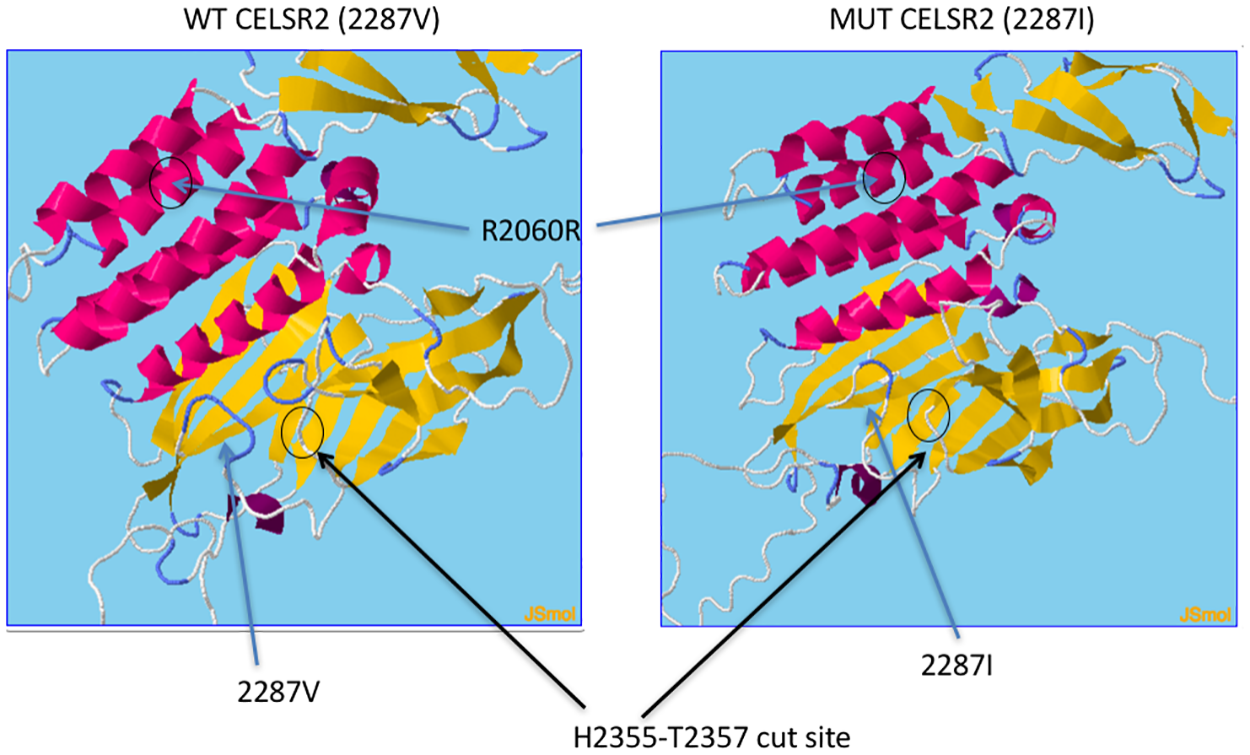
Visualisation of the predicted structure of the GAIN domain of *CELSR2*. The left panel shows the wildtype 2287V form, the right panel shows the mutated 2287I form. The location of the V2287I and R2060R variants are shown, as well as the site of autoproteolytic cleavage.

The V2287I variant is located within the ancient and highly conserved GAIN domain [39] (Fig 3). Fig 4 shows the predicted three-dimensional structure of the GAIN domain of CELSR2 (pdb-files available on demand). While the V2287I change is not expected to have major effects on the structure of the protein, the predicted structure of CELSR2 indicates that it is located in close proximity to the H2355-T2357 autoproteolysis cut site [39].

### Genotyping of rare *CELSR2* variant in an independent cohort

1737 Swedish individuals with idiopathic scoliosis [15] were successfully genotyped for the *CELSR2* rs141489111 variant (genotyping success rate of >99%). We identified five independent carriers of the rs141489111 variant, including the proband of the Swedish large family. This equates to an allele frequency of 0.1439% in this case cohort. We found no indication that the four carriers were directly related to the family under study or to each other.

### Genotyping of common *CELSR1-3* variants in an independent cohort

To further investigate *CELSR2* and its related genes *CELSR1* and *CELSR3*, we selected a set of twenty common tagging variants within these genes. Removing any samples with a genotyping success rate <80% yielded data for 1731 cases and 1783 controls.

No significant association was found to any of the variants in *CELSR1* or *CELSR3*, but two variants in *CELSR2* showed significant association at the p<0.05 level (Table 1 and S6 Fig). The C allele of the *CELSR2* rs6698843 variant was overrepresented in cases (0.571 vs. 0.546), p = 0.0398, OR 1.10 (not significant after Bonferroni-correction for multiple testing). This variant results in a predicted synonymous P1712P in one of the EGF-like domains of CELSR2, as shown in Figs 3 and 4. The A allele of the *CELSR2* rs2281894 variant had a frequency of 0.225 in cases versus 0.188 in controls (p = 0.0001, OR 1.25 (Bonferroni-corrected p = 0.002)). The more strongly associated variant rs2281894 represents a predicted synonymous R2060R within the GAIN domain of the CELSR2 protein (Figs 3 and 4). These two markers are neither in linkage disequilibrium with each other nor with the rare rs141489111 variant identified in the large family.

**Table 1:** Association of tagging variants in *CELSR1*, *2* and *3* to idiopathic scoliosis in a set of Swedish cases and controls.

*GENE*	Chr	Name	b38 pos	Assoc Allele	Case,Control Counts	Case,Control Freq	P value	OR (95% CI)
*CELSR1*	22	rs35364389	46364189	T	544:2904, 550:3026	0.158, 0.154	0.6465	
*CELSR1*	22	rs9615351	46364584	G	837:2611, 858:2708	0.243, 0.241	0.8339	
*CELSR1*	22	rs6007897	46384624	C	552:2912, 542:3030	0.159, 0.152	0.3780	
*CELSR1*	22	rs6008794	46391797	G	546:2910, 547:3025	0.158, 0.153	0.5748	
*CELSR1*	22	rs6008813	46416688	A	861:2597, 867:2711	0.249, 0.242	0.5156	
*CELSR1*	22	rs4823549	46468568	G	1890:1568, 1929:1645	0.547, 0.540	0.5656	
*CELSR1*	22	rs4823554	46475875	C	2392:1038, 2462:1102	0.697, 0.691	0.5506	
*CELSR1*	22	rs9627484	46512158	G	1116:2348, 1150:2428	0.322, 0.321	0.9454	
*CELSR1*	22	rs4823849	46530585	A	2120:1328, 2169:1397	0.615, 0.608	0.5705	
*CELSR1*	22	rs4823561	46533795	A	235:3229, 242:3336	0.068, 0.068	0.9727	
*CELSR1*	22	rs4823850	46535180	G	237:3207, 233:3337	0.069, 0.065	0.5523	
*CELSR2*	1	rs413380	109252404	T	115:3337, 118:3456	0.033, 0.033	0.9444	
*CELSR2*	1	rs10858082	109256099	G	1583:1869, 1587:1979	0.459, 0.445	0.2546	
*CELSR2*	1	rs653635	109263691	T	3105:353, 3196:376	0.898, 0.895	0.6618	
*CELSR2*	1	rs6698843	109264212	C	1965:1479, 1941:1613	0.571, 0.546	0.0398	1.10 (1.00–1.21)
*CELSR2*	1	rs2281894	109267922	A	780:2682, 671:2895	0.225, 0.188	0.0001	1.25 (1.12–1.41)
*CELSR2*	1	rs4970834	109272258	C	2817:627, 2888:676	0.818, 0.810	0.4125	
*CELSR2*	1	rs629301	109275684	T	2691:763, 2746:828	0.779, 0.768	0.2808	
*CELSR3*	3	rs12107252	48653883	T	3053:407, 3153:425	0.882, 0.881	0.8811	
*CELSR3*	3	rs3821875	48660221	C	3064:396, 3164:410	0.886, 0.885	0.9720	

The name of each marker, position (build 38), the allele counts and allele frequencies are shown. Also the P-values as well as the odds ratio (OR) and 95% confidence interval (CI) for markers with P-values < 0.05. All P-values are uncorrected.

### Replication study of common *CELSR2* variants

The two common variants in CELSR2 that showed significant association to idiopathic scoliosis in the Swedish-Danish population, rs6698843 and rs2281894, were further tested for association in two additional case-control datasets. In a genome-wide association study (GWAS) dataset from Japan (previously described in detail in [16]), neither marker showed association to idiopathic scoliosis. Rs6698843 had a p-value of 0.61 and an OR of 0.98; rs2281894 had a p-value of 0.95 and an OR of 1.00. Results from the US cohort did not show association with idiopathic scoliosis for the two common *CELSR2* variants (rs6698843_p-value = 0.839 (OR = 1.01); rs2281894_p-value = 0.465 (OR = 0.96).

## Discussion

Idiopathic scoliosis has proven to be a complex disorder with high genetic heterogeneity. By linkage analysis and exome sequencing of a family with a high burden of idiopathic scoliosis we identified a rare missense mutation in the highly conserved *CELSR2* gene. We suggest a novel causative mechanism in the aetiology of idiopathic scoliosis.

The rare *CELSR2* variant co-segregating with idiopathic scoliosis in the pedigree under study is a previously described variant with a frequency of approx. 0.08–0.16% in the general European population. The position of the rare *CELSR2* rs141489111 variant, as well as the G-protein coupled receptor (GPCR) auto-proteolysis-inducing (GAIN) domain within which it is situated, is strongly conserved throughout evolution, arguing for the functional importance of this protein. Upon genotyping of a large cohort of independent Swedish-Danish idiopathic scoliosis patients, we did not find an elevated prevalence. This variant does thus not alone explain a significant part of idiopathic scoliosis susceptibility at the population level, while it remains a plausible candidate for rare, monogenic forms of scoliosis.

In order to further understand the possible importance of *CELSR2* in idiopathic scoliosis, we genotyped common variants within *CELSR2* and the related genes *CELSR1 and 3*, in a large independent case-control cohort. Interestingly enough, we found an association of idiopathic scoliosis to another variant, rs2281894, also situated in the GAIN domain of *CELSR2*. This association could not be replicated in large cohorts of Japanese and US case-control datasets. It remains unknown if this is due to differences in diagnostic criteria/selection of samples or perhaps due to population differences in the underlying genetic risk factors. Future studies, aiming to dissect in further detail the overall importance of the *CELSR1-3*/associated genes contribution to scoliosis in multiple populations will be undertaken to understand this further.

*CELSR2* is highly expressed in neuronal tissues, including adult temporal lobe, occipital pole and postcentral gyrus, in addition to whole brain, neurons and fetal and adult spinal chord (as assessed through FANTOM5). In *CELSR2*-deficient mice, the development and planar organization of ependymal cilia are compromised, leading to fatal hydrocephalus [40].

Compound heterozygosity for mutations in *CELSR2* have recently been suggested to cause Joubert syndrome, a ciliopathy disorder, in a young girl [41]. This girl showed a phenotype of cortical heterotopia, microophthalmia, severe growth retardation, cone-shaped epiphyses and growth hormone deficiency. This phenotype is obviously more severe than idiopathic scoliosis and supports the notion that a drastic change in the function of CELSR2, e.g. a gain-of-function due to the mutation, would likely be lethal or cause a more severe phenotype than idiopathic scoliosis. Based on this, it seems more plausible that the mutation identified in the current family would involve a loss of function, with heterozygote carriers having approximately half of the wildtype levels of functioning CELSR2. This is mirrored by the situation in *ADGRG6* (also called *GPR126*), a gene previously associated to idiopathic scoliosis [4]. The protein product of this gene also carries a GAIN domain, and also needs to be auto-proteolysed to function properly. The *ADGRG6* variants that have been associated to idiopathic scoliosis have been intronic, and presumably only mildly or not directly functional. In contrast, homozygosity for a mutation that severely reduces the auto-proteolytic function of *ADGRG6* has been shown in severe arthrogryposis multiplex congenita [42]. This further argues for the importance of the GAIN domains and supports our idea that any mutations abolishing their function (in *ADGRG6* or *CELSR2*) would likely lead to more severe phenotypes than idiopathic scoliosis.

The penetrance of scoliosis in carriers of the *CELSR2* variant in the present study is incomplete, suggesting additional unknown modifiers of disease risk (genetic or environmental). This is a common finding even in clearly monogenic situations (reviewed in e.g. [43]). Another example of this is the reduced penetrance of *POC5* mutations segregating with idiopathic scoliosis in the families studied by Patten *et al*. [13]. They suggest the possible presence of a second risk variant within the families. Another thing to keep in mind is that idiopathic scoliosis is a continuous variable that we dichotomise, and that the difference between a classification as unaffected and mild scoliosis may be very subtle.

The cellular function of CELSR2 is not fully understood but it is known to be important for axon guidance, neuronal migration, and cilium polarity [44–46]. CELSR2 is located at the plasma membrane and belongs to the flamingo cadherin subfamily [40]). This group of proteins is involved in contact-mediated communication between cells and consists of a large extra-cellular domain, a seven-pass transmembrane domain and a cytoplasmic tail [47]. The rare variant identified in the family induces a change from valine to isoleucine in the extracellular part of the protein, in the so-called GAIN domain. The GAIN domain is required and necessary for autoproteolysis of the protein [39], and while the result of changing this particular valine to an isoleucine remains unclear, it is plausible to suggest a possible effect on this mechanism.

In conclusion, we have identified *CELSR2* as a putative novel risk gene idiopathic scoliosis, and we hypothesise that this effect may be mediated through a disruption of the auto-proteolytic mechanism of the GAIN domain in the CELSR2. Future studies will focus on functional studies to understand the specific mechanisms underlying this disruption.

## Supporting information

S1 Fig Merlin NPL pruned linkage plots. Plots per chromosome, using the linear (black) or exponential (blue) functions. The x-axis indicates position on the chromosome in cM, the y-axis indicates the non-parametric LOD score. Data for “ALL” (sharing between all affected) or “Pairs” (sharing between all possible pairs of affected) is shown.
(PDF)

S2 Fig Ion Torrent pipeline output. The output QC for the exome sequencing of individuals II:I (up101_1) and II:III (up101_2).
(PDF)

S3 Fig A, B, C Screenshot from IGV browser. (software.broadinstitute.org/software/igv/), Figure shows the three putative rare *CELSR2* variants shared by both sequenced individuals (upper: II:I, lower: II:III). Panel A shows a putative 1:109805856, C>T variant, panel B shows a putative 1:109805857, C>G variant. Both reside within the same region of low quality sequence and were deemed to be false positive variant calls. Panel C shows the 1:109812092, G>A variant, confirmed by Sanger sequencing to be a true variant.
(PDF)

S4 Fig Sanger validation. *CELSR2* 1:109812092, G>A variant shown by Sanger sequencing. Upper panel shows the wildtype G/G genotype (in I:III), lower panel shows an A/G heterozygote (I:I).
(PDF)

S5 Fig CELSR2 conservation. Conservation of the CELSR2 protein between humans (HUMAN), mice (MOUSE), rat (RAT), dog (CANLF), CAVPO (guinea pig), OTOGA (small-eared galago), BOVIN (bovine), AILME (giant panda), and rabbit (RABIT). The location of the human V2287 is marked in red.
(PDF)

S6 Fig Tagging variants. Association of tagging variants in *CELSR2* with idiopathic scoliosis in a Swedish case-control dataset. The plot is produced using LocusZoom, available at locuszoom.org. The x-axis shows the position of the variants on the chromosome (in Mb), and relative to *CELSR2* and neighbouring genes. The left y-axis shows the -log of the association. The most strongly associated variant, rs2281894, is marked with a diamond; the colour of the other markers (circles) is determined by their linkage disequilibrium (LD) with rs228194 (based on hg19/1000Genomes Nov2014 EUR). The right y-axis shows the recombination rate in the region as a light blue line.
(PDF)

S1 Table Phenotypes. Phenotypic information on the family members included in the linkage/exome sequencing analysis. Individual numbering is as in Fig 1.
(PDF)

S2 Table Haplotypes. Chromosome 1 Merlin most probable haplotypes flow (haplotypes coded as A, B, C etc, and colour coded for simplicity). Haplotypes are produced using Merlin, according to the most likely pattern of gene flow (—best) and using the—horizontal flag.
(PDF)

S1 Data All exonic variants in II:I and II:III. Compressed variant list, annotated by Annovar, containing all the exonic variants found in either II:I or II:III (or both).
(TXT)
