# Supplementary material for: CELSR2 is a candidate susceptibility gene in idiopathic scoliosis
Source: PLoS One. 2017 Dec 14;12(12):e0189591. doi: 10.1371/journal.pone.0189591 (PMC5730153; doi:10.1371/journal.pone.0189591)
Supplement: S1 Fig — Plots per chromosome, using the linear (black) or exponential (blue) functions. The x-axis indicates position on the chromosome in cM, the y-axis indicates the non-parametric LOD score. Data for “ALL” (sharing between all affected) or “Pairs” (sharing between all possible pairs of affected) is shown. (PDF) [file pone.0189591.s001.pdf]

SUPPLEMENTARY FIGURE 1

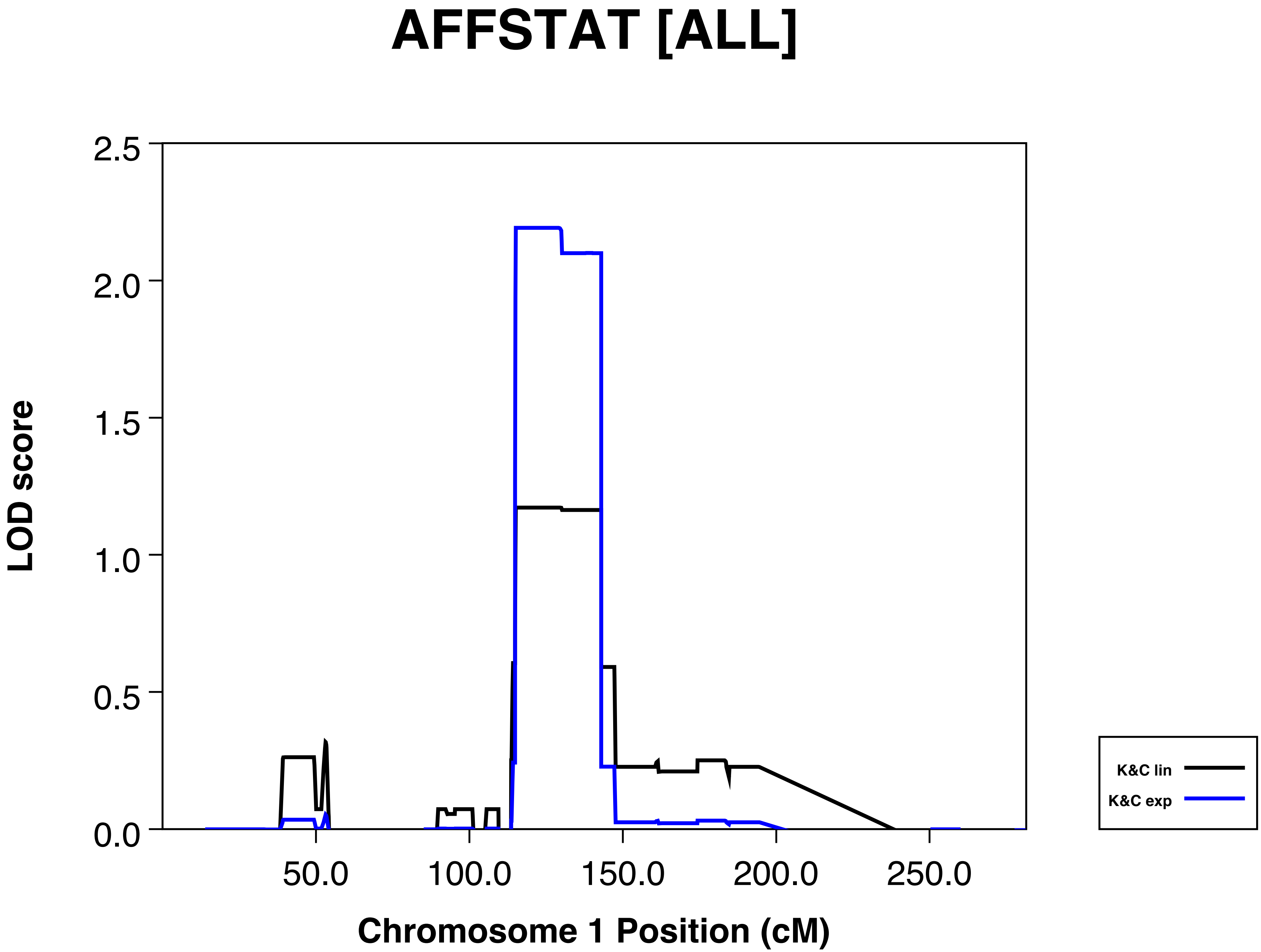

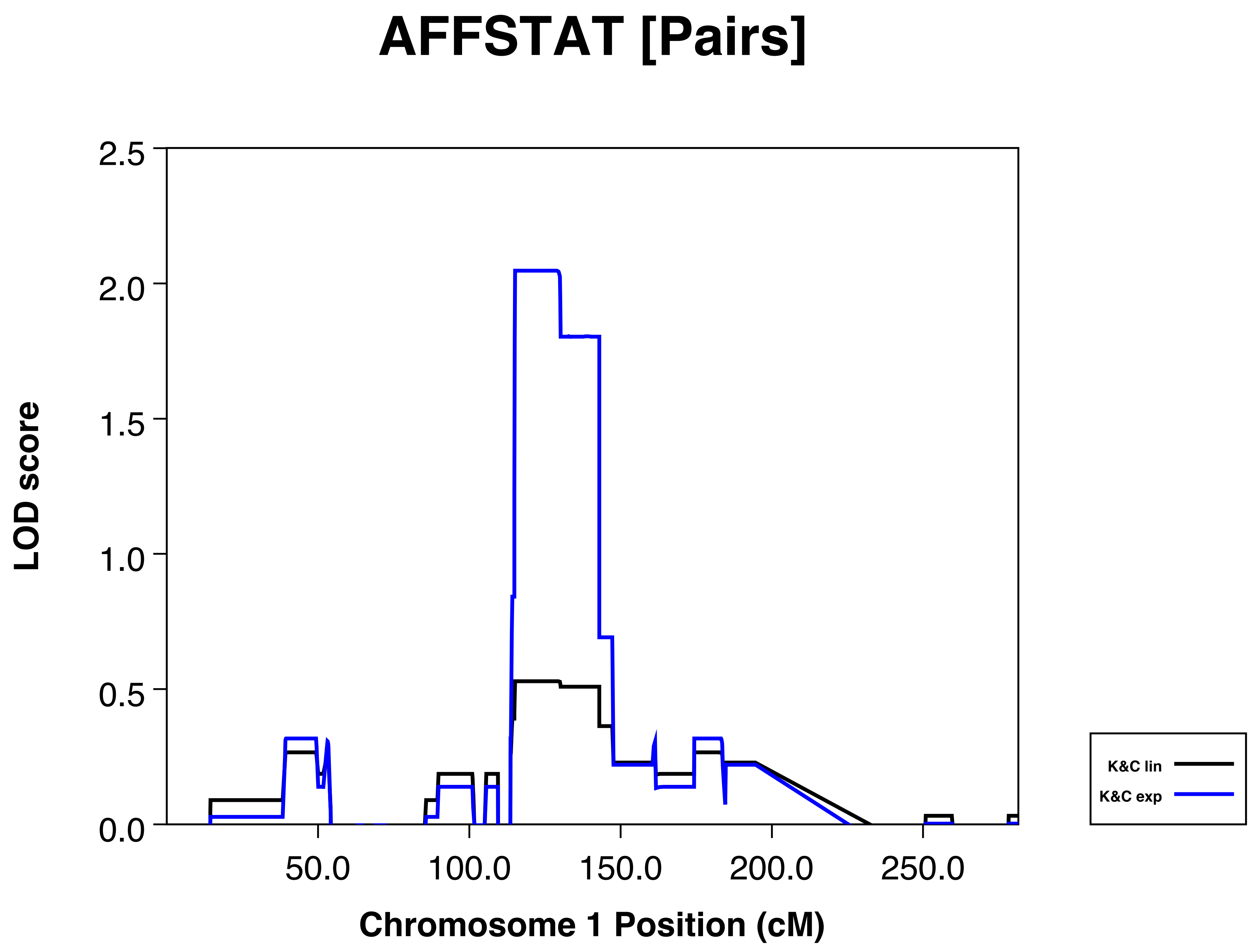

AFFSTAT [Pairs]
LOD score
2.5
2.0
1.5
1.0
0.5
0.0
50.0
100.0
150.0
200.0
250.0
Chromosome 1 Position (cM)
K&C lin
K&C exp

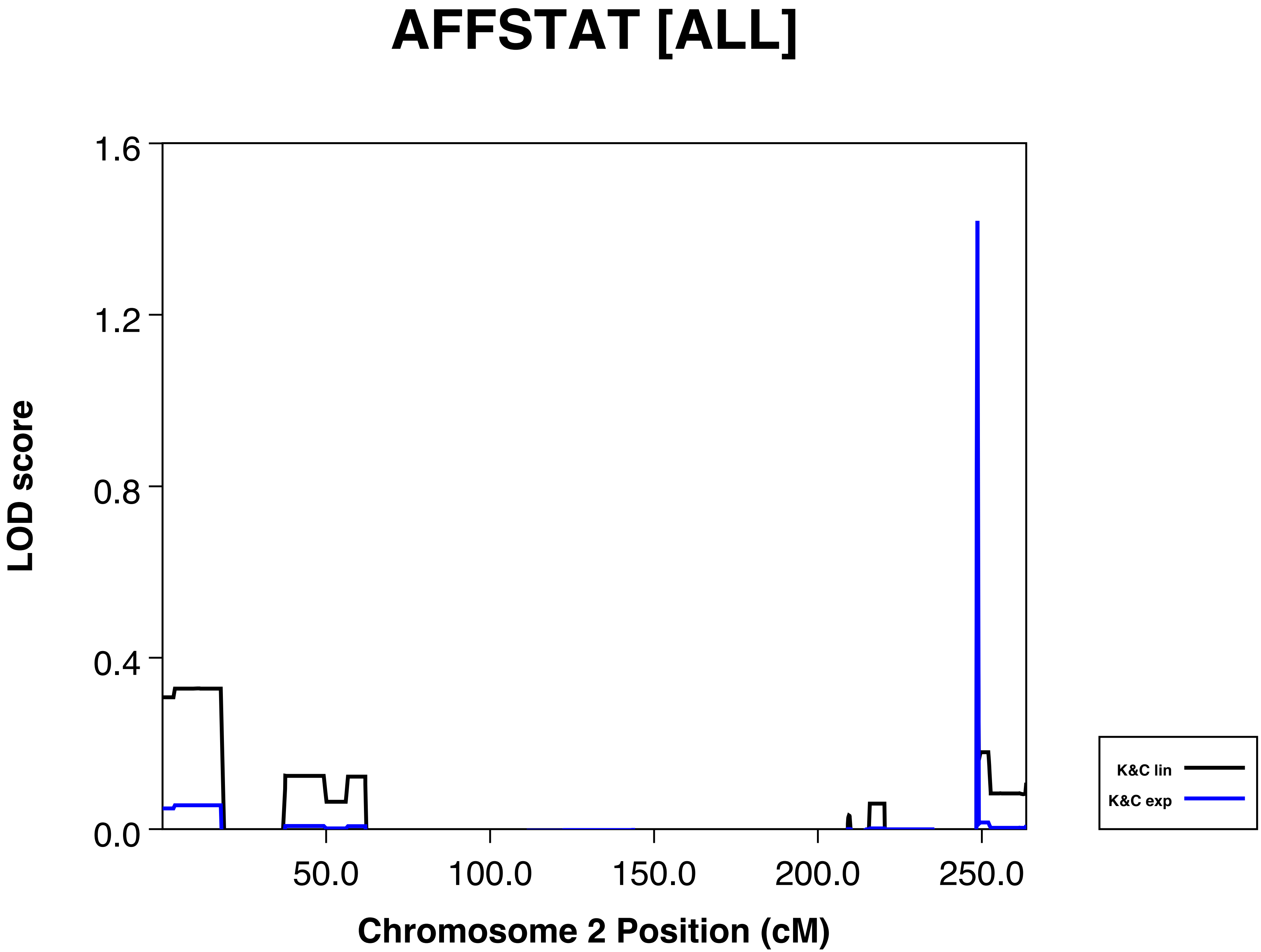

AFFSTAT [ALL]
1.6
1.2
0.8
0.4
0.0
LOD score
50.0
100.0
150.0
200.0
250.0
Chromosome 2 Position (cM)
K&C lin
K&C exp

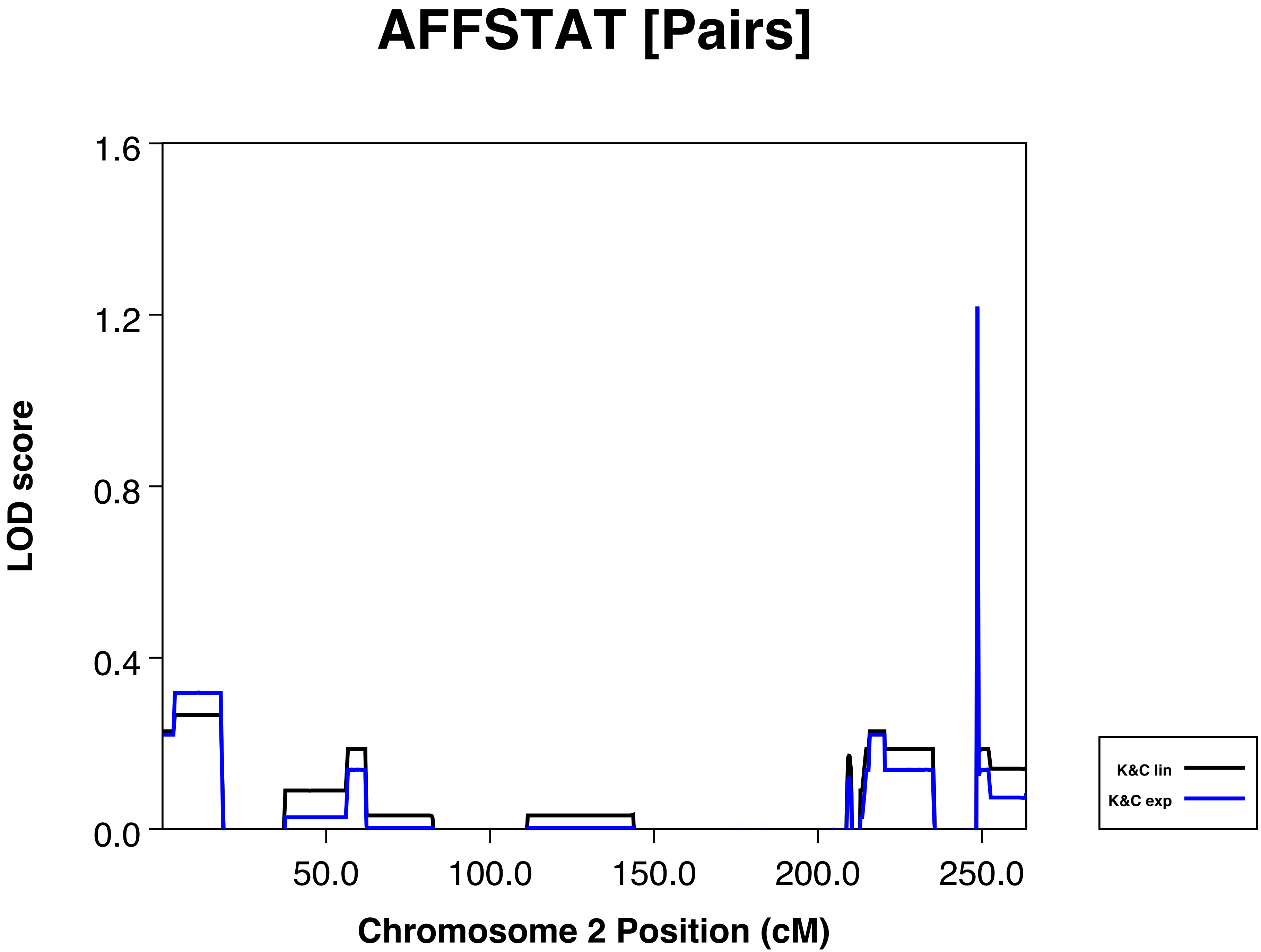

AFFSTAT [Pairs]
LOD score
1.6
1.2
0.8
0.4
0.0
50.0
100.0
150.0
200.0
250.0
Chromosome 2 Position (cM)
K&C lin
K&C exp

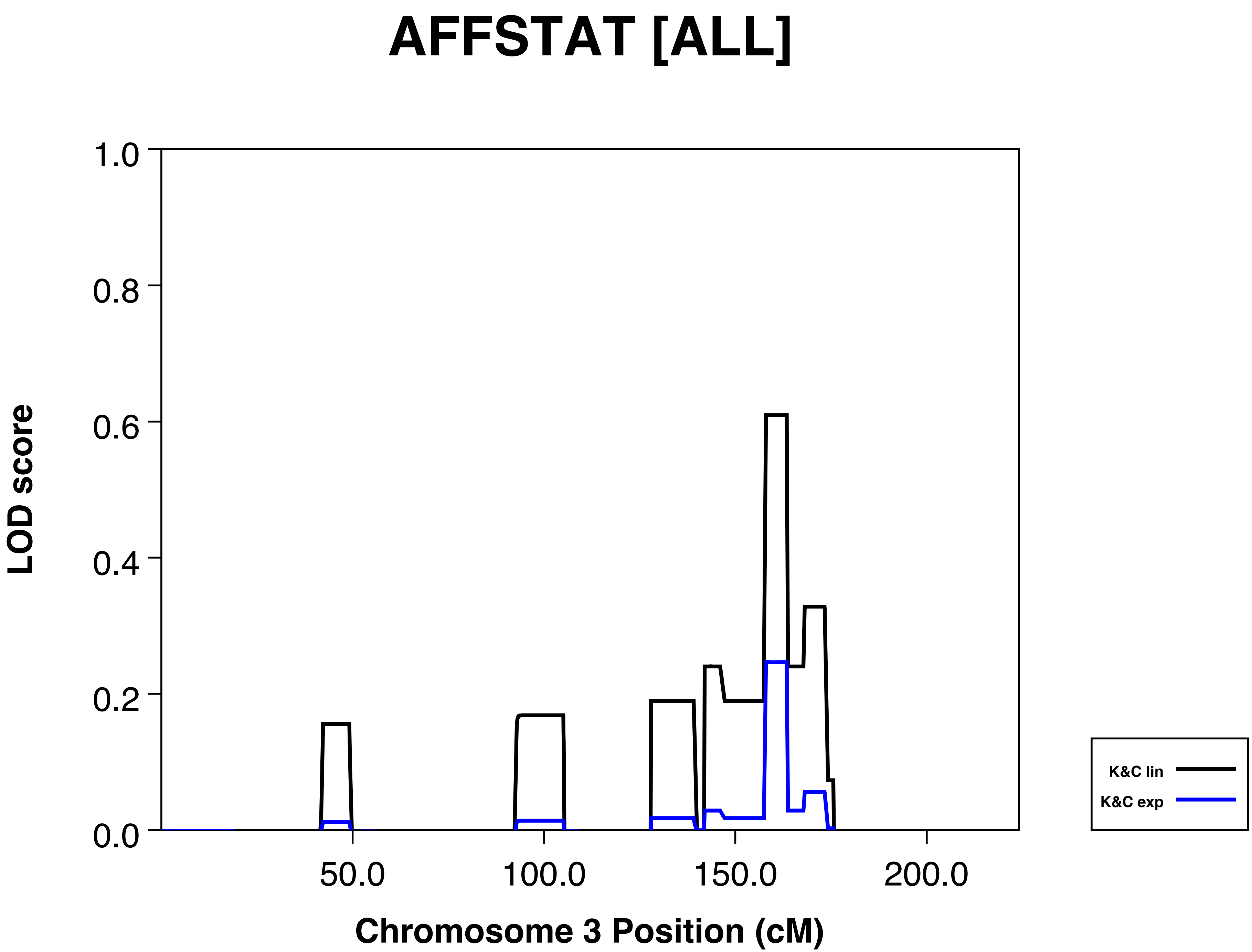
AFFSTAT [ALL]
LOD score
1.0
0.8
0.6
0.4
0.2
0.0
50.0
100.0
150.0
200.0
Chromosome 3 Position (cM)
K&C lin
K&C exp

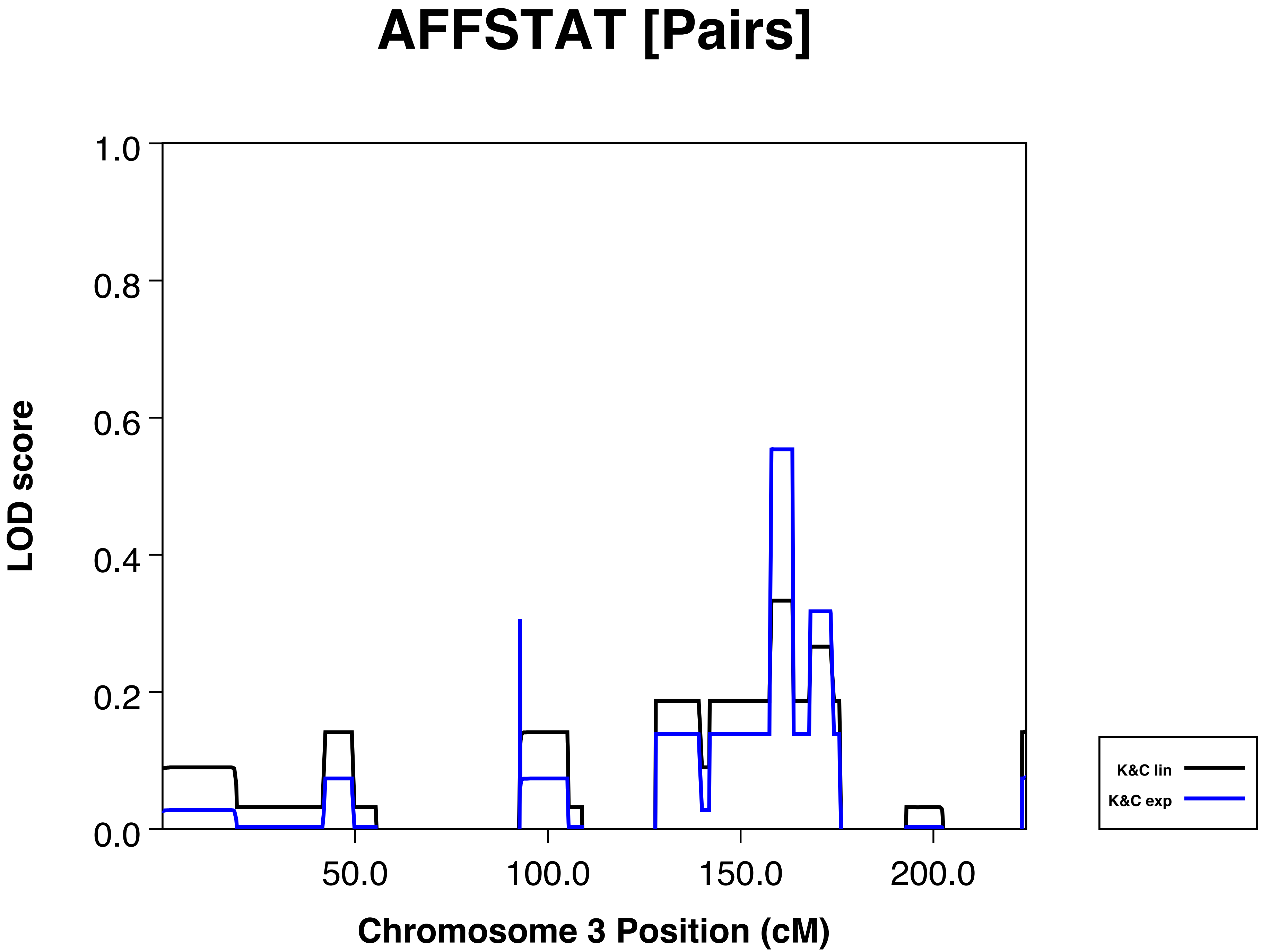

AFFSTAT [Pairs]
LOD score
1.0
0.8
0.6
0.4
0.2
0.0
50.0
100.0
150.0
200.0
Chromosome 3 Position (cM)
K&C lin
K&C exp

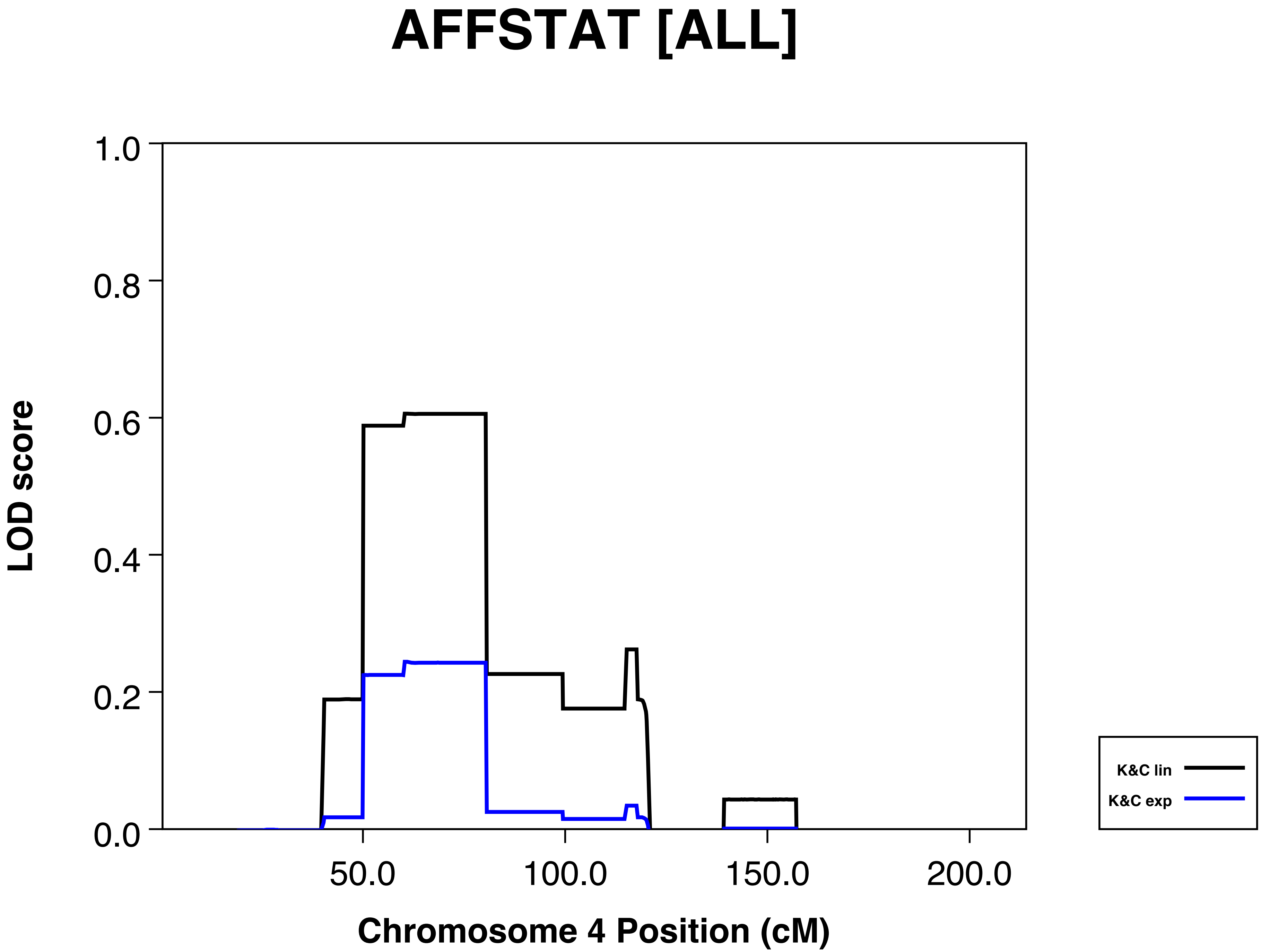

AFFSTAT [ALL]
LOD score
1.0
0.8
0.6
0.4
0.2
0.0
50.0
100.0
150.0
200.0
Chromosome 4 Position (cM)
K&C lin
K&C exp

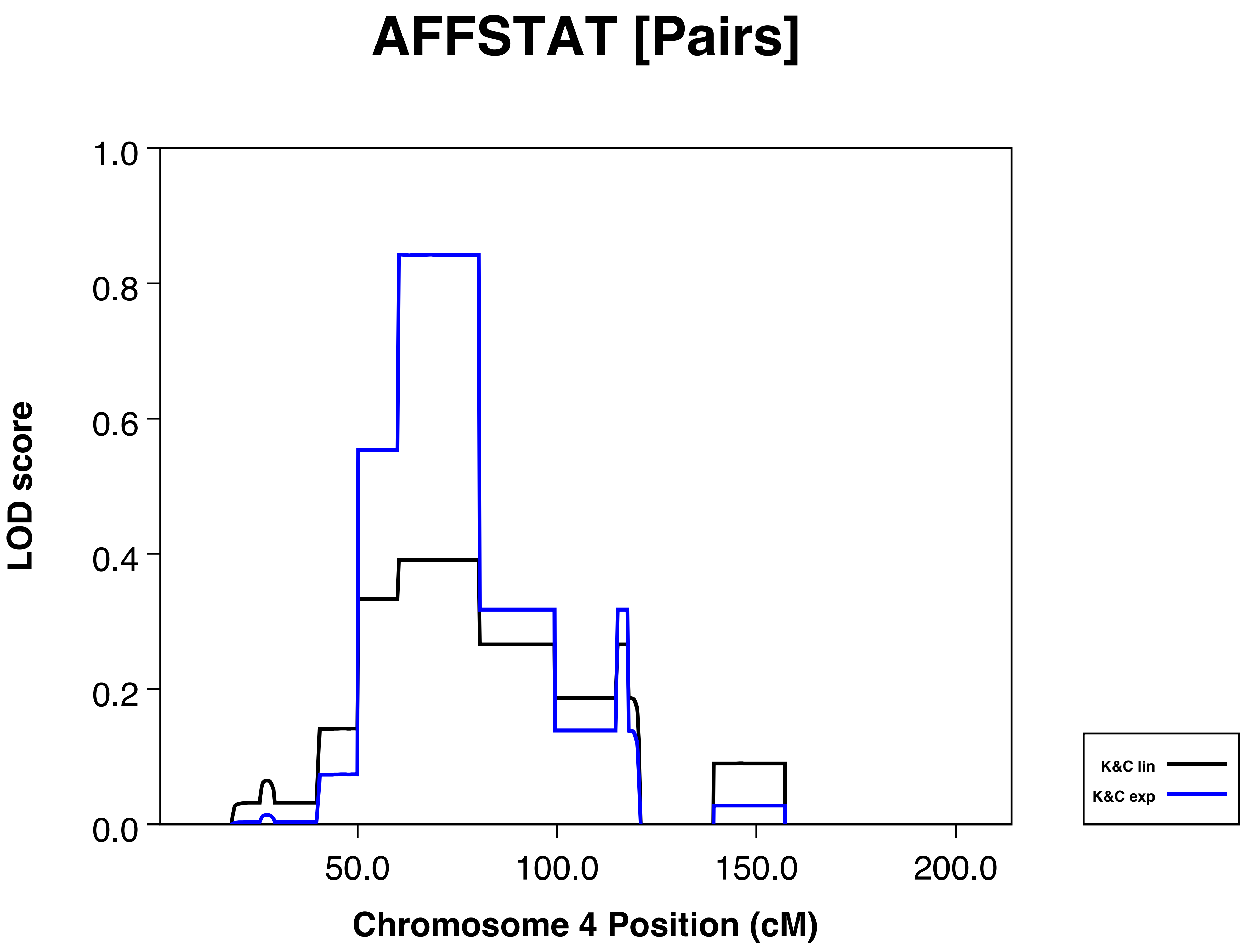

AFFSTAT [Pairs]
LOD score
1.0
0.8
0.6
0.4
0.2
0.0
50.0
100.0
150.0
200.0
Chromosome 4 Position (cM)
K&C lin
K&C exp

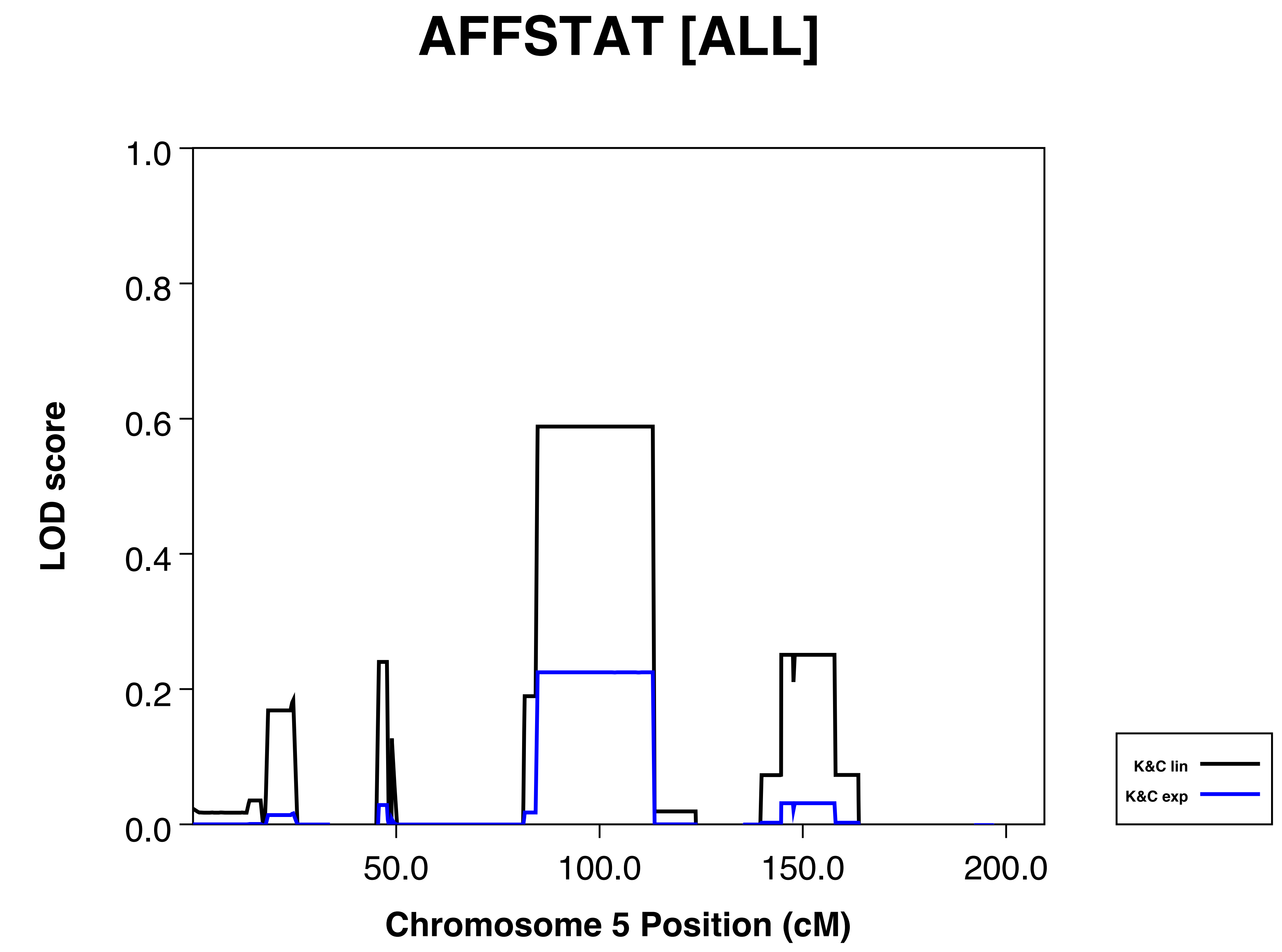

AFFSTAT [ALL]
LOD score
1.0
0.8
0.6
0.4
0.2
0.0
50.0
100.0
150.0
200.0
Chromosome 5 Position (cM)
K&C lin
K&C exp

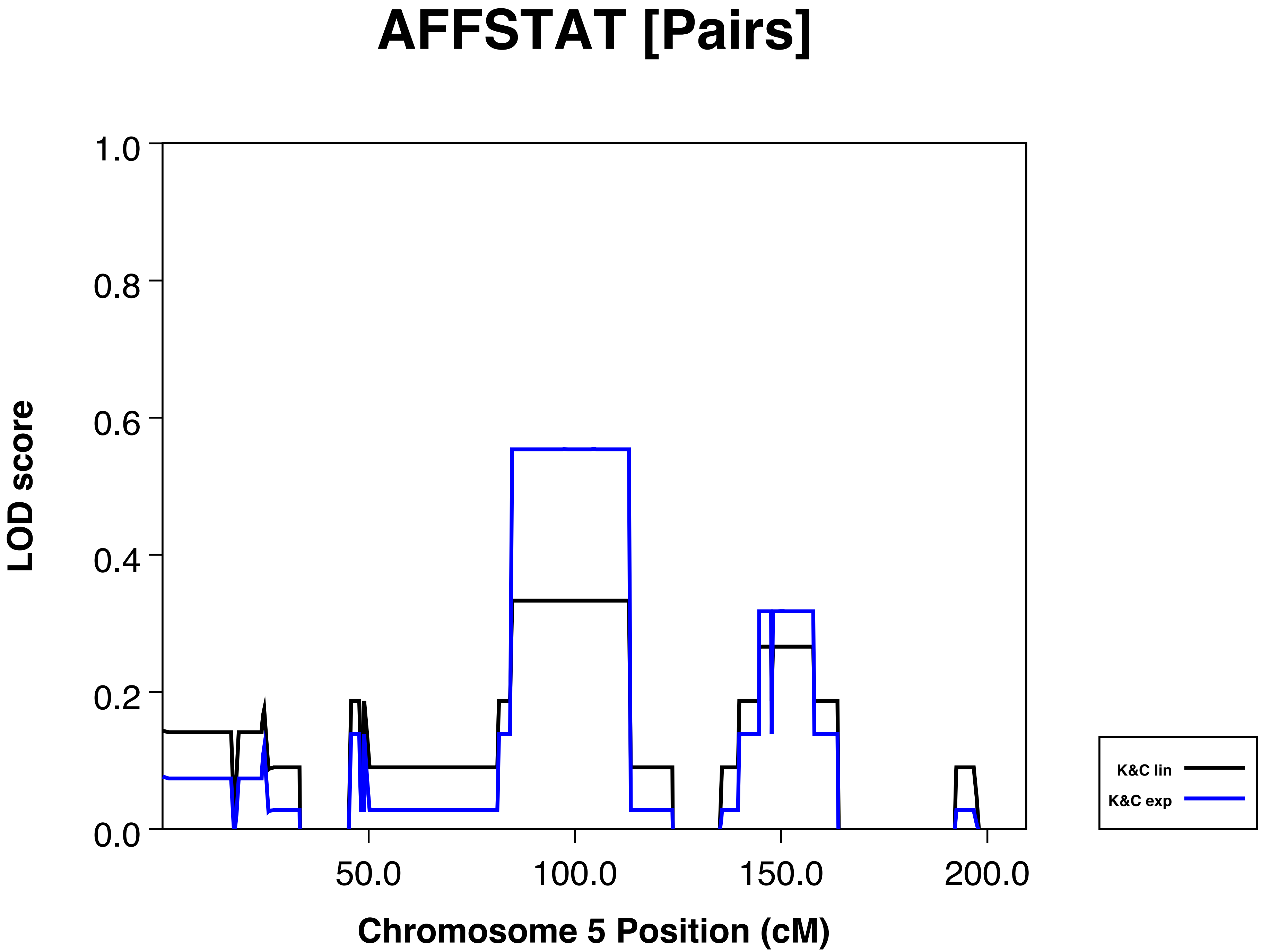
AFFSTAT [Pairs]
1.0
0.8
0.6
0.4
0.2
0.0
LOD score
50.0
100.0
150.0
200.0
Chromosome 5 Position (cM)
K&C lin
K&C exp

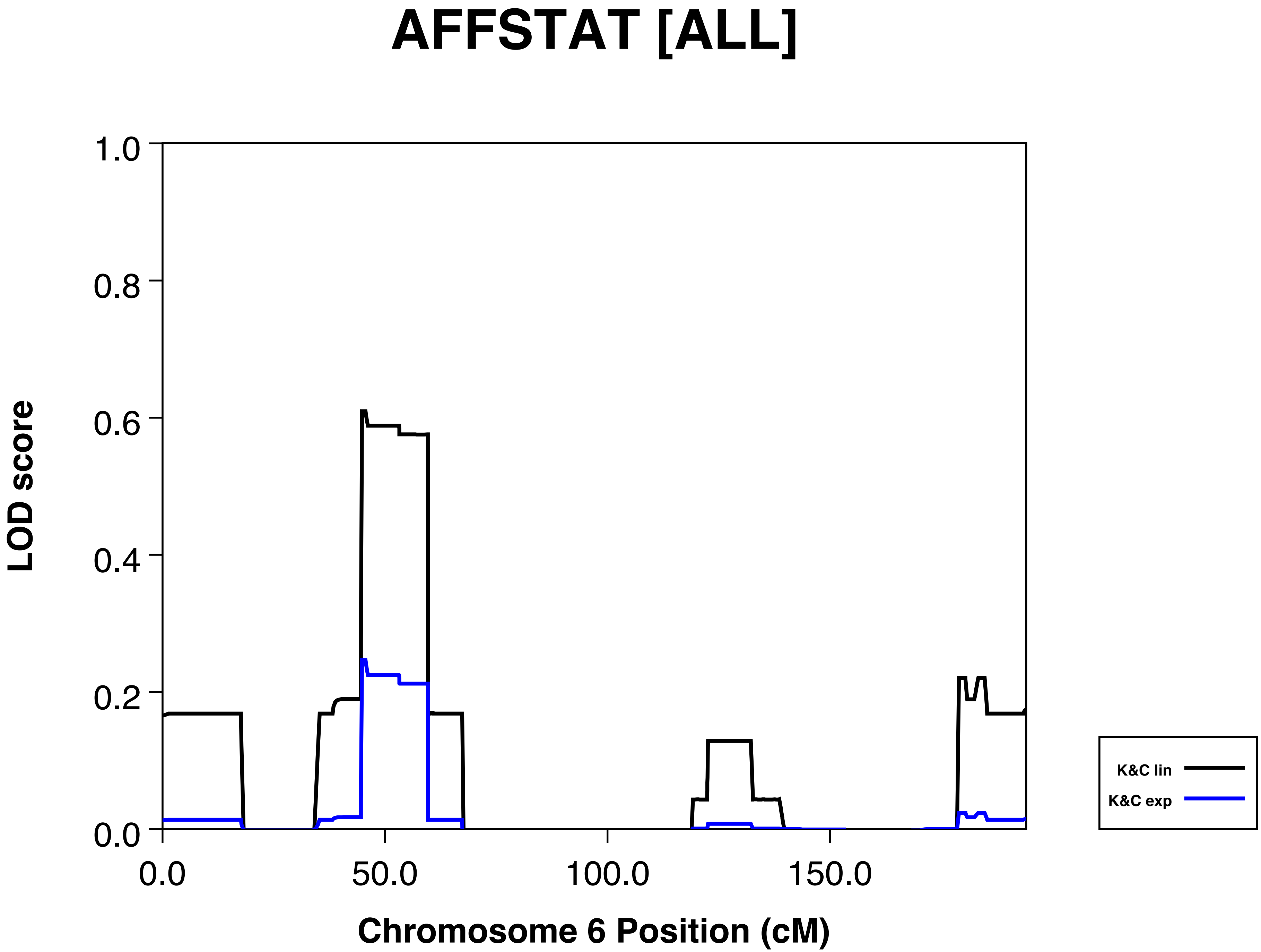
AFFSTAT [ALL]
LOD score
1.0
0.8
0.6
0.4
0.2
0.0
0.0
50.0
100.0
150.0
Chromosome 6 Position (cM)
K&C lin
K&C exp

AFFSTAT [Pairs]

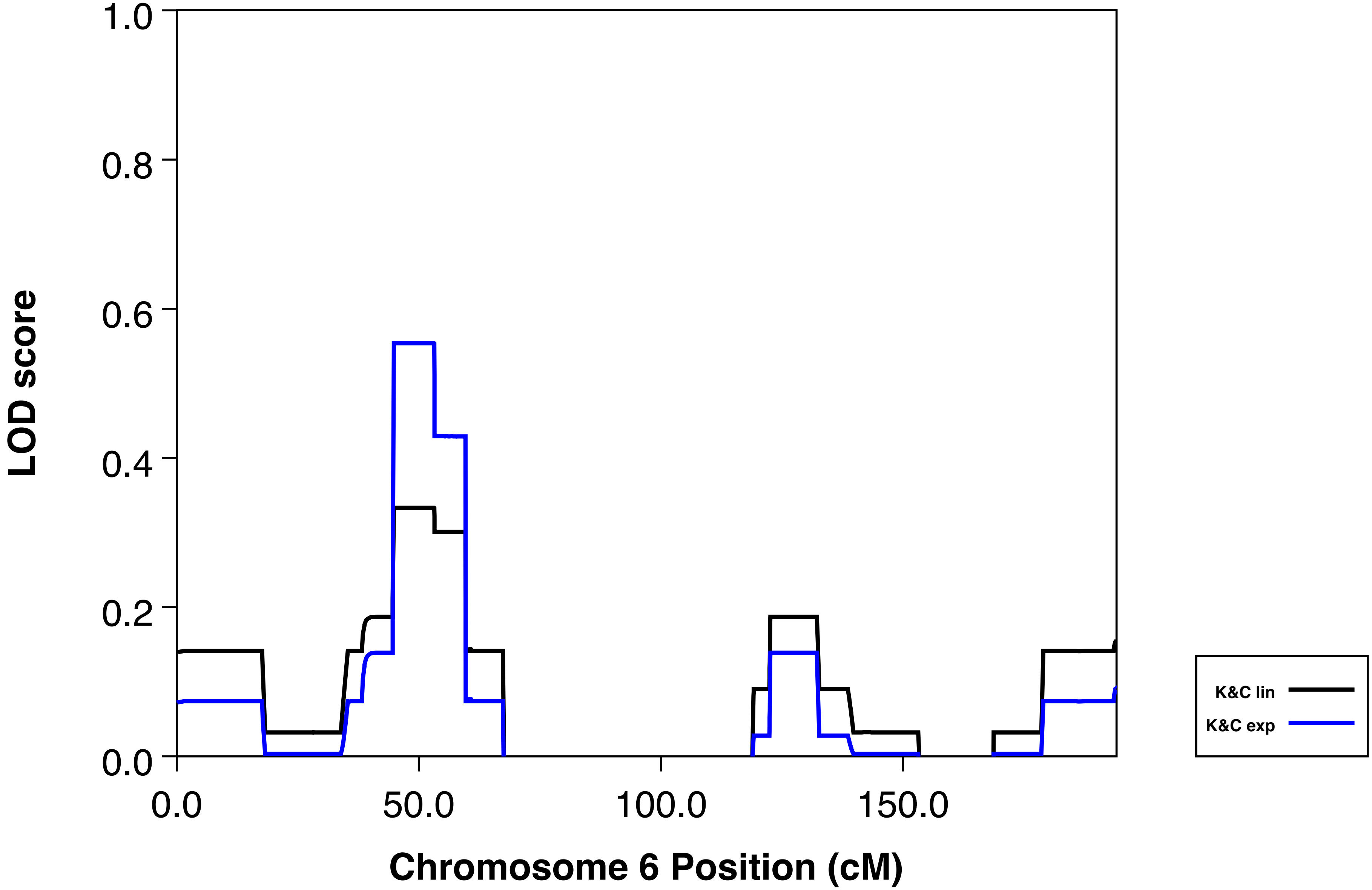

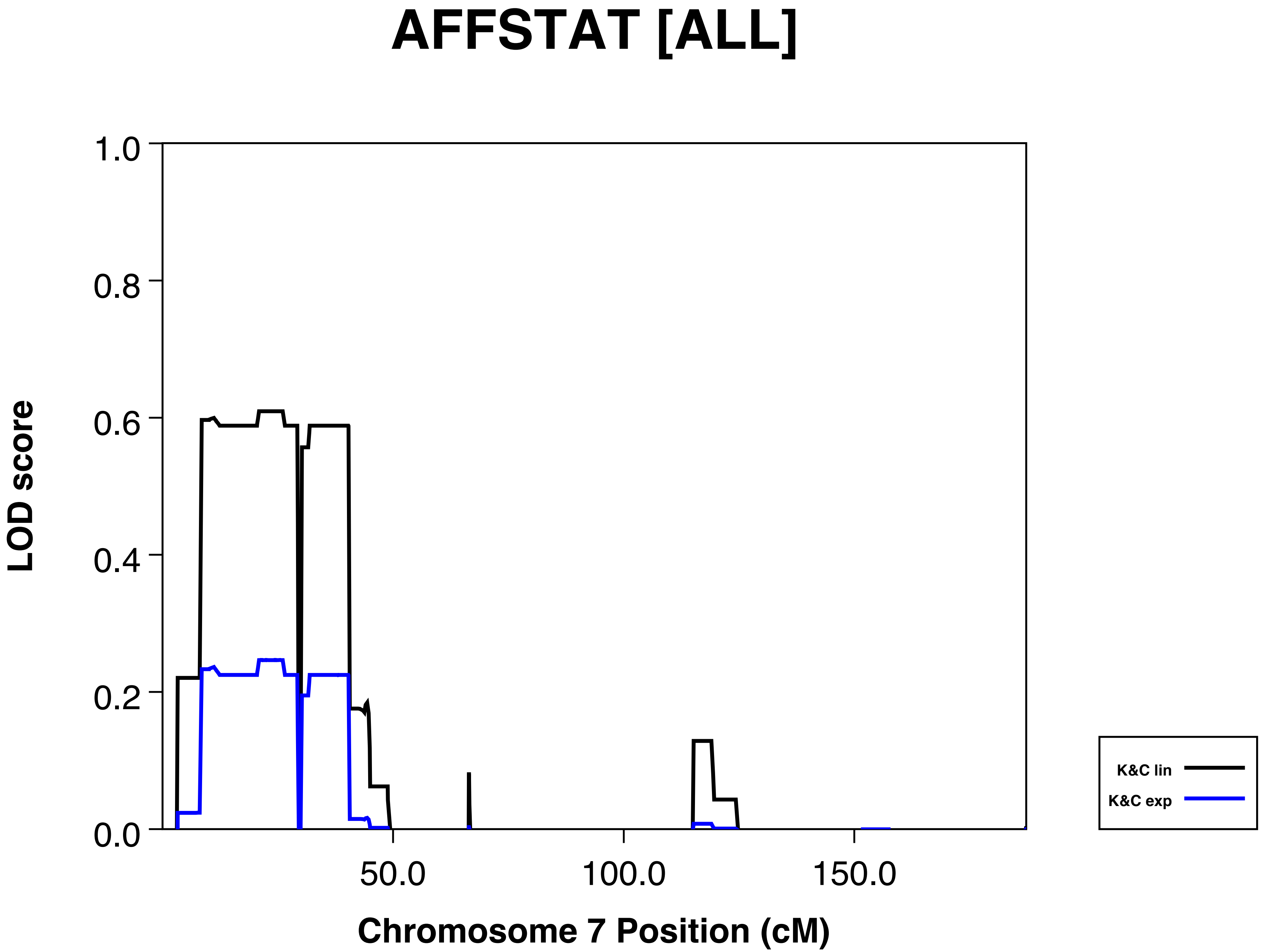
AFFSTAT [ALL]
1.0
0.8
0.6
0.4
0.2
0.0
LOD score
50.0
100.0
150.0
Chromosome 7 Position (cM)
K&C lin
K&C exp

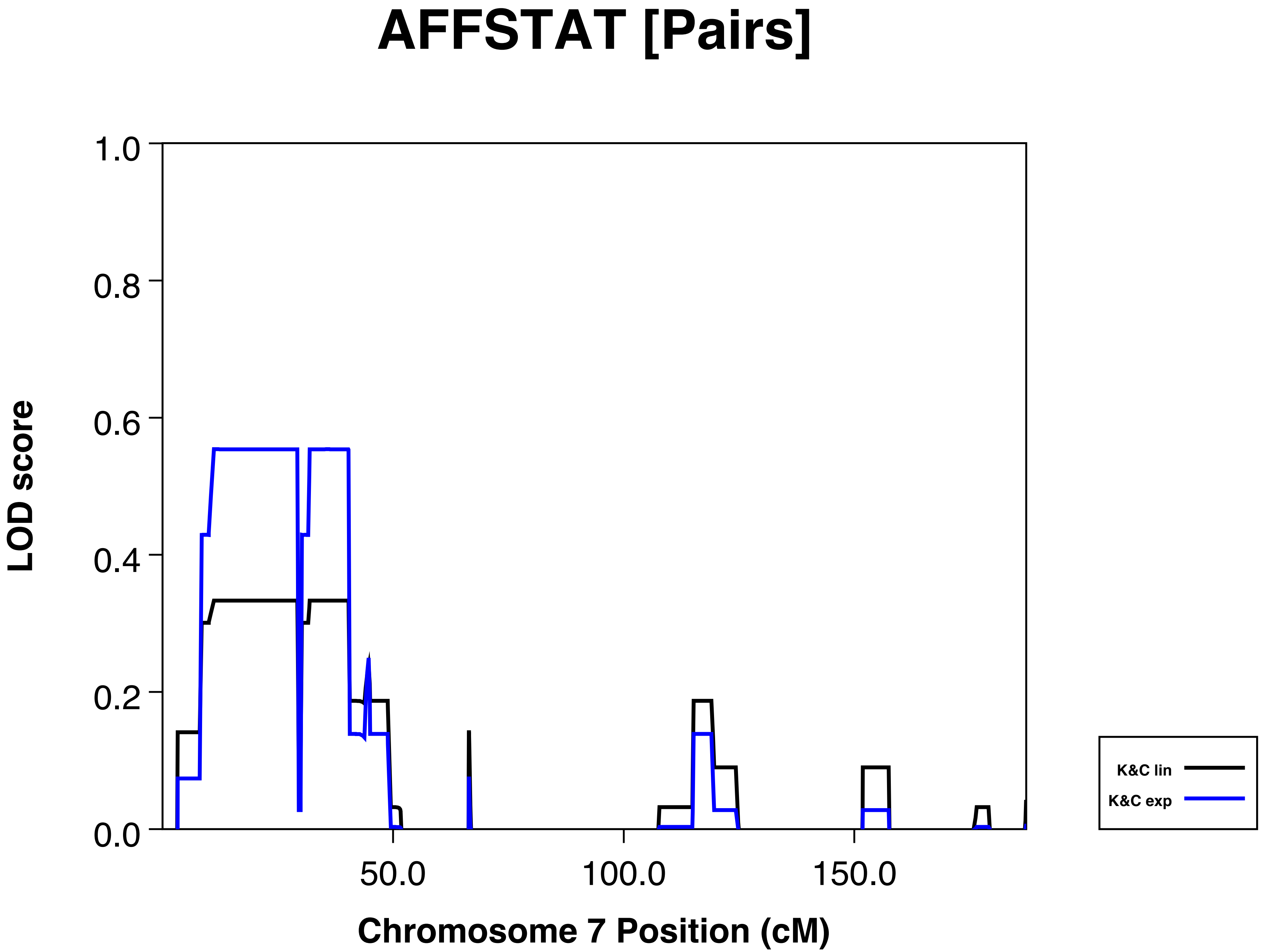

AFFSTAT [Pairs]
1.0
0.8
0.6
0.4
0.2
0.0
LOD score
50.0
100.0
150.0
Chromosome 7 Position (cM)
K&C lin
K&C exp

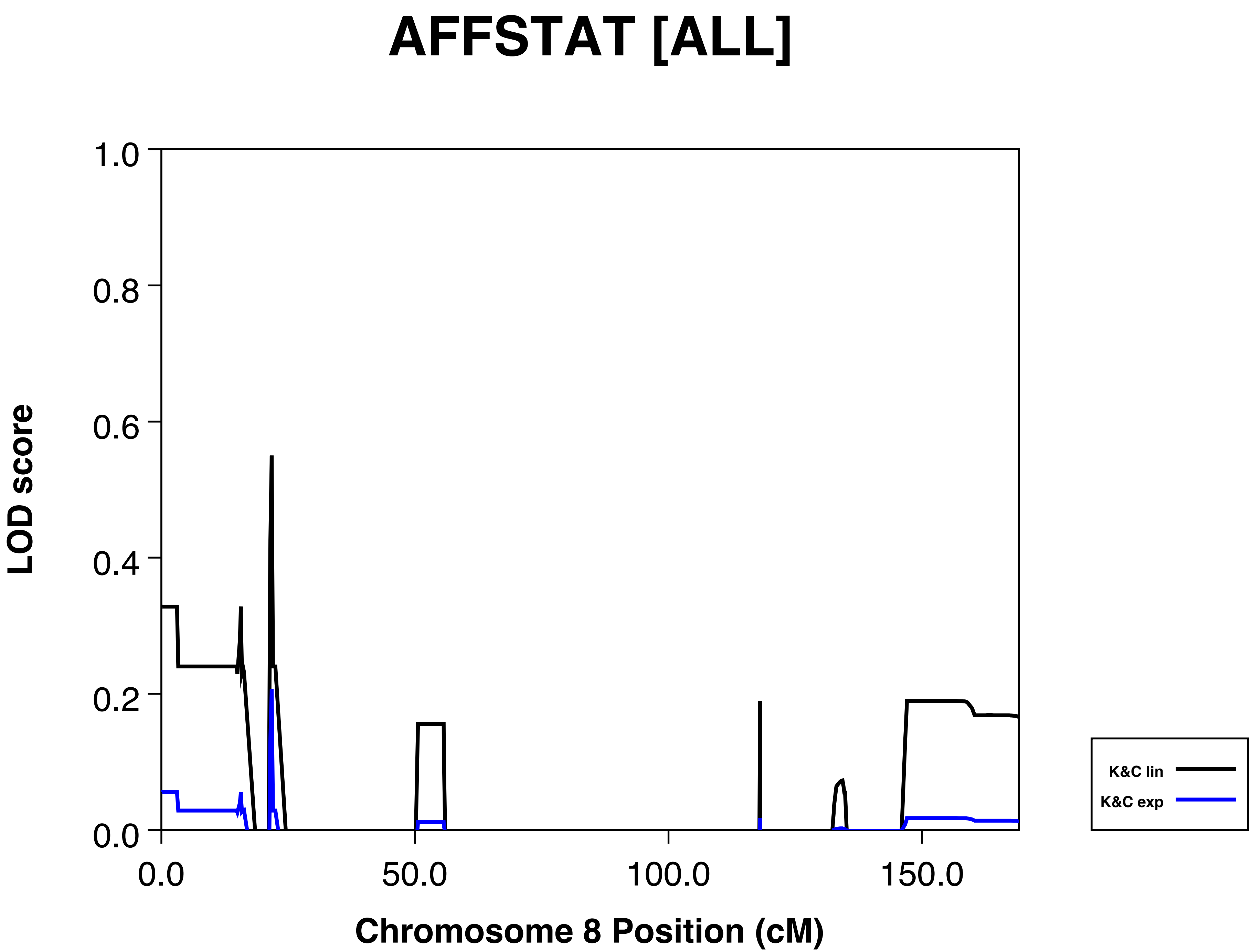
AFFSTAT [ALL]
LOD score
1.0
0.8
0.6
0.4
0.2
0.0
0.0
50.0
100.0
150.0
Chromosome 8 Position (cM)
K&C lin
K&C exp

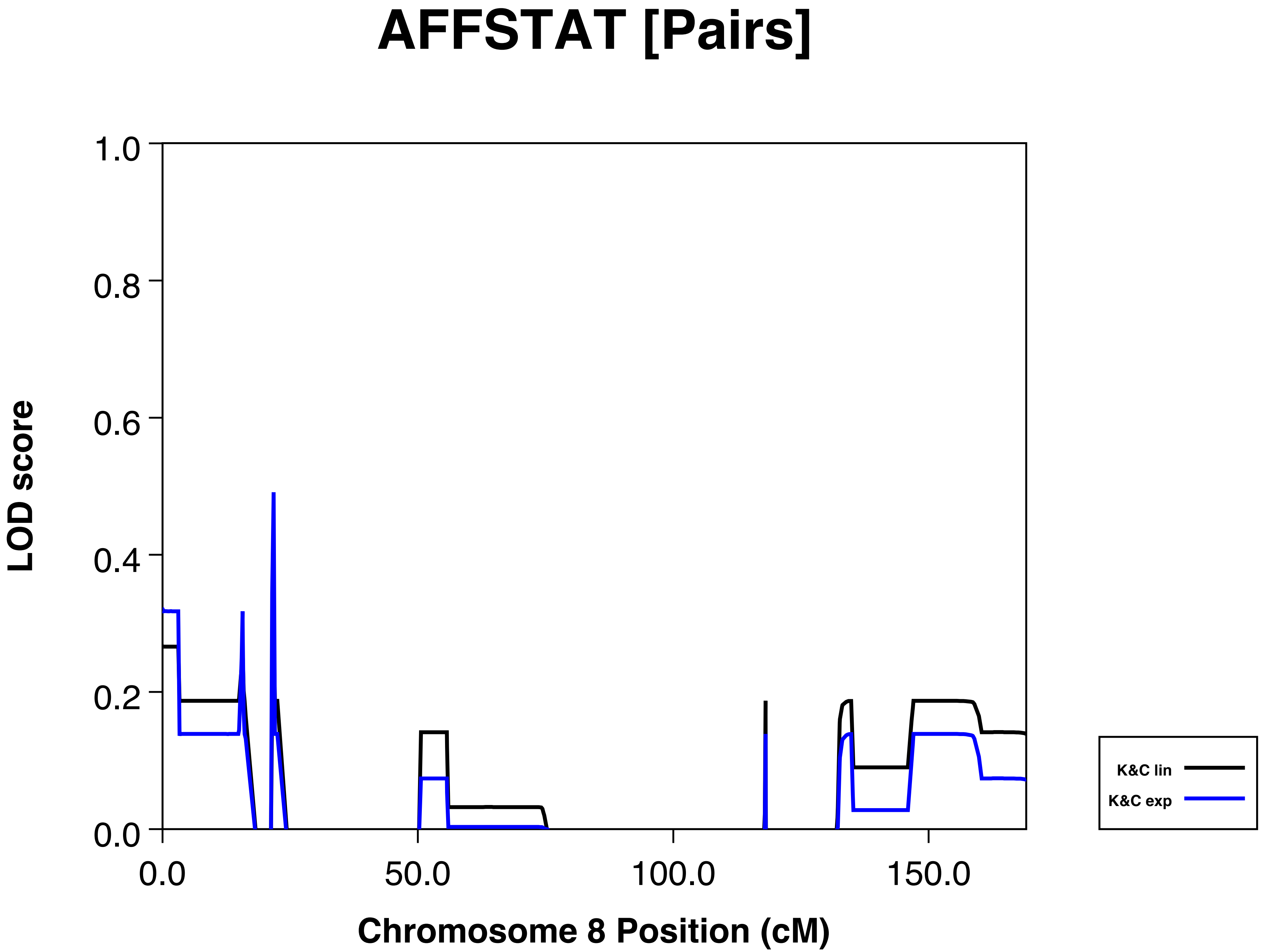
AFFSTAT [Pairs]
LOD score
Chromosome 8 Position (cM)
1.0
0.8
0.6
0.4
0.2
0.0
0.0
50.0
100.0
150.0
K&C lin
K&C exp

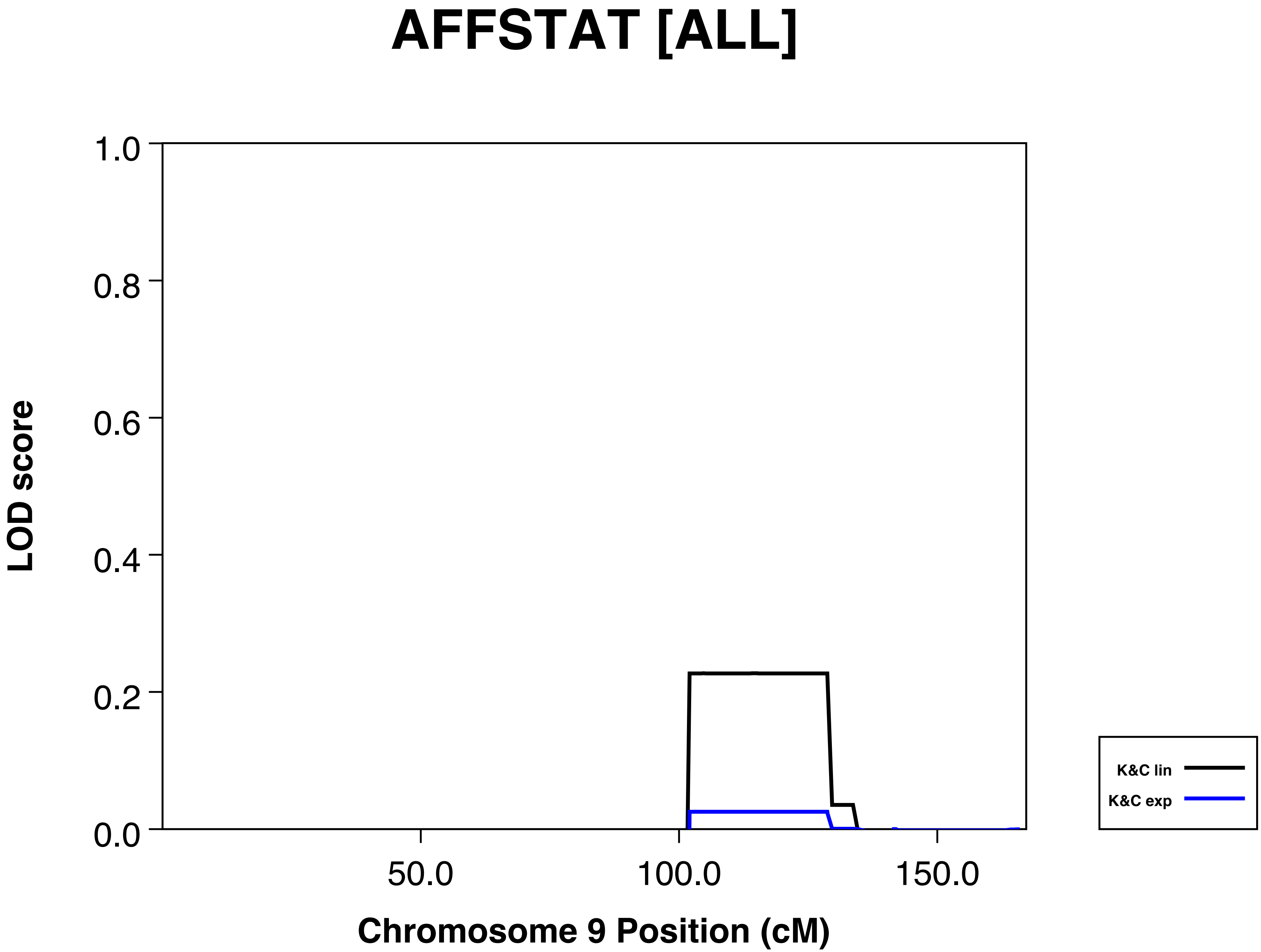
AFFSTAT [ALL]
LOD score
1.0
0.8
0.6
0.4
0.2
0.0
50.0
100.0
150.0
Chromosome 9 Position (cM)
K&C lin
K&C exp

AFFSTAT [Pairs]

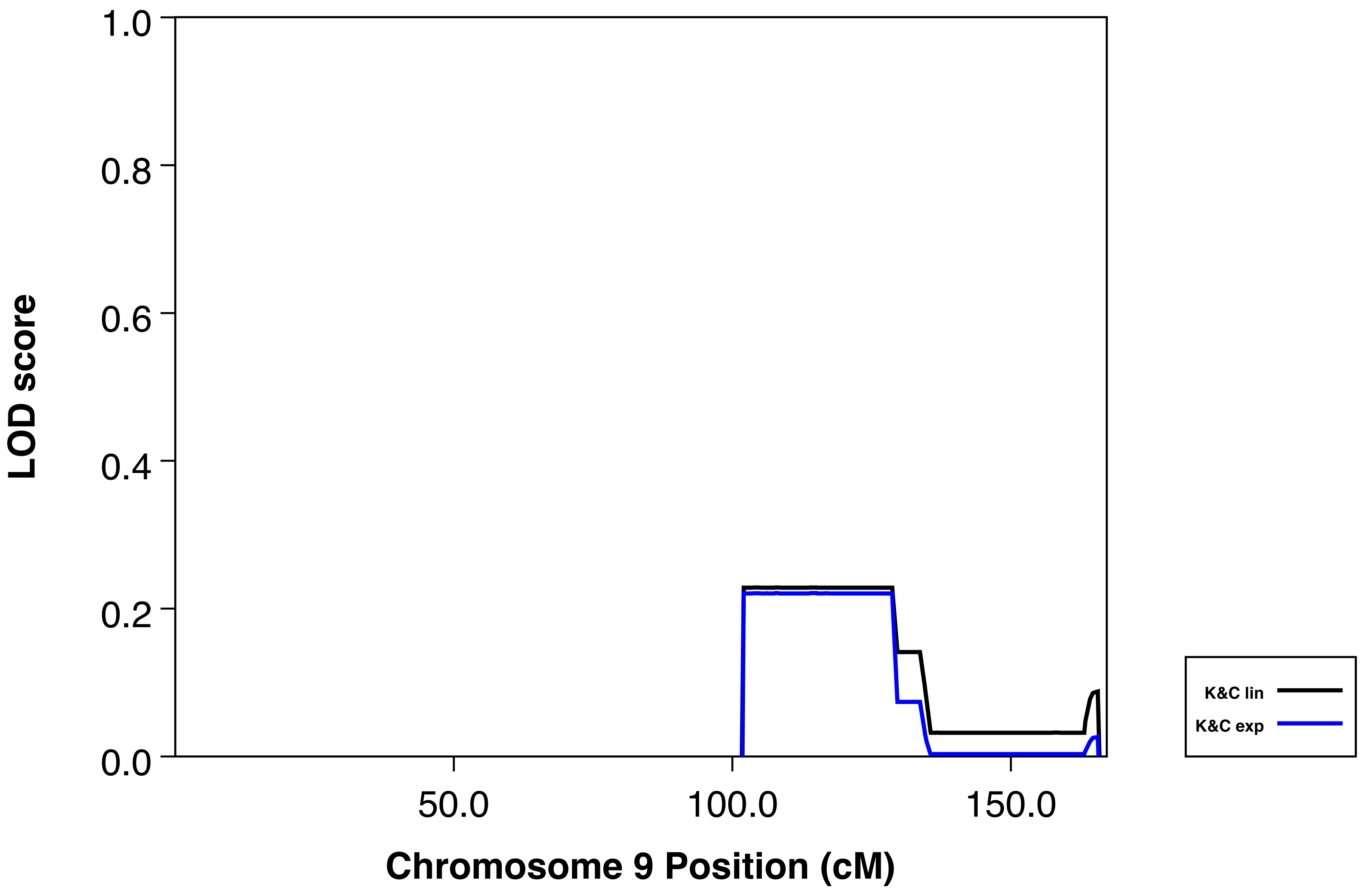

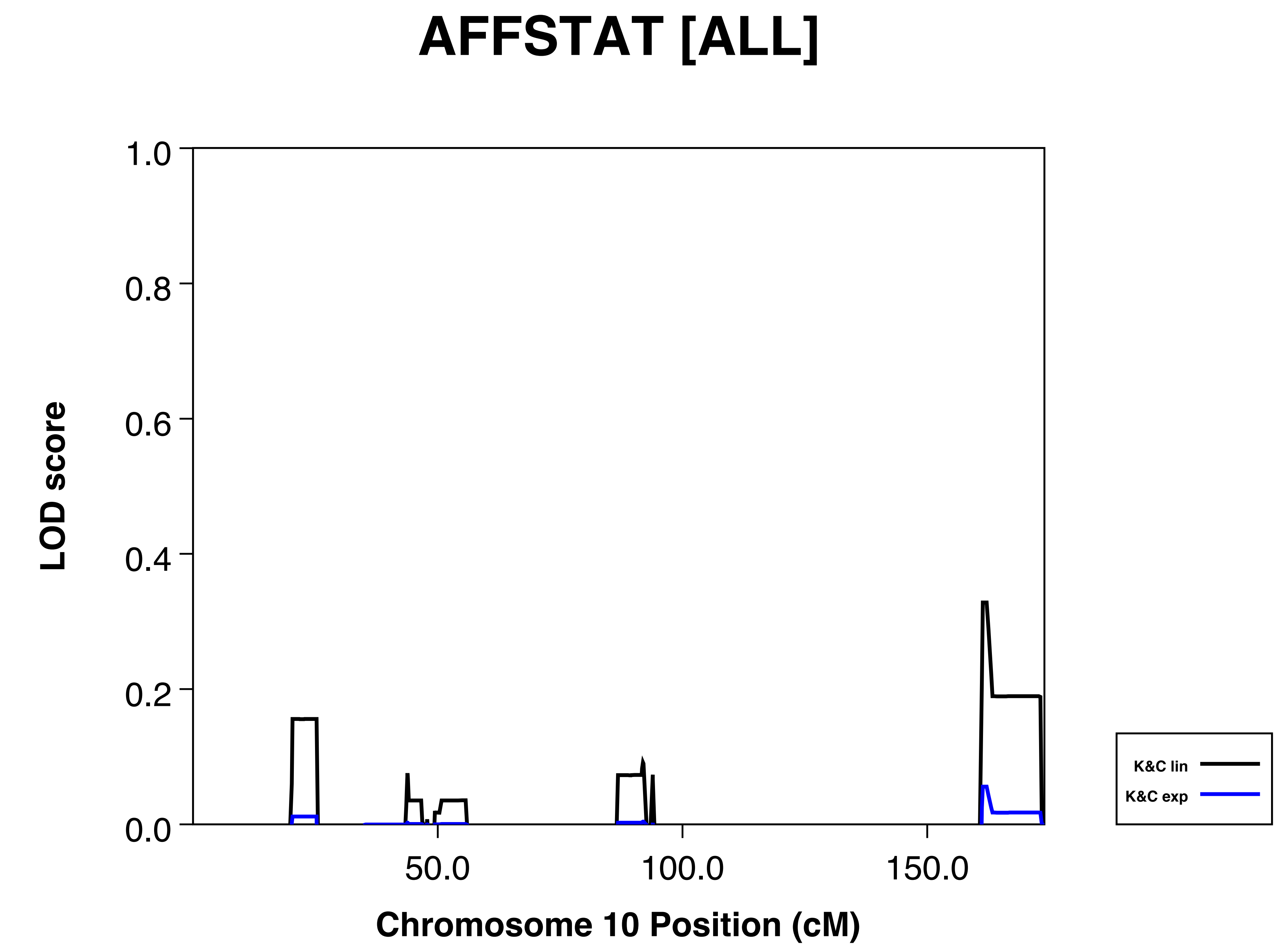
AFFSTAT [ALL]
LOD score
1.0
0.8
0.6
0.4
0.2
0.0
50.0
100.0
150.0
Chromosome 10 Position (cM)
K&C lin
K&C exp

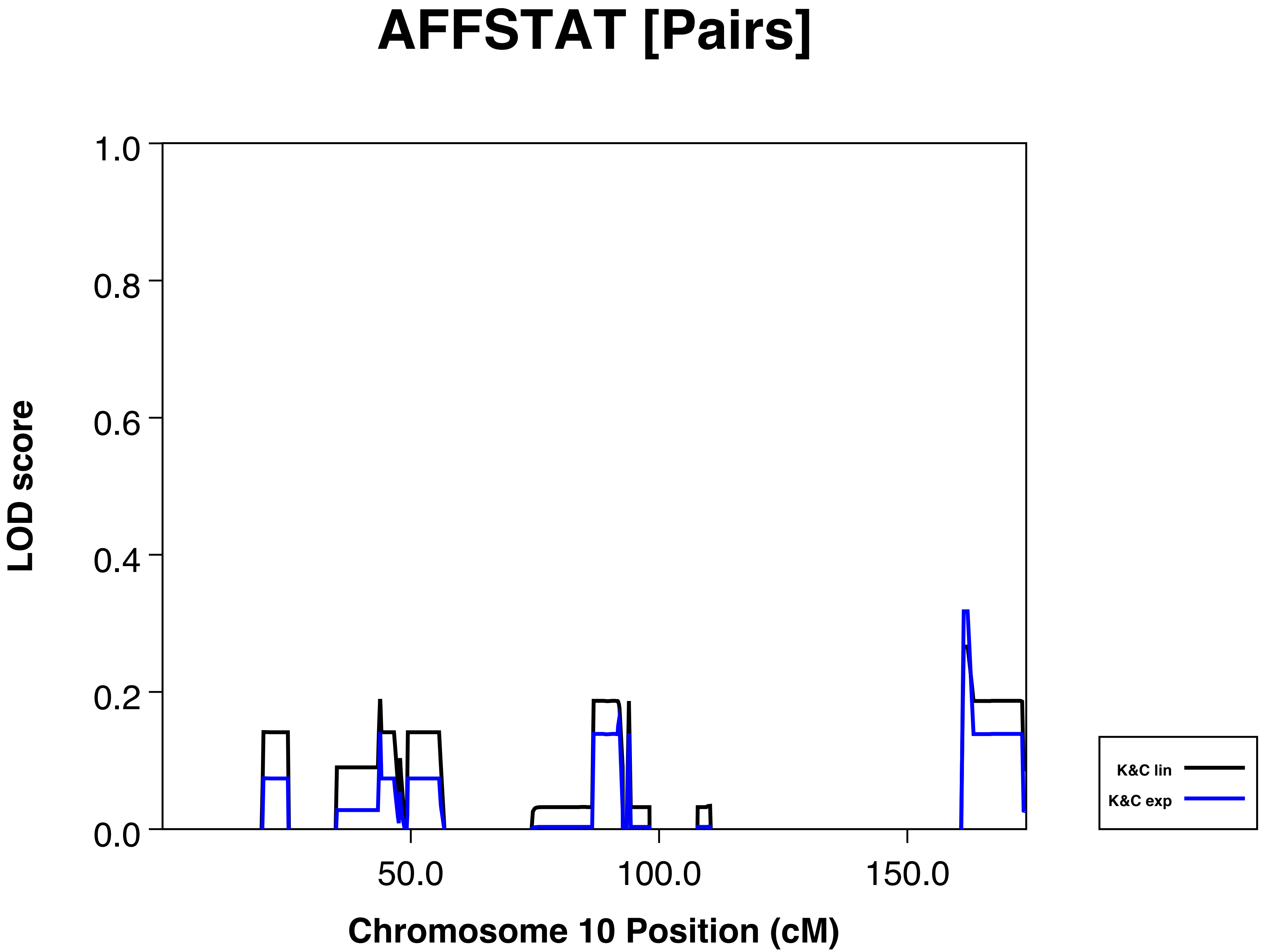

AFFSTAT [Pairs]
LOD score
1.0
0.8
0.6
0.4
0.2
0.0
50.0
100.0
150.0
Chromosome 10 Position (cM)
K&C lin
K&C exp

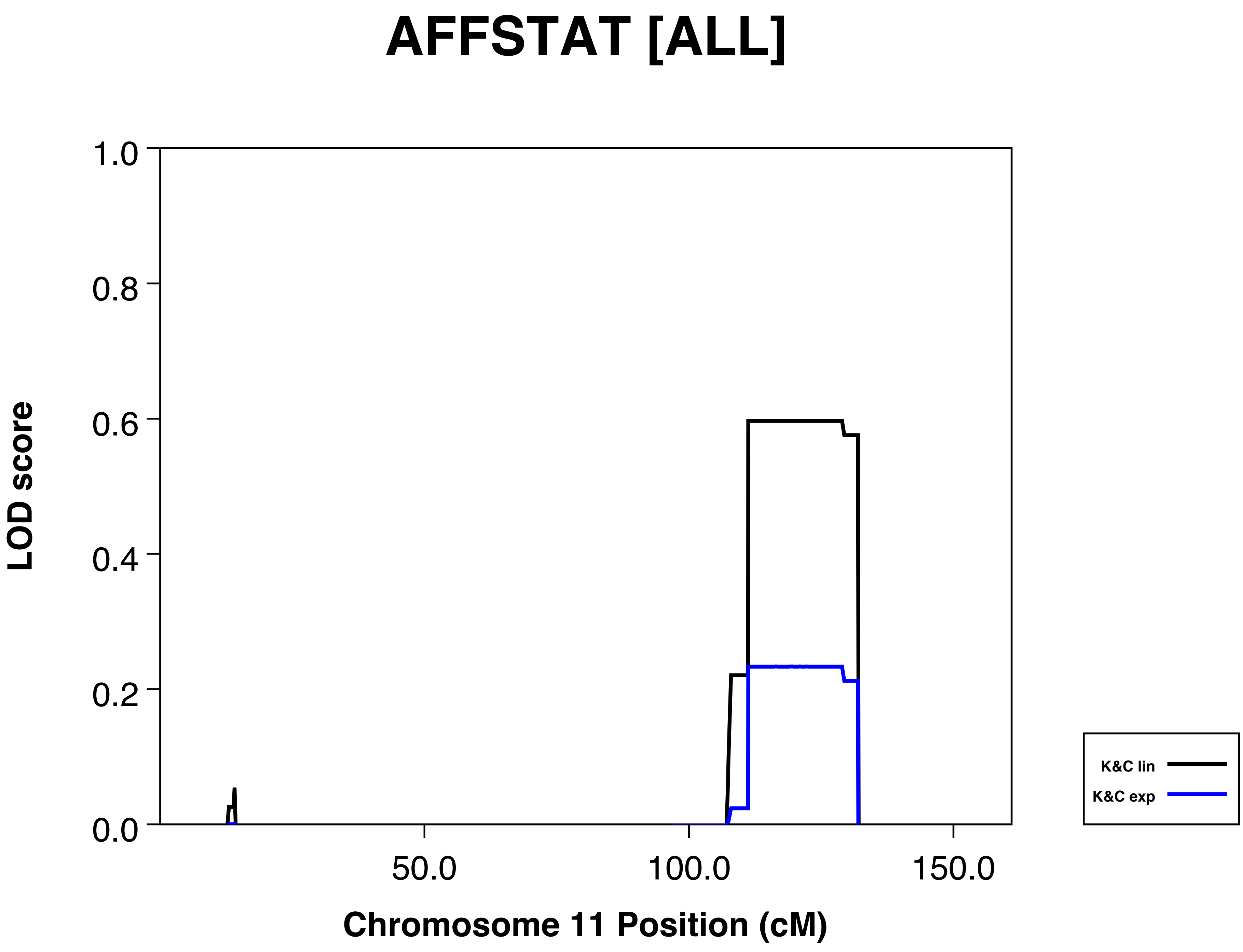

AFFSTAT [ALL]
LOD score
1.0
0.8
0.6
0.4
0.2
0.0
50.0
100.0
150.0
Chromosome 11 Position (cM)
K&C lin
K&C exp

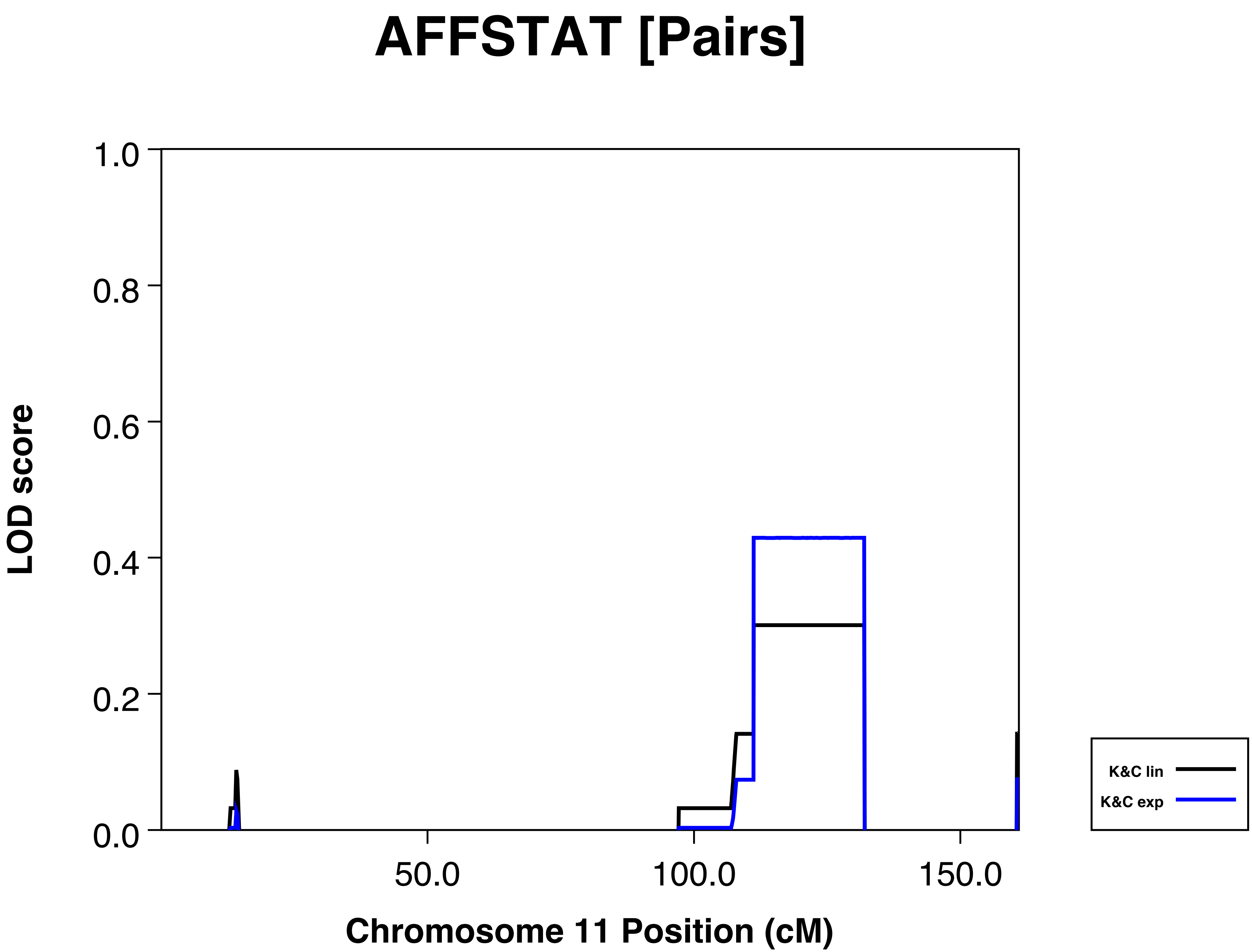
AFFSTAT [Pairs]
LOD score
1.0
0.8
0.6
0.4
0.2
0.0
50.0
100.0
150.0
Chromosome 11 Position (cM)
K&C lin
K&C exp

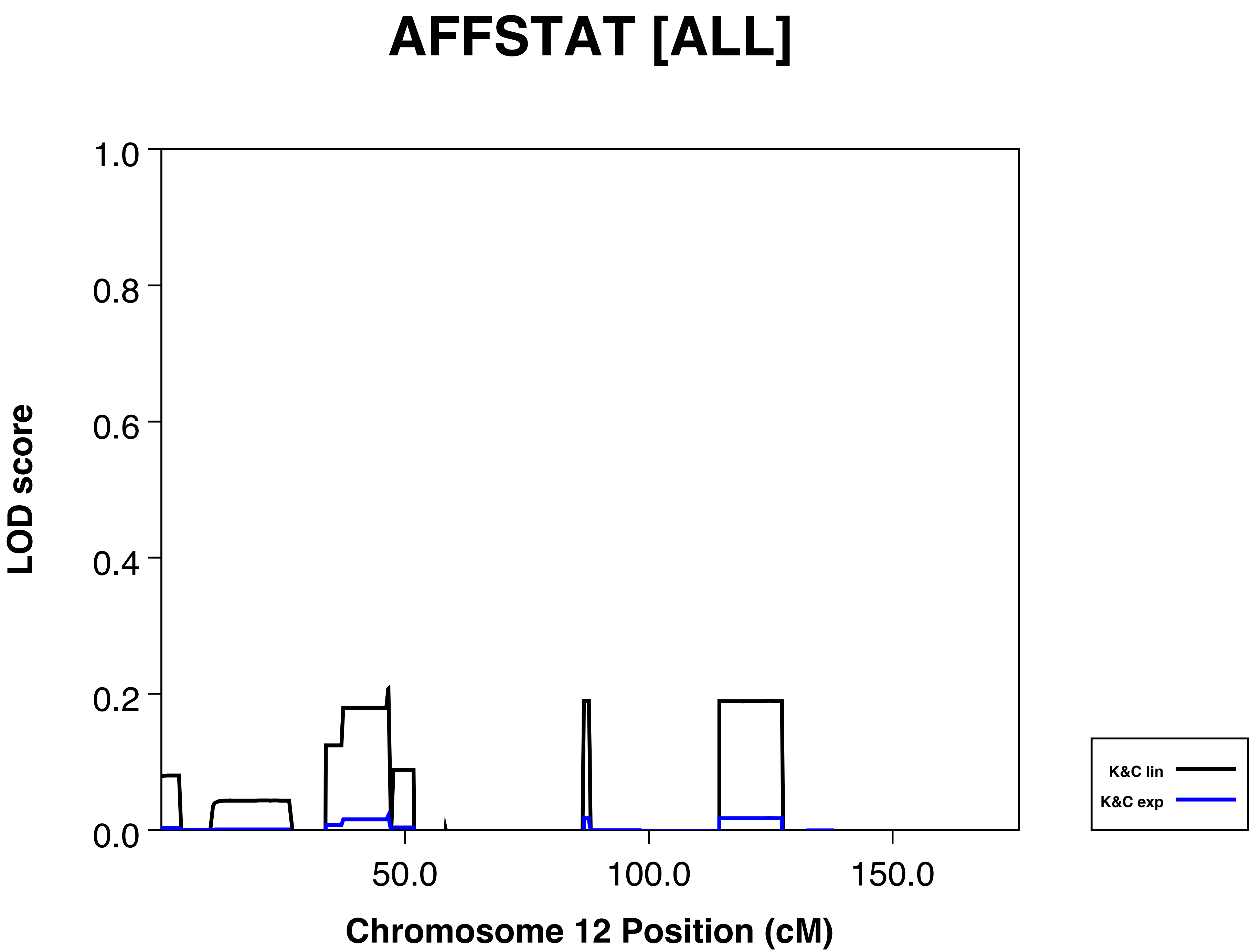
AFFSTAT [ALL]
1.0
0.8
0.6
0.4
0.2
0.0
LOD score
50.0
100.0
150.0
Chromosome 12 Position (cM)
K&C lin
K&C exp

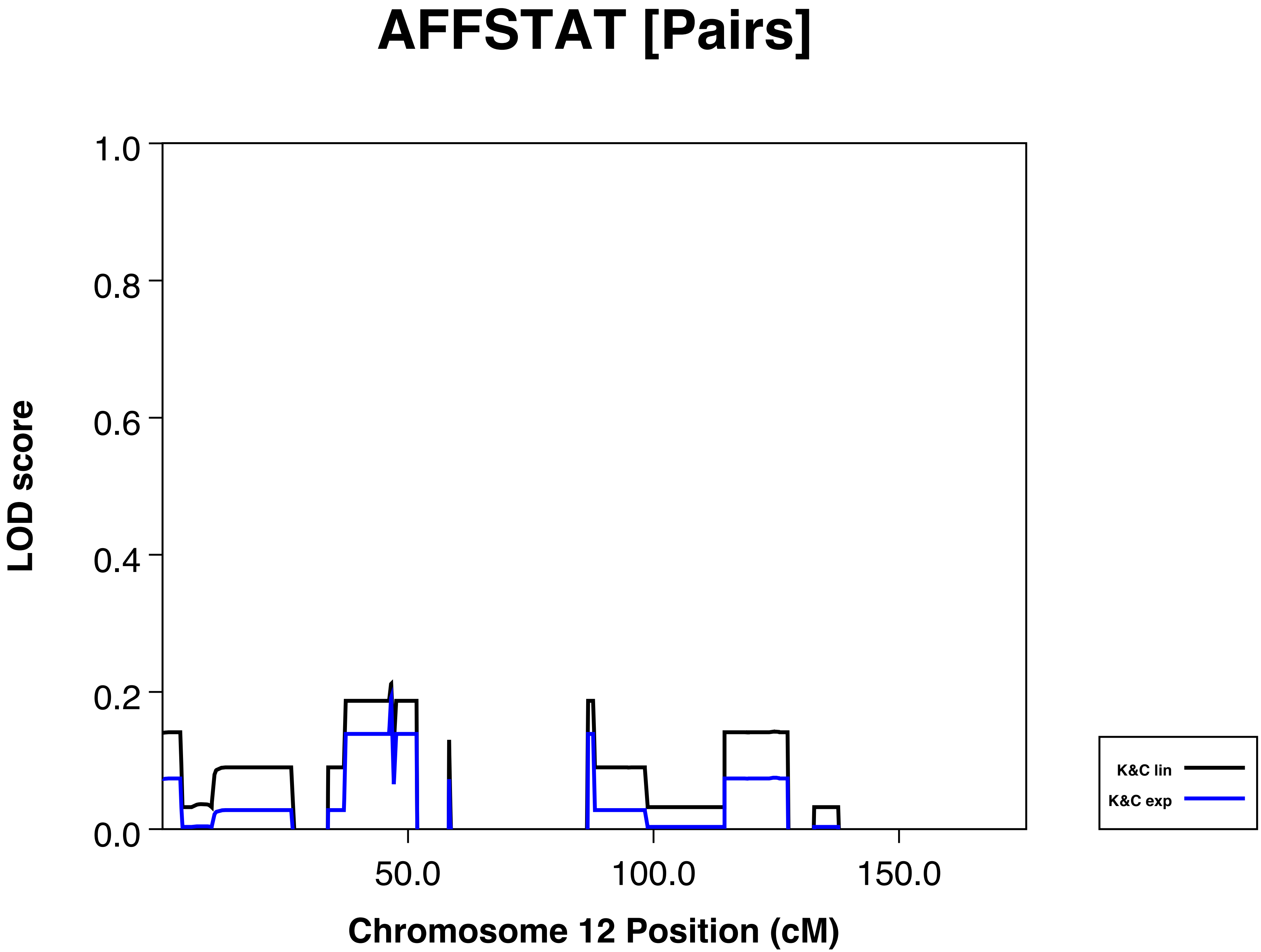
AFFSTAT [Pairs]
LOD score
1.0
0.8
0.6
0.4
0.2
0.0
50.0
100.0
150.0
Chromosome 12 Position (cM)
K&C lin
K&C exp

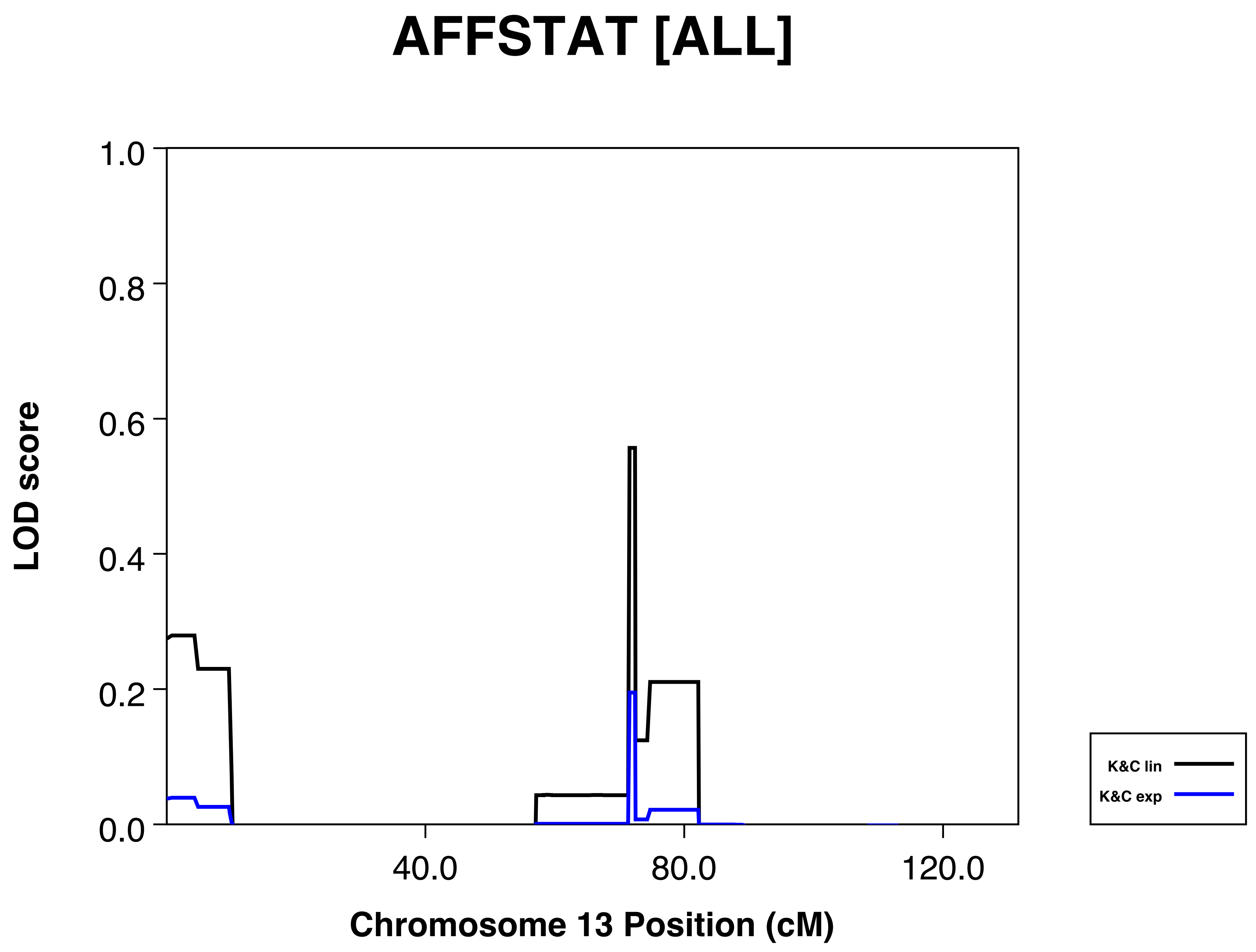
AFFSTAT [ALL]
LOD score
1.0
0.8
0.6
0.4
0.2
0.0
40.0
80.0
120.0
Chromosome 13 Position (cM)
K&C lin
K&C exp

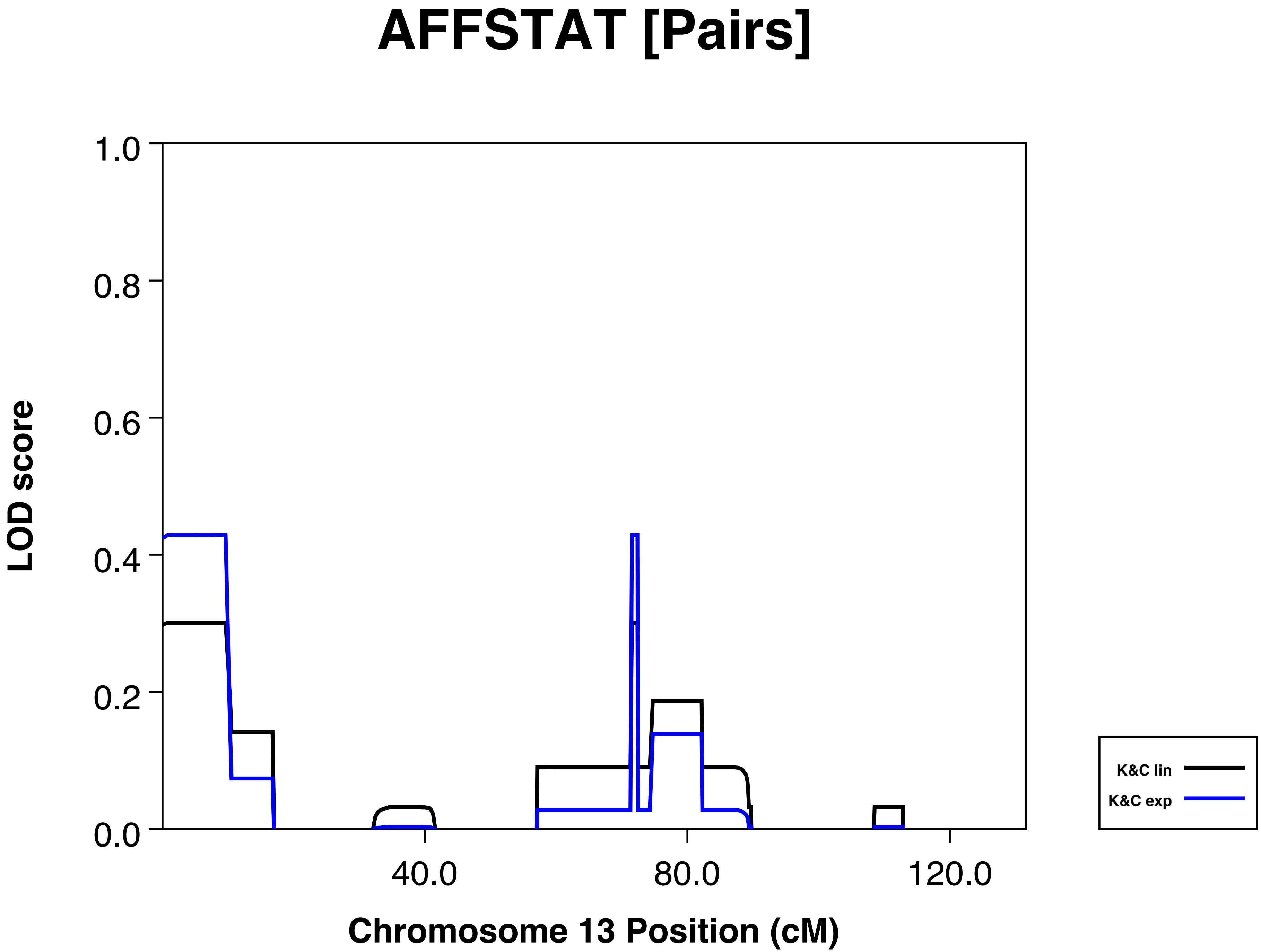

AFFSTAT [Pairs]
LOD score
1.0
0.8
0.6
0.4
0.2
0.0
40.0
80.0
120.0
Chromosome 13 Position (cM)
K&C lin
K&C exp

AFFSTAT [ALL]

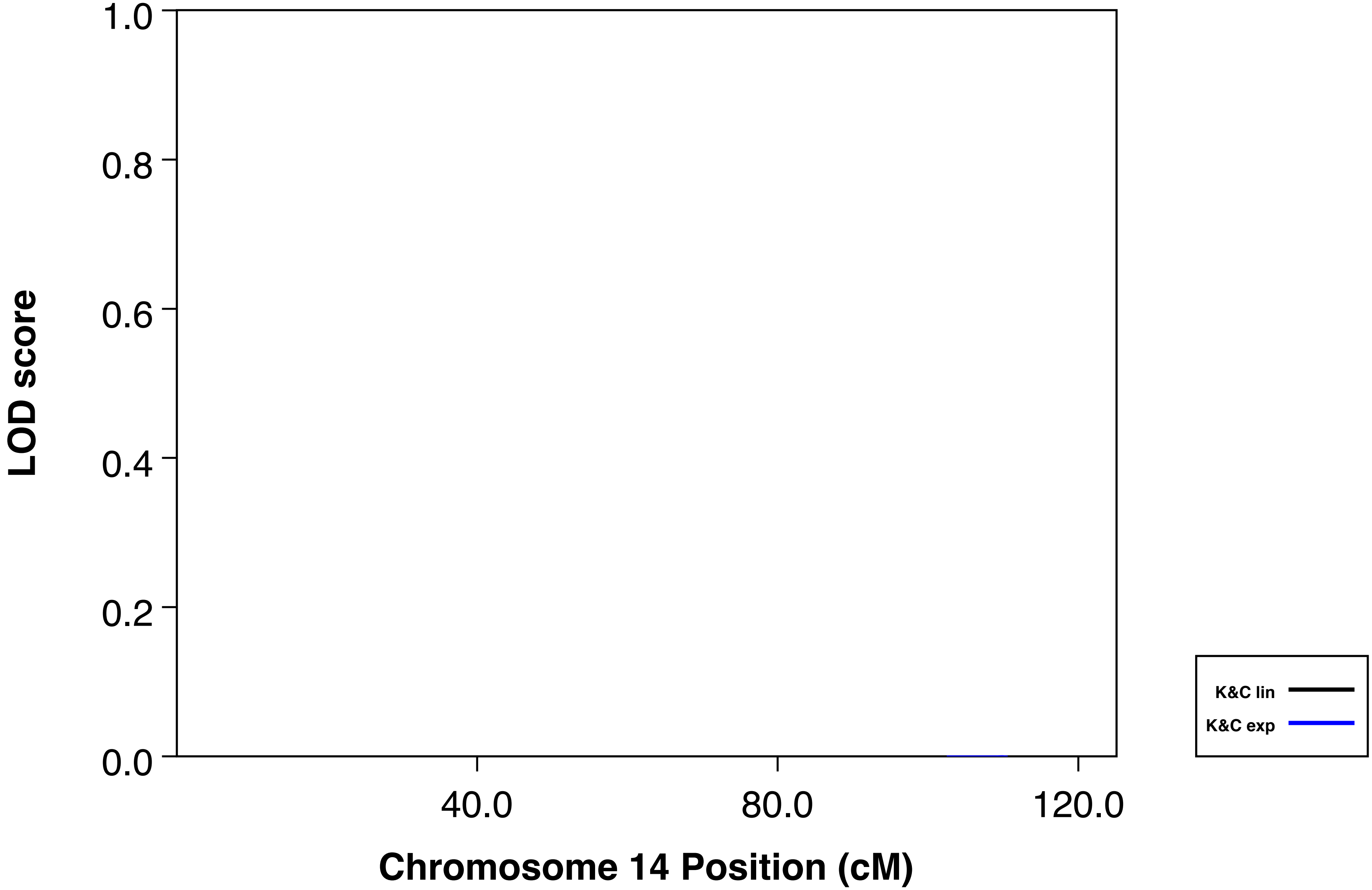
1.0
0.8
0.6
0.4
0.2
0.0
LOD score
40.0
80.0
120.0
Chromosome 14 Position (cM)
K&C lin
K&C exp

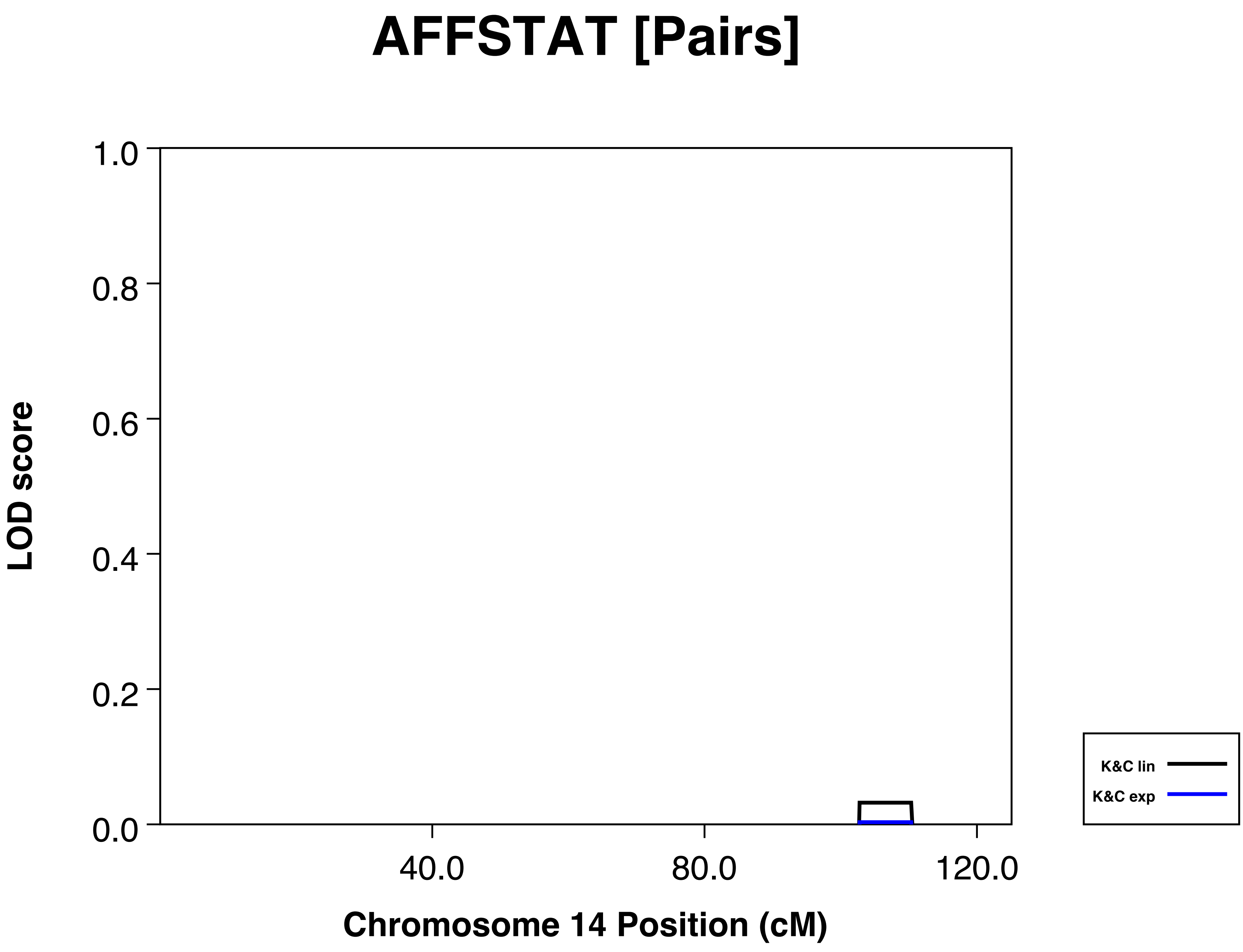
AFFSTAT [Pairs]
LOD score
1.0
0.8
0.6
0.4
0.2
0.0
40.0
80.0
120.0
Chromosome 14 Position (cM)
K&C lin
K&C exp

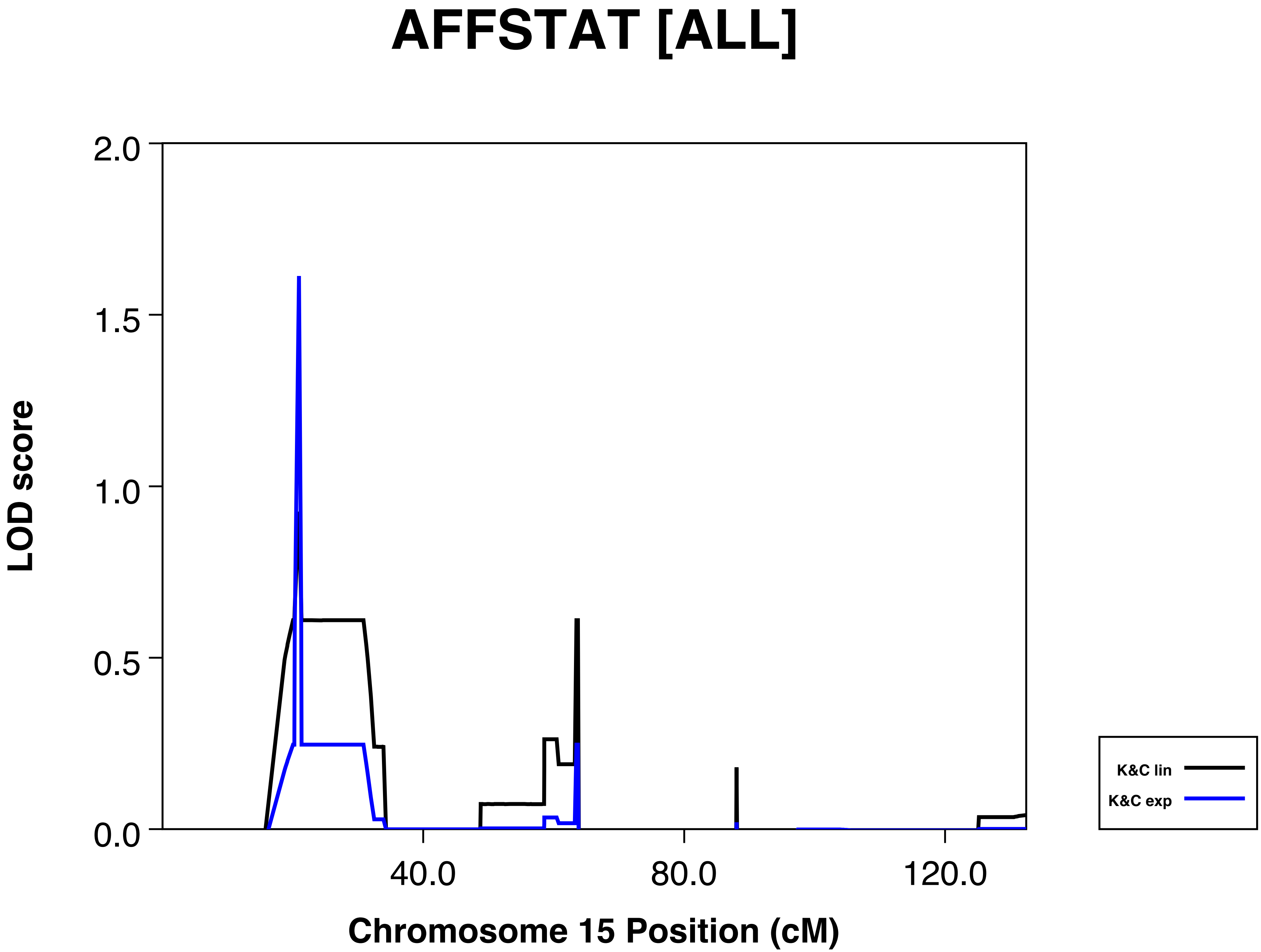
AFFSTAT [ALL]
LOD score
2.0
1.5
1.0
0.5
0.0
40.0
80.0
120.0
Chromosome 15 Position (cM)
K&C lin
K&C exp

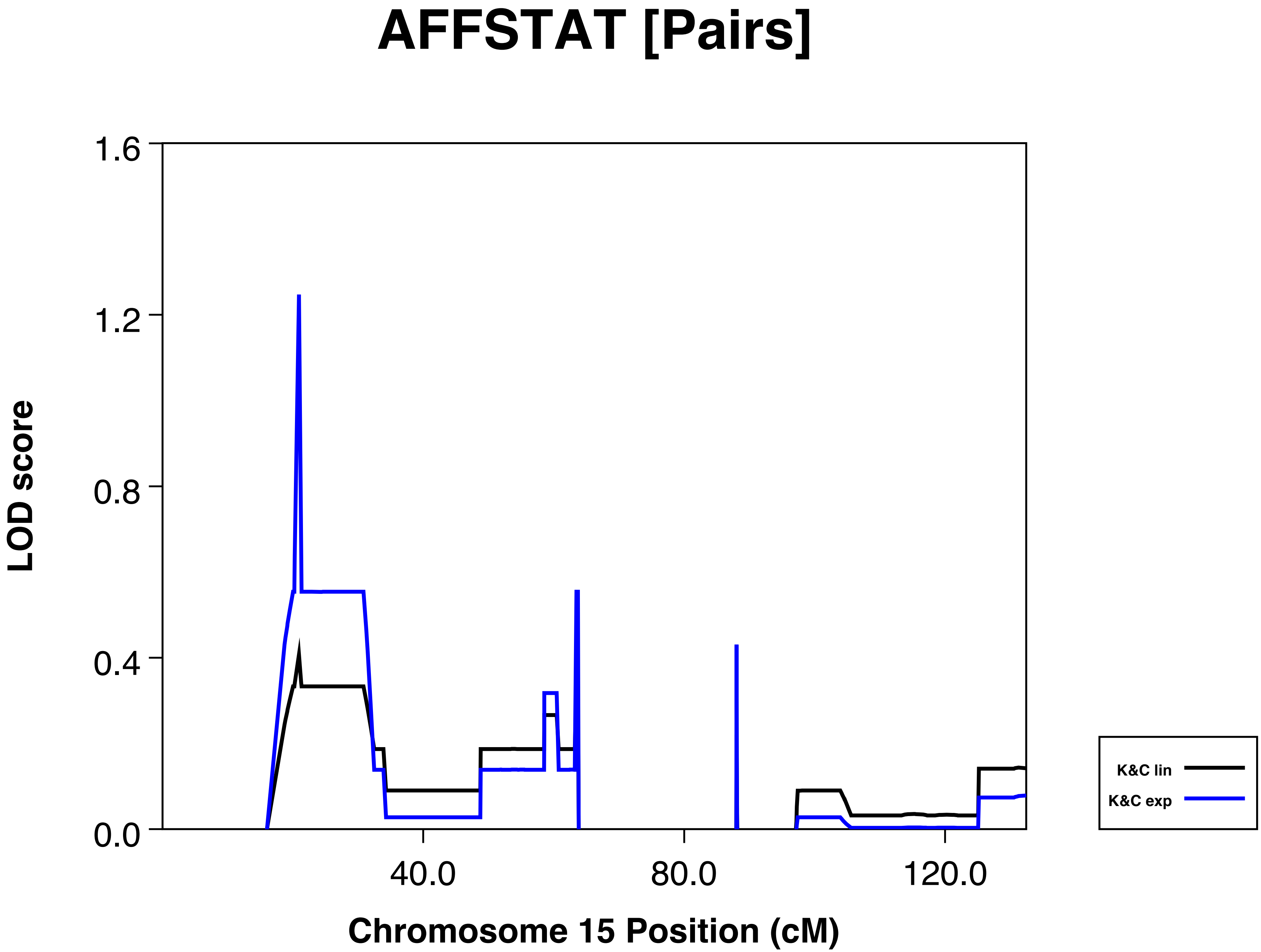

AFFSTAT [Pairs]
LOD score
0.0
0.4
0.8
1.2
1.6
40.0
80.0
120.0
Chromosome 15 Position (cM)
K&C lin
K&C exp

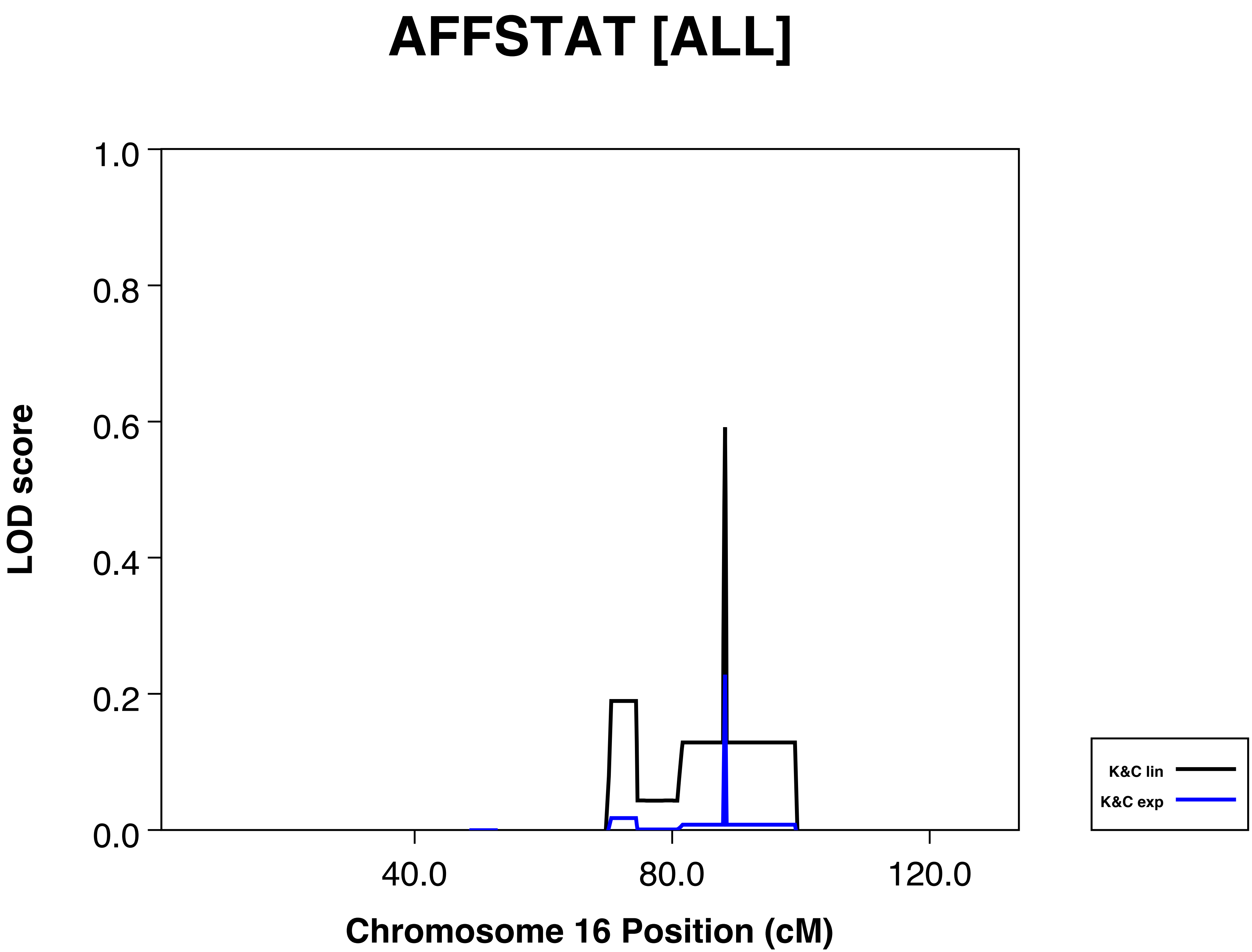

AFFSTAT [ALL]
LOD score
1.0
0.8
0.6
0.4
0.2
0.0
40.0
80.0
120.0
Chromosome 16 Position (cM)
K&C lin
K&C exp

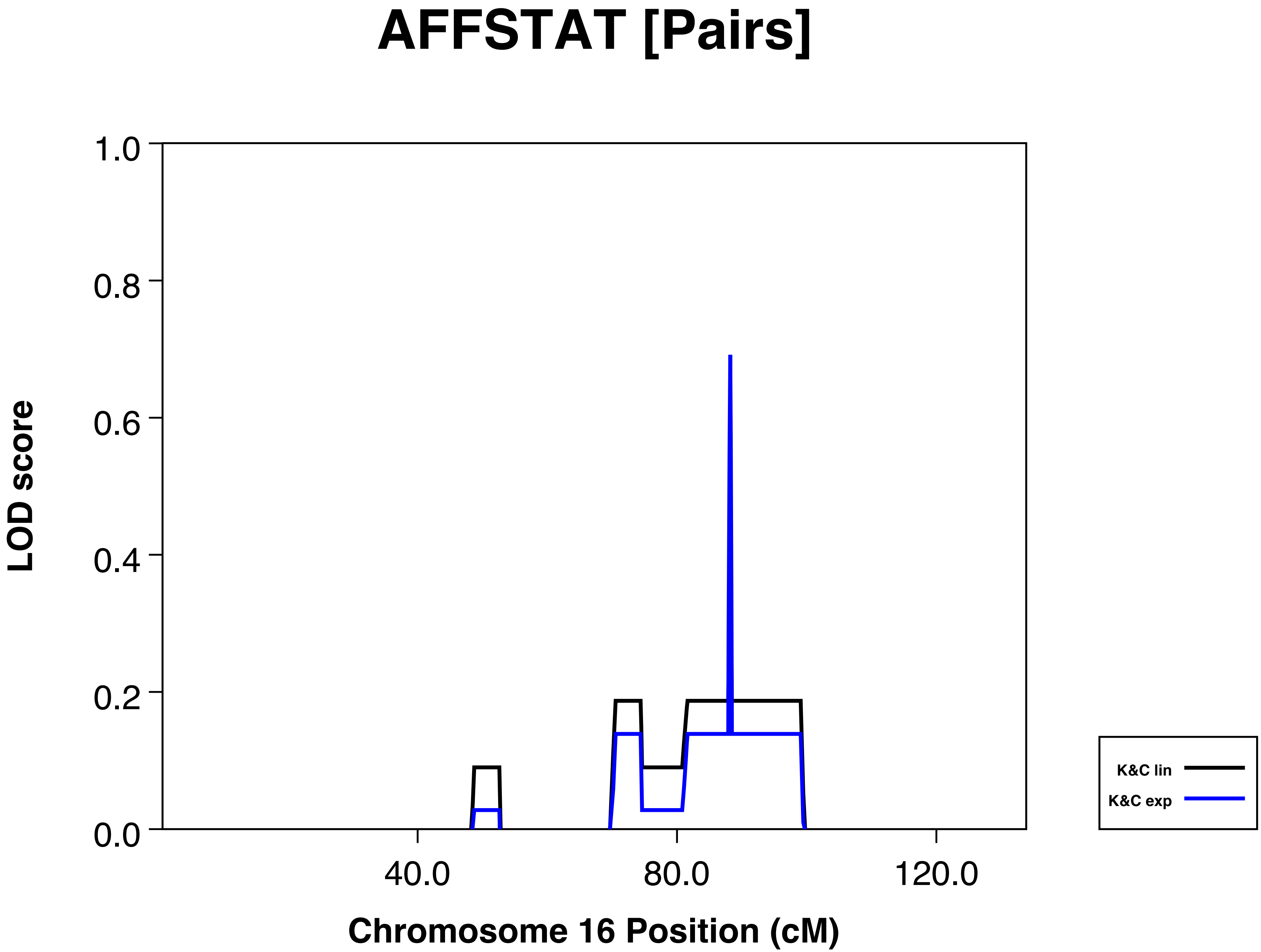
AFFSTAT [Pairs]
LOD score
1.0
0.8
0.6
0.4
0.2
0.0
40.0
80.0
120.0
Chromosome 16 Position (cM)
K&C lin
K&C exp

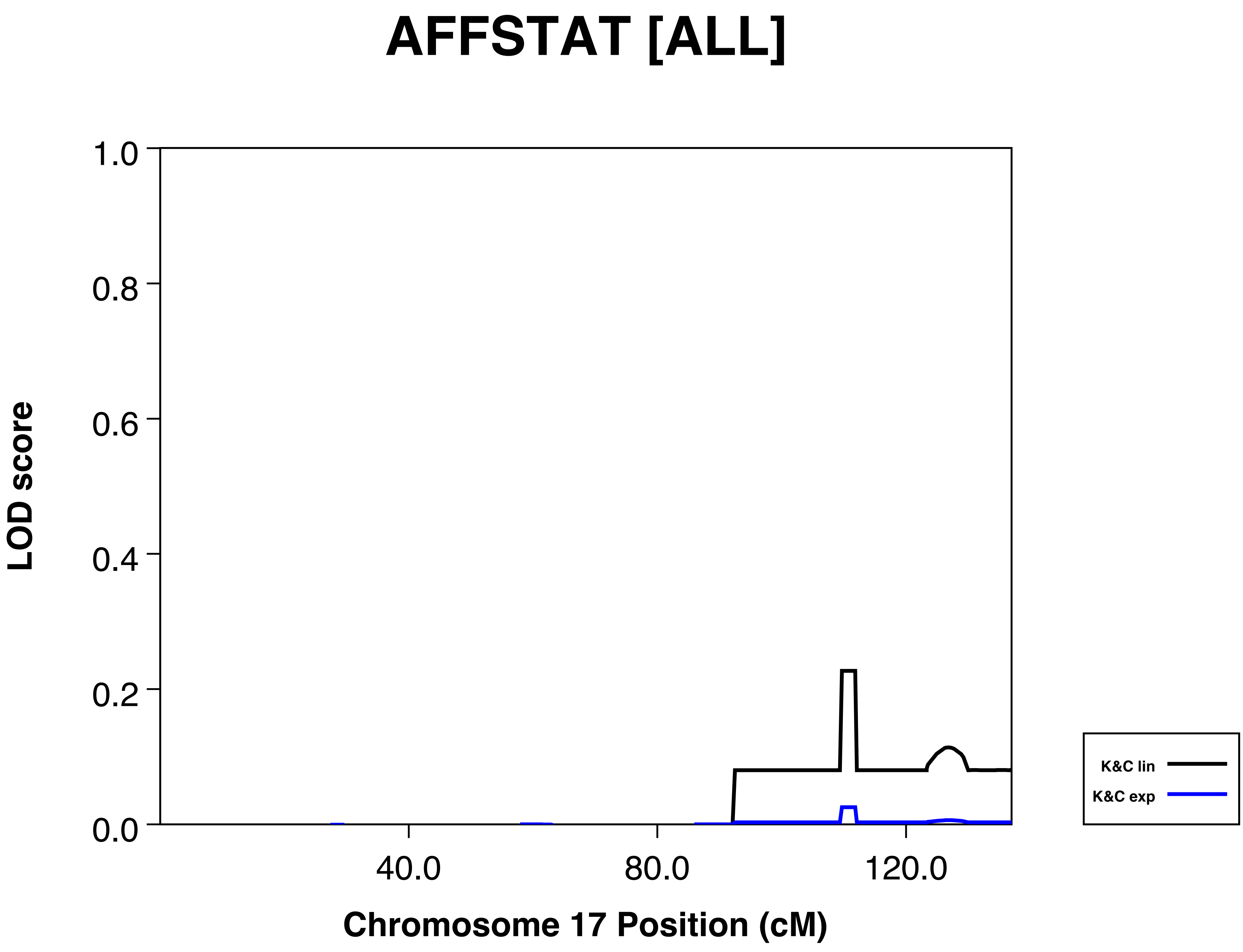
AFFSTAT [ALL]
LOD score
1.0
0.8
0.6
0.4
0.2
0.0
40.0
80.0
120.0
Chromosome 17 Position (cM)
K&C lin
K&C exp

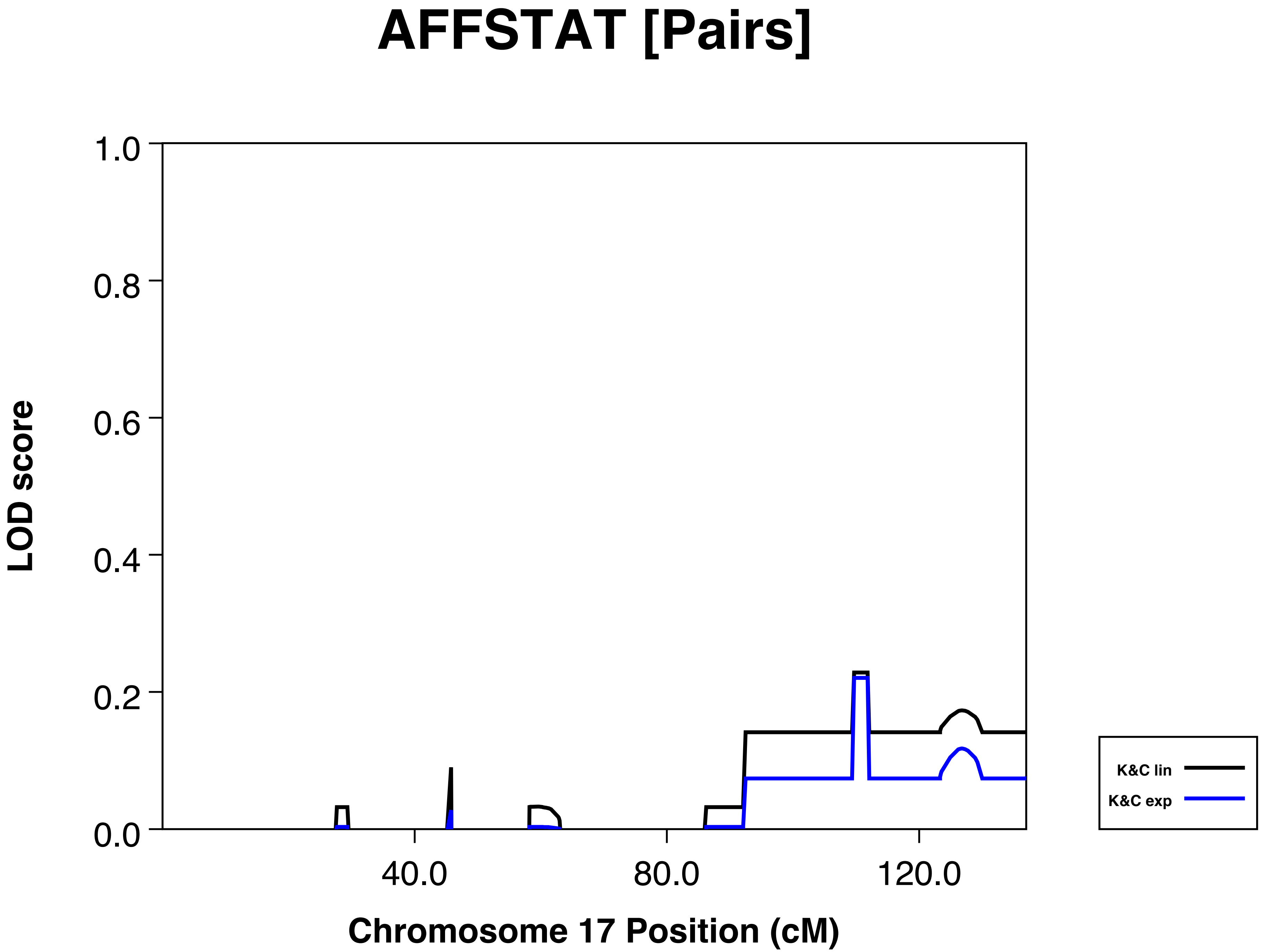

AFFSTAT [Pairs]
LOD score
1.0
0.8
0.6
0.4
0.2
0.0
40.0
80.0
120.0
Chromosome 17 Position (cM)
K&C lin
K&C exp

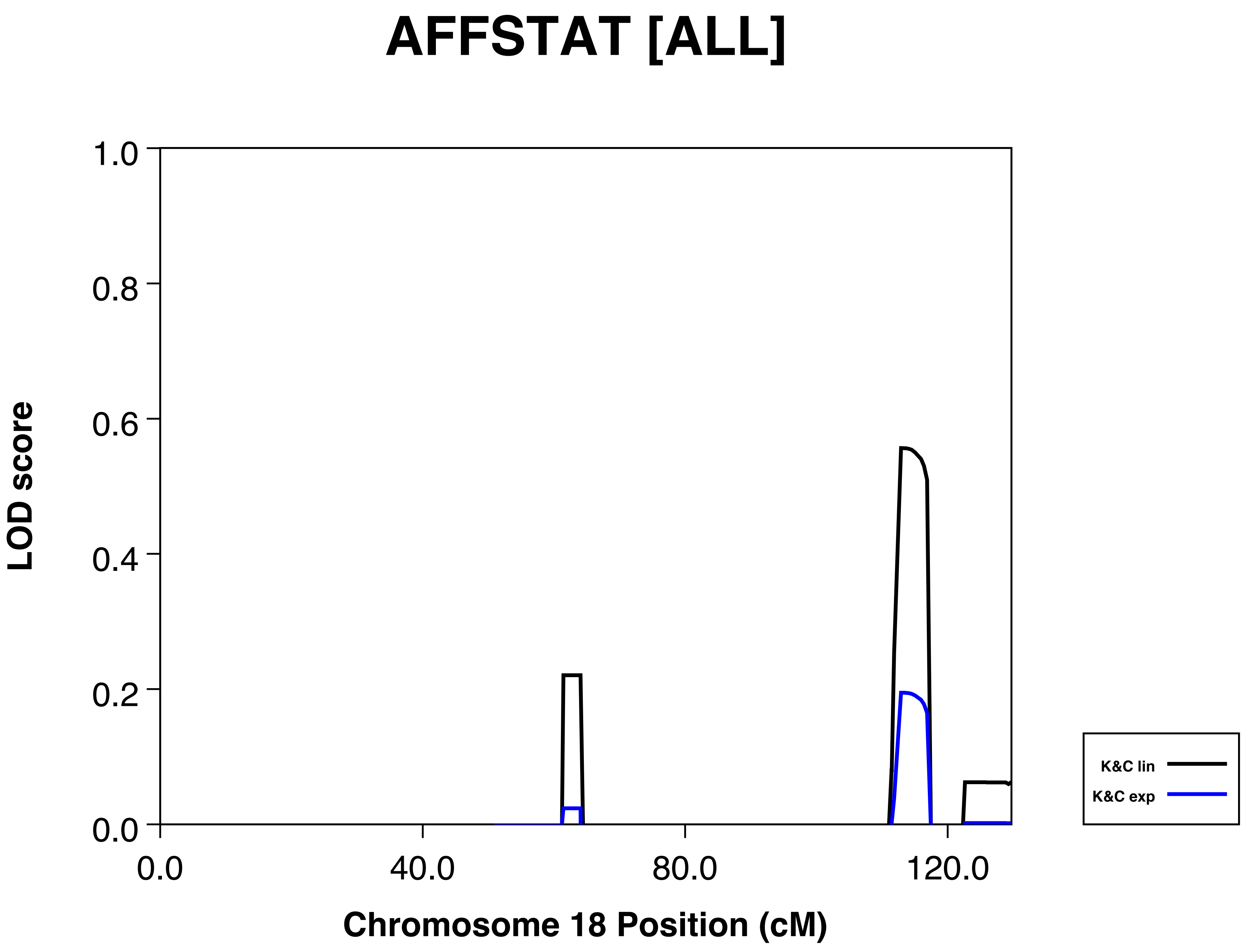

AFFSTAT [ALL]
1.0
0.8
0.6
0.4
0.2
0.0
LOD score
0.0
40.0
80.0
120.0
Chromosome 18 Position (cM)
K&C lin
K&C exp

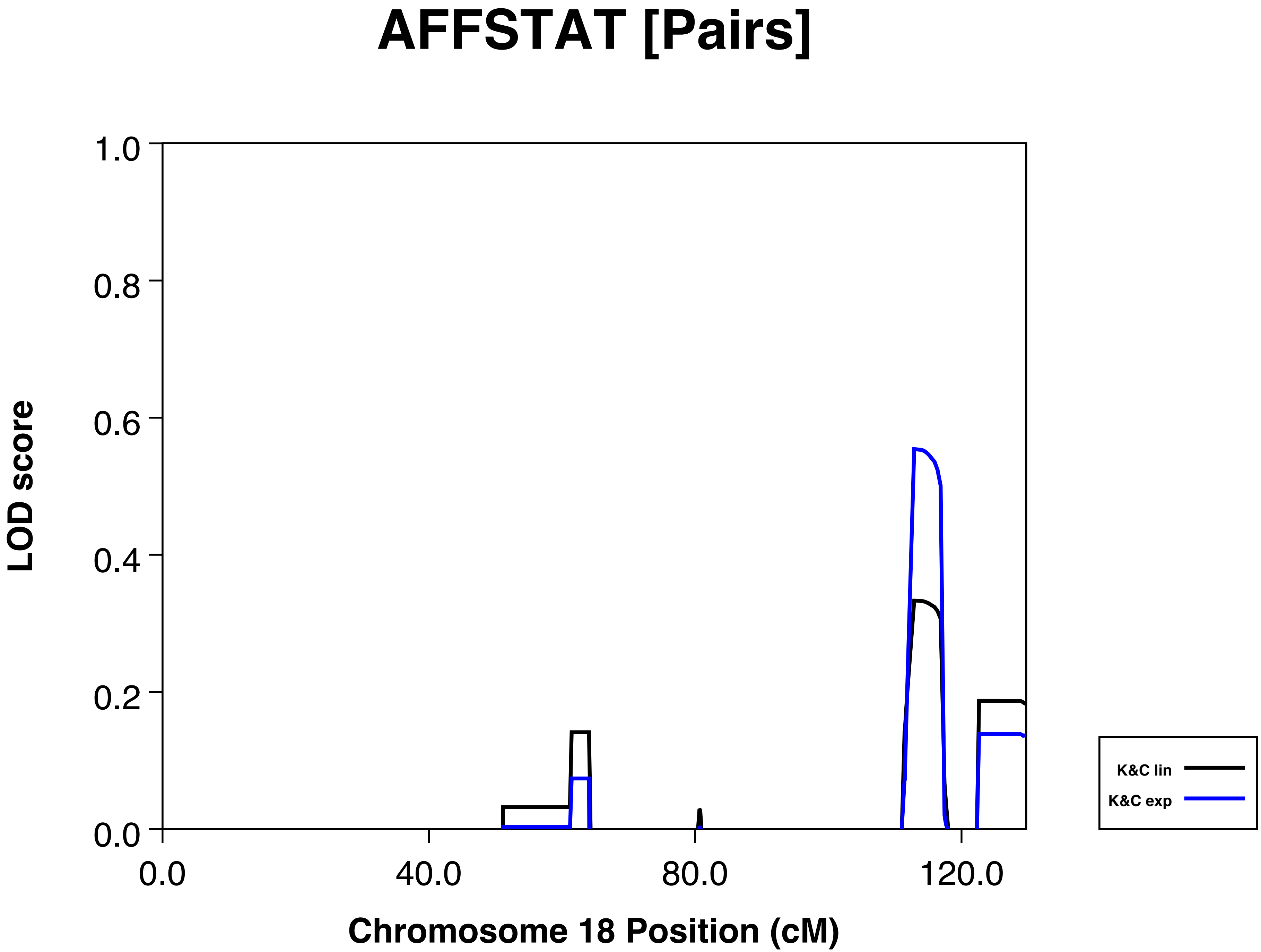

AFFSTAT [Pairs]
LOD score
1.0
0.8
0.6
0.4
0.2
0.0
0.0
40.0
80.0
120.0
Chromosome 18 Position (cM)
K&C lin
K&C exp

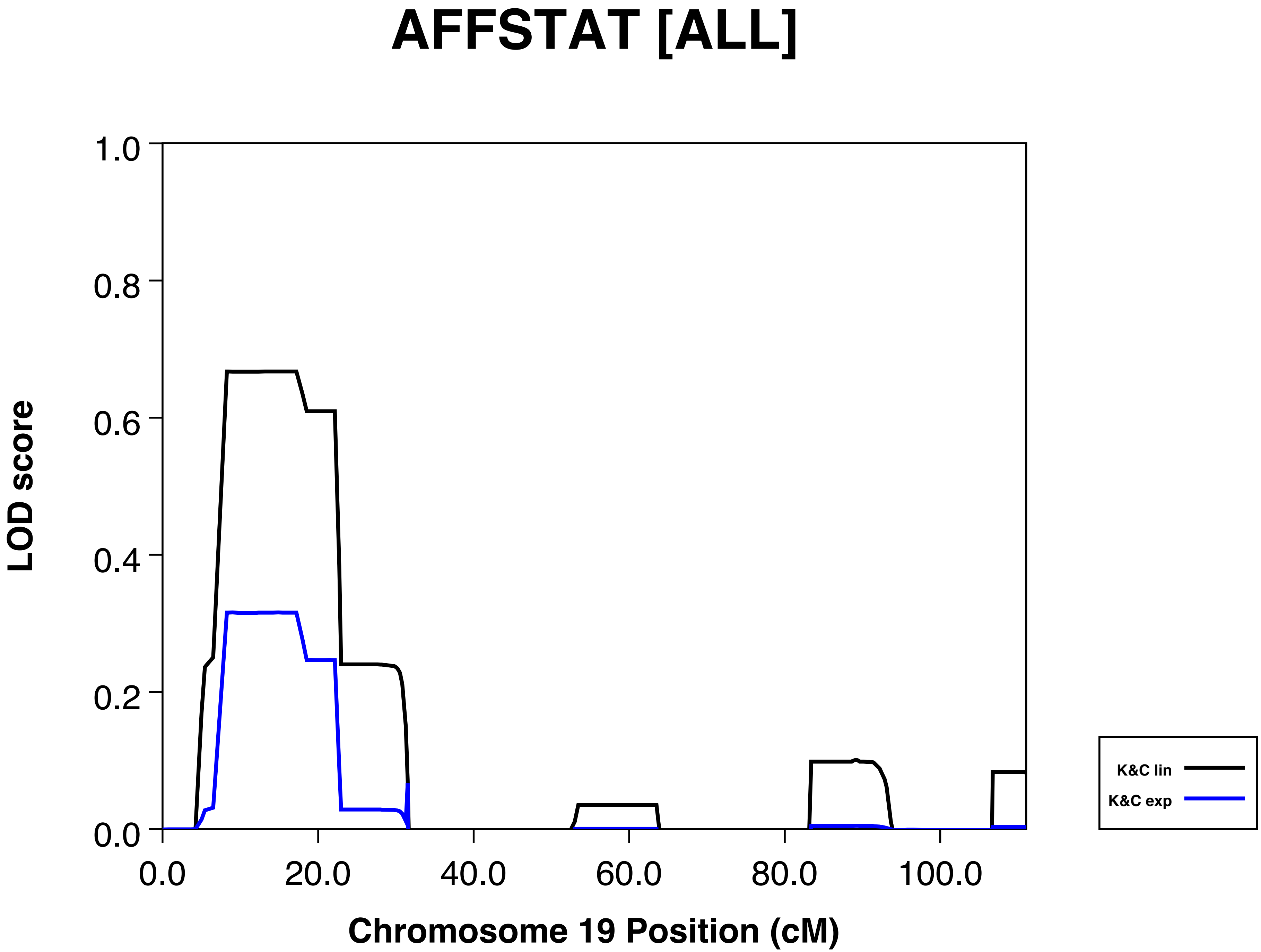
AFFSTAT [ALL]
LOD score
1.0
0.8
0.6
0.4
0.2
0.0
0.0
20.0
40.0
60.0
80.0
100.0
Chromosome 19 Position (cM)
K&C lin
K&C exp

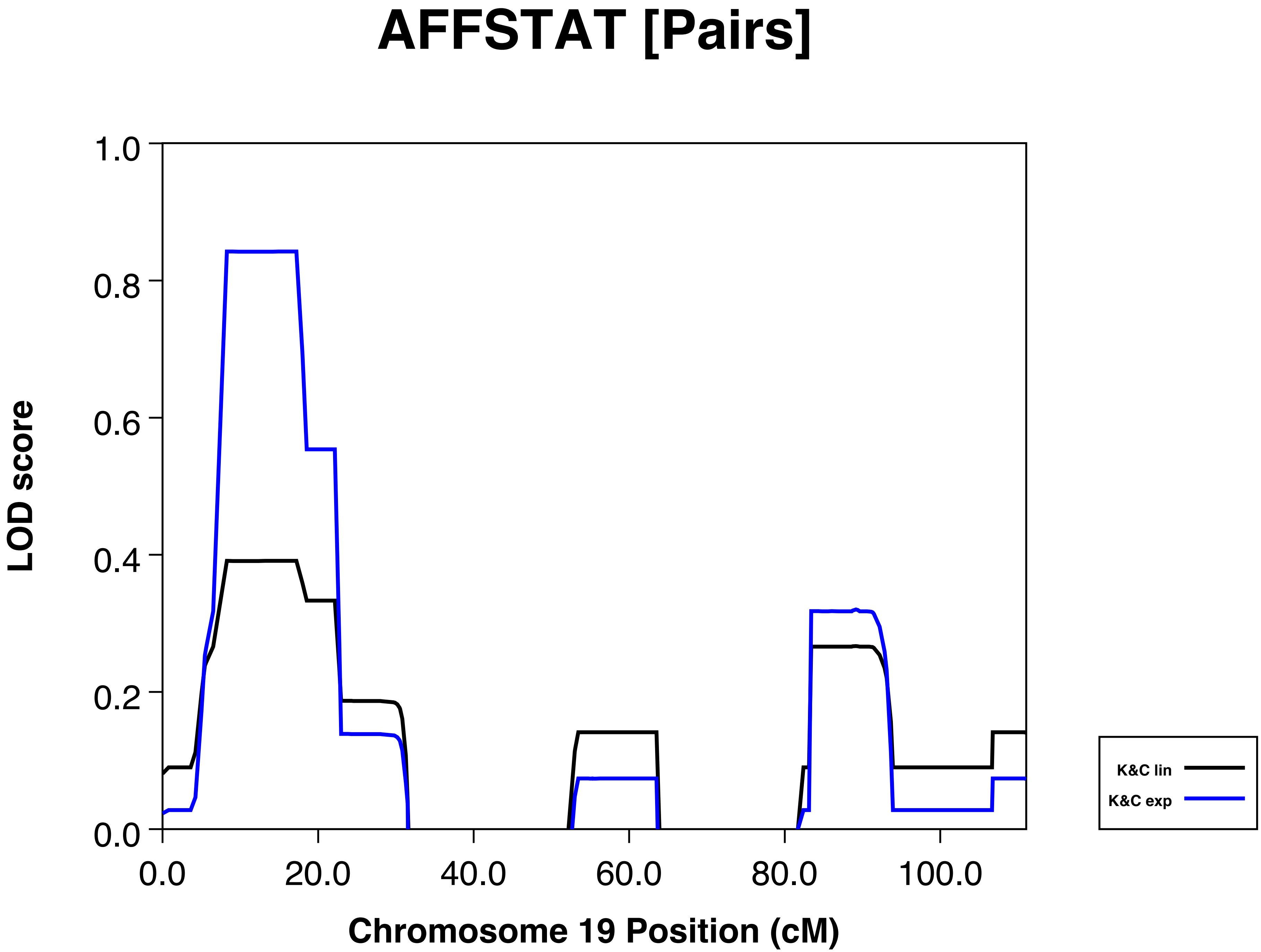
AFFSTAT [Pairs]
LOD score
1.0
0.8
0.6
0.4
0.2
0.0
0.0
20.0
40.0
60.0
80.0
100.0
Chromosome 19 Position (cM)
K&C lin
K&C exp

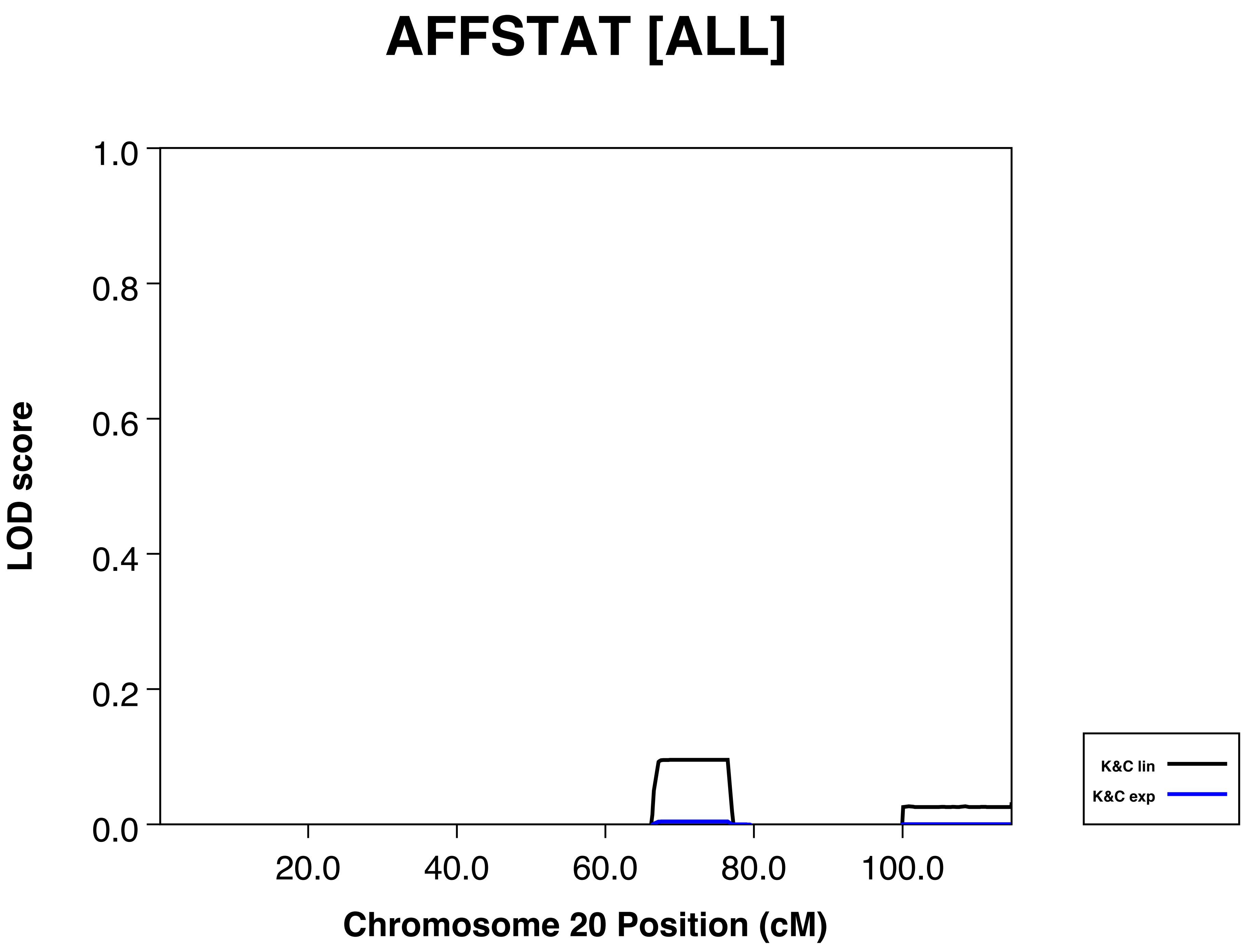
AFFSTAT [ALL]
1.0
0.8
0.6
0.4
0.2
0.0
LOD score
20.0
40.0
60.0
80.0
100.0
Chromosome 20 Position (cM)
K&C lin
K&C exp

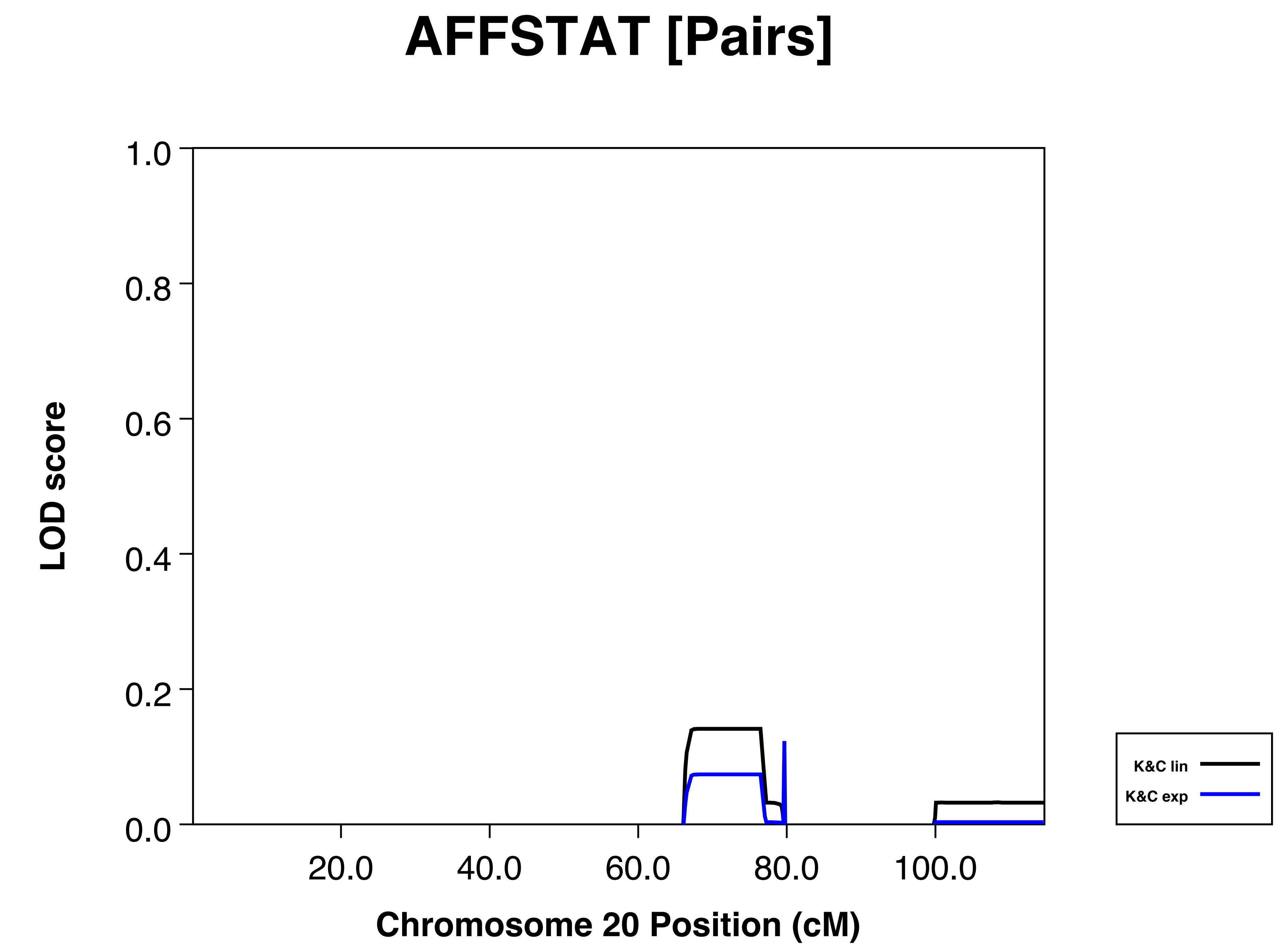

AFFSTAT [Pairs]
LOD score
1.0
0.8
0.6
0.4
0.2
0.0
20.0
40.0
60.0
80.0
100.0
Chromosome 20 Position (cM)
K&C lin
K&C exp

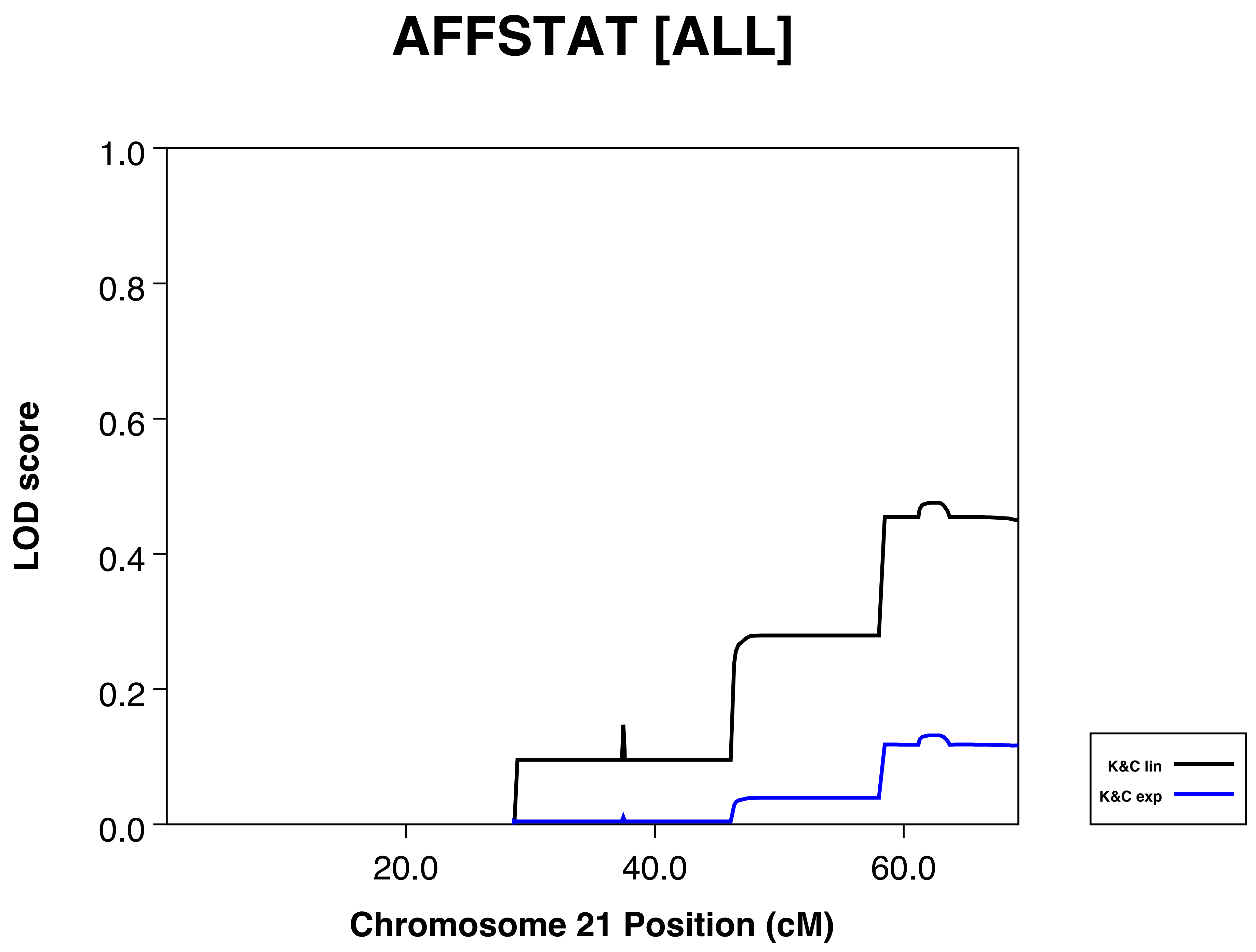

AFFSTAT [ALL]
LOD score
1.0
0.8
0.6
0.4
0.2
0.0
20.0
40.0
60.0
Chromosome 21 Position (cM)
K&C lin
K&C exp

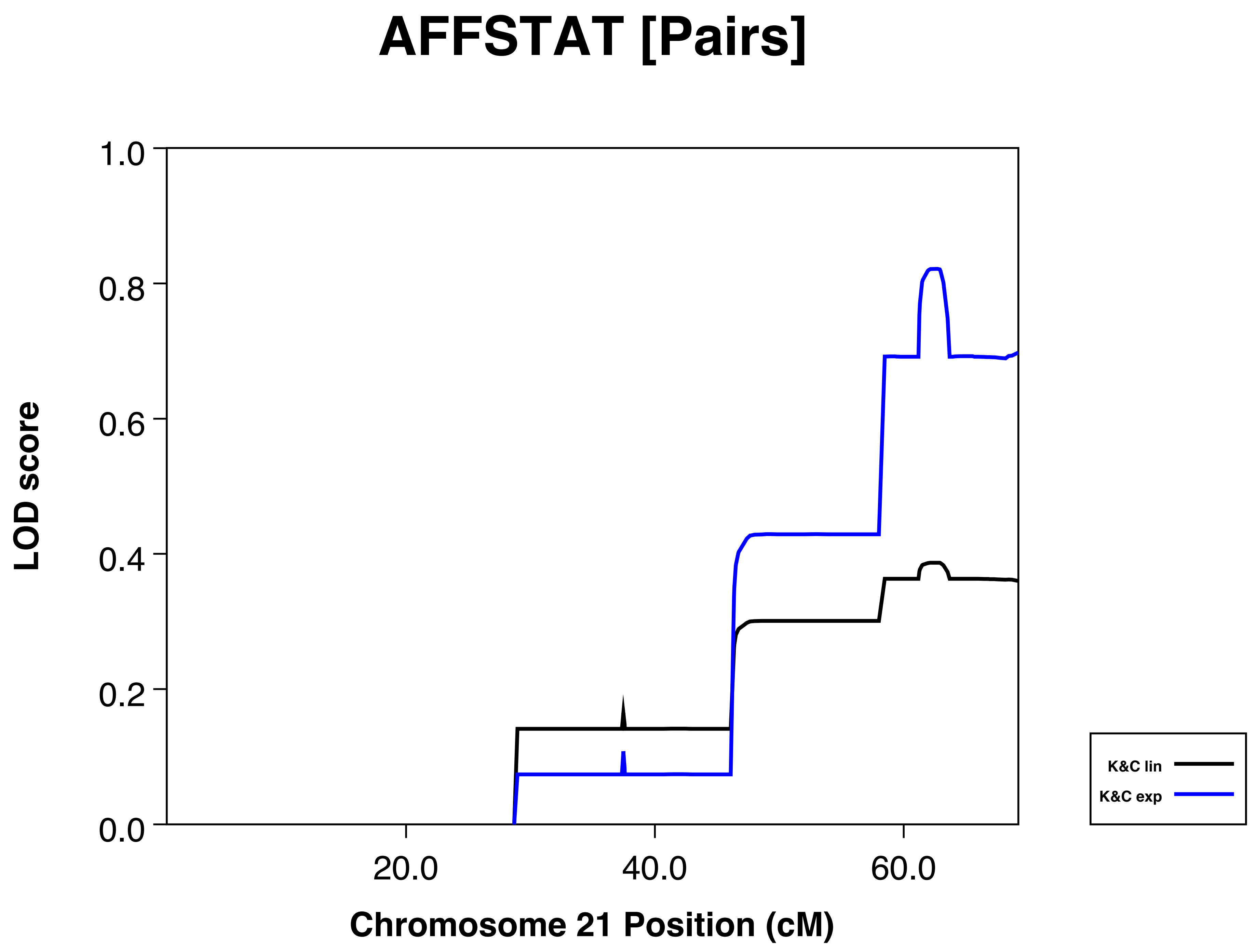
AFFSTAT [Pairs]
1.0
0.8
0.6
0.4
0.2
0.0
LOD score
20.0
40.0
60.0
Chromosome 21 Position (cM)
K&C lin
K&C exp

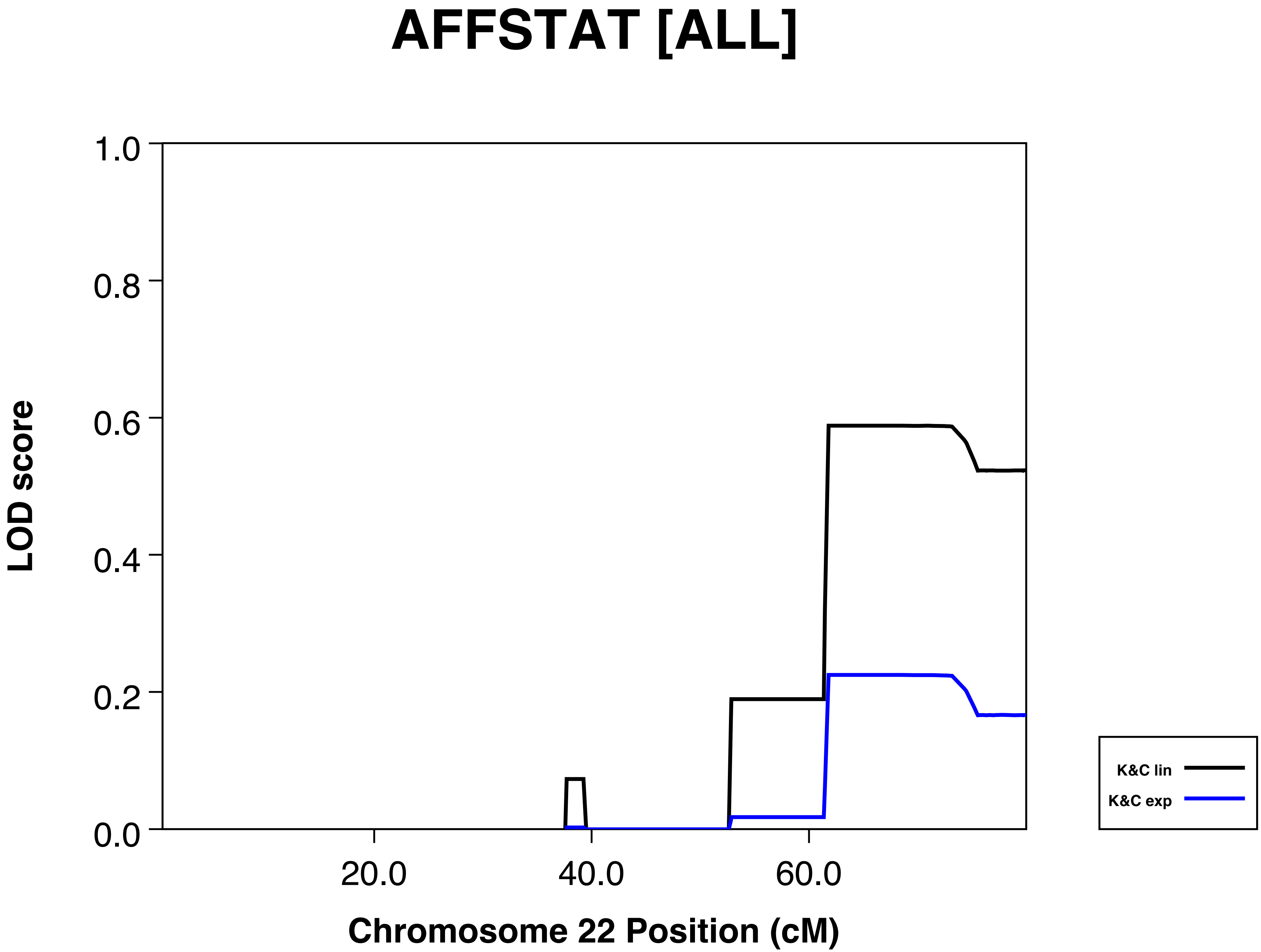
AFFSTAT [ALL]
LOD score
1.0
0.8
0.6
0.4
0.2
0.0
20.0
40.0
60.0
Chromosome 22 Position (cM)
K&C lin
K&C exp

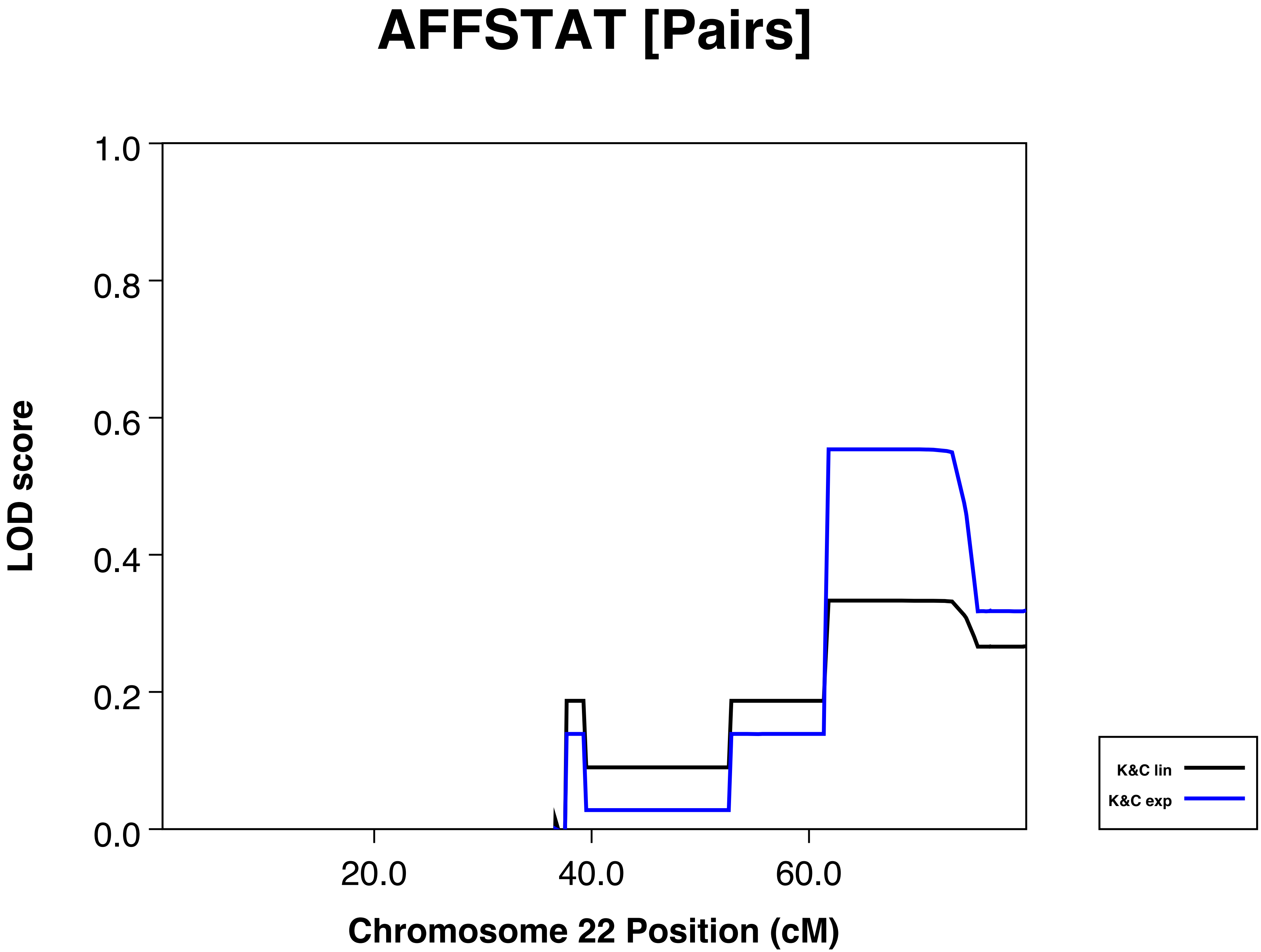
AFFSTAT [Pairs]
1.0
0.8
0.6
0.4
0.2
0.0
LOD score
20.0
40.0
60.0
Chromosome 22 Position (cM)
K&C lin
K&C exp
