# Supplementary material for: CELSR2 is a candidate susceptibility gene in idiopathic scoliosis
Source: PLoS One. 2017 Dec 14;12(12):e0189591. doi: 10.1371/journal.pone.0189591 (PMC5730153; doi:10.1371/journal.pone.0189591)
Supplement: S2 Fig — The output QC for the exome sequencing of individuals II:I (up101_1) and II:III (up101_2). (PDF) [file pone.0189591.s002.pdf]

SUPPLEMENTARY FIGURE 2

Run Report for Auto_user_PR5-38-up_101_73

Run Summary

13.8 G	78
Total Bases	Key Signal

74%
ISP Loading

ISP Density

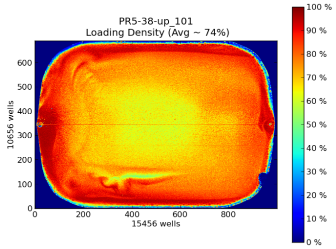

77,519,234
Total Reads

72%
Usable Reads

ISP Summary

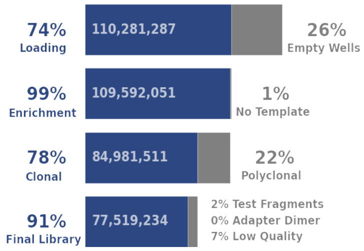

None bp	196 bp	227 bp
Mean	Median	Mode

Read Length

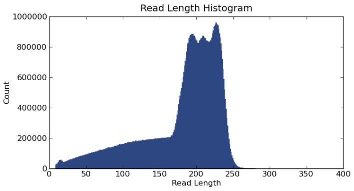

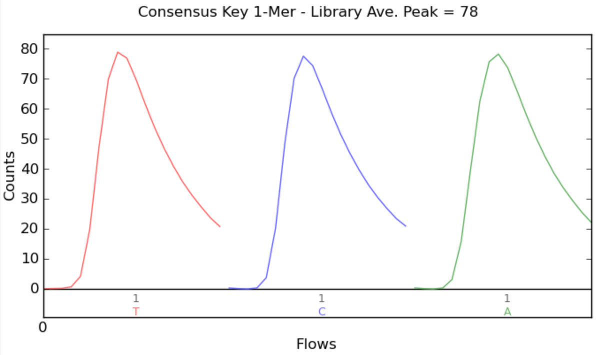

**Addressable Wells**	**148,603,956**	
With ISPs	110,281,287	74.2%
Live	109,592,051	99.4%
Test Fragment	1,714,714	01.6%
Library	107,877,337	98.4%

**Library ISPs**	**107,877,337**	
Filtered: Polyclonal	24,610,540	22.8%
Filtered: Low Quality	6,222,498	05.8%
Filtered: Primer Dimer	24,896	00.0%
**Final Library ISPs**	**77,519,234**	**71.9%**

Barcode Name	Sample	Bases	$\geq Q20$	Reads	Mean Read Length
No barcode	none	386,477,203	319,727,981	2,087,093	185 bp
IonXpress_014	up_101_1	6,588,730,342	5,466,721,023	37,002,135	178 bp
IonXpress_015	up_101_2	6,848,130,672	5,697,152,068	38,390,705	178 bp

Test Fragment	Reads	Percent 50AQ17	Read Length Histogram
**TF_C**	**1,429,611**	**77%**	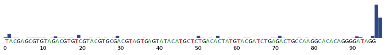

ion torrent
by life technologies

Alignment Summary *(aligned to Homo sapiens)*

**13.2 G**	**4.3X**
Total Alignment Bases	Average Coverage Depth of Reference

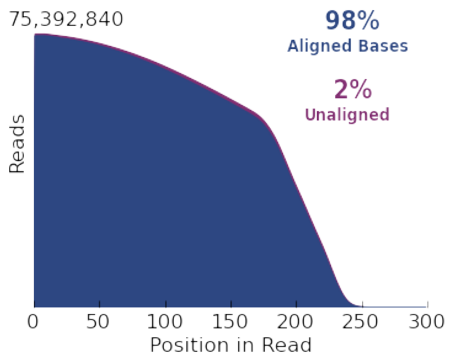

98.7%
Mean Raw Accuracy 1x

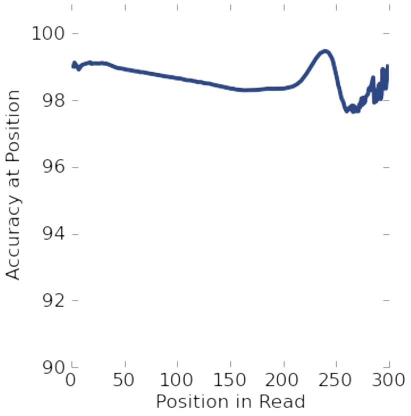

	AQ17	AQ20	Perfect
**Total Number of Bases [Mbp]**	11.8 G	10.2 G	7.4 G
**Mean Length [bp]**	168	152	116
**Longest Alignment [bp]**	332	332	306
**Mean Coverage Depth**	3.8	3.3	2.4

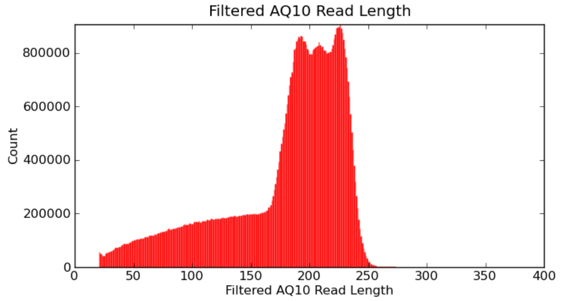

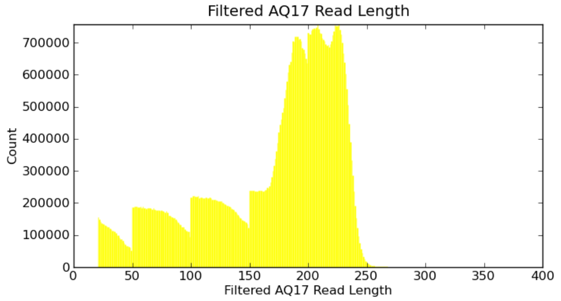

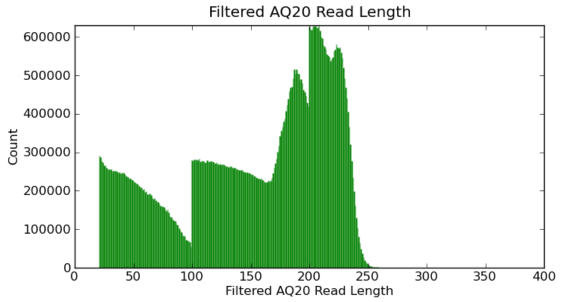

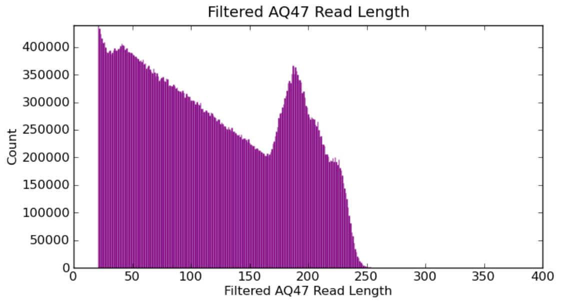

coverageAnalysis

Library type: Ion AmpliSeq Exome
Target regions: AmpliSeqExome.20130612.Designed

Barcode Name	Sample	Mapped Reads	On Target	Mean Depth	Uniformity

variantCaller

Library type:	AmpliSeq
Targeted regions:	AmpliSeqExome.20130612.Designed
Hotspot regions:	None
Configuration:	Generic - Proton - Germ Line - Low Stringency
Download all barcodes:	VCF.ZIP XLS.ZIP XLS

Barcode Name	Sample Name	Variants	Download Links
IonXpress_014	up_101_1	53680	VCF.GZ VCF.GZ.TBI XLS
IonXpress_015	up_101_2	53599	VCF.GZ VCF.GZ.TBI XLS

ion torrent
by life technologies™

Analysis Details

**Run Name**	R_2014_09_26_13_59_17_user_PR5-38-up_101
**Run Date**	Sept. 26, 2014, 2:01 p.m.
**Run Flows**	520
**Projects**	
**Sample**	up_101_2, up_101_1
**Reference**	
**PGM**	PR5
**Flow Order**	TACGTACGTCTGAGCATCGATCGATGTACAGC
**Library Key**	TCAG
**TF Key**	ATCG
**Chip Check**	Passed
**Chip Type**	P1.1.17
**Chip Data**	tiled
**Barcode Set**	IonXpress
**Analysis Name**	Auto_user_PR5-38-up_101_73
**Analysis Date**	Sept. 27, 2014, 1:03 a.m.
**Analysis Flows**	0
**runID**	ZBN5S

Software Version

**Torrent_Suite**	4.2.1
**host**	Proton05
**ion-analysis**	4.2.18-1
**ion-chefupdates**	4.2.0
**ion-dbreports**	4.2.22-1
**ion-gpu**	4.2.2-1
**ion-pipeline**	4.2.12-1
**ion-plugins**	4.2.28-1
**ion-protonupdates**	4.2.2
**ion-torrentr**	4.2.1-1
**LiveView**	1835
**DataCollect**	2929
**OIA**	4201
**OS**	21
**Graphics**	34
