# Supplementary figures and images for: CELSR2 is a candidate susceptibility gene in idiopathic scoliosis
Source: PLoS One. 2017 Dec 14;12(12):e0189591. doi: 10.1371/journal.pone.0189591 (PMC5730153; doi:10.1371/journal.pone.0189591)

A  
Text

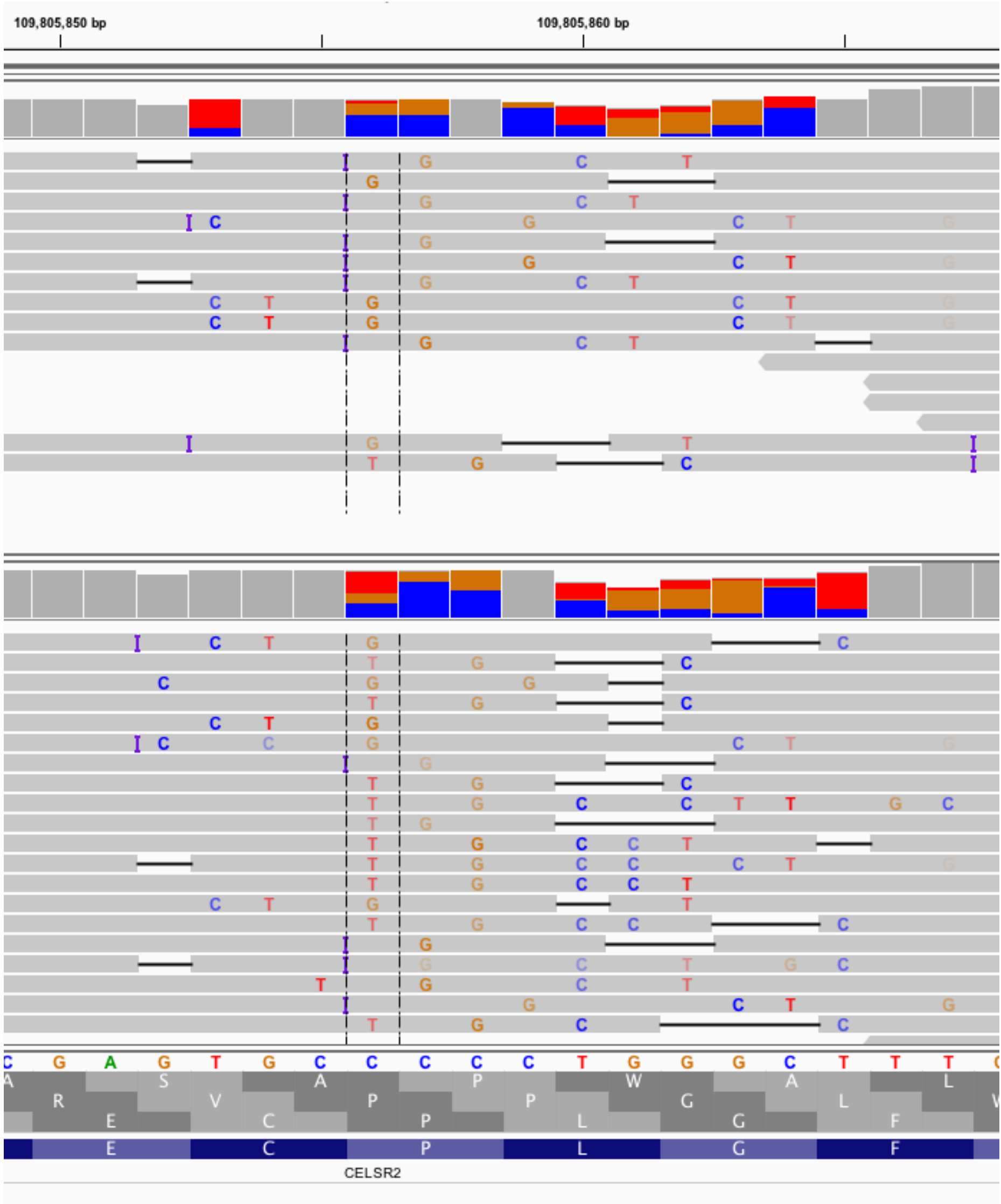

B

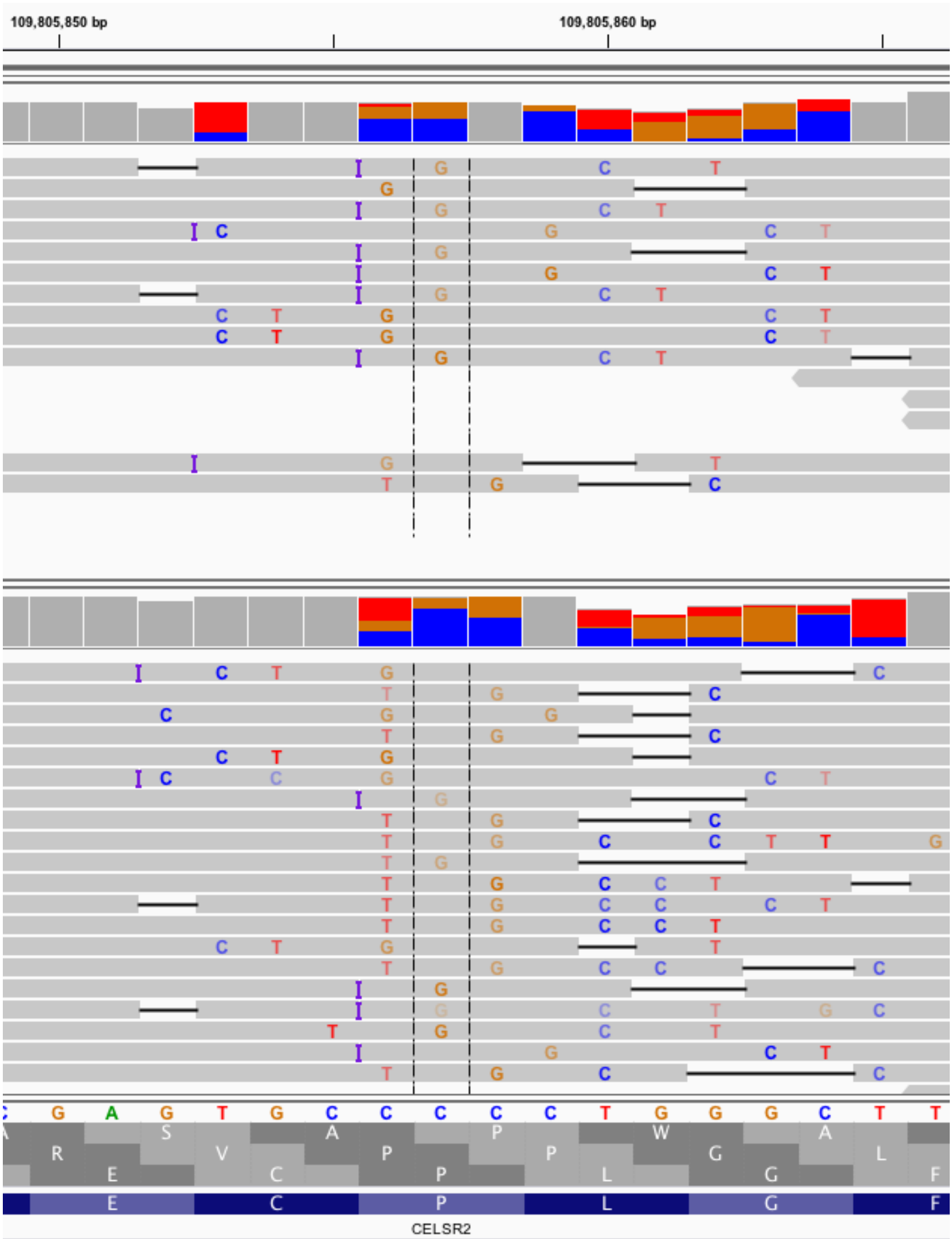

C

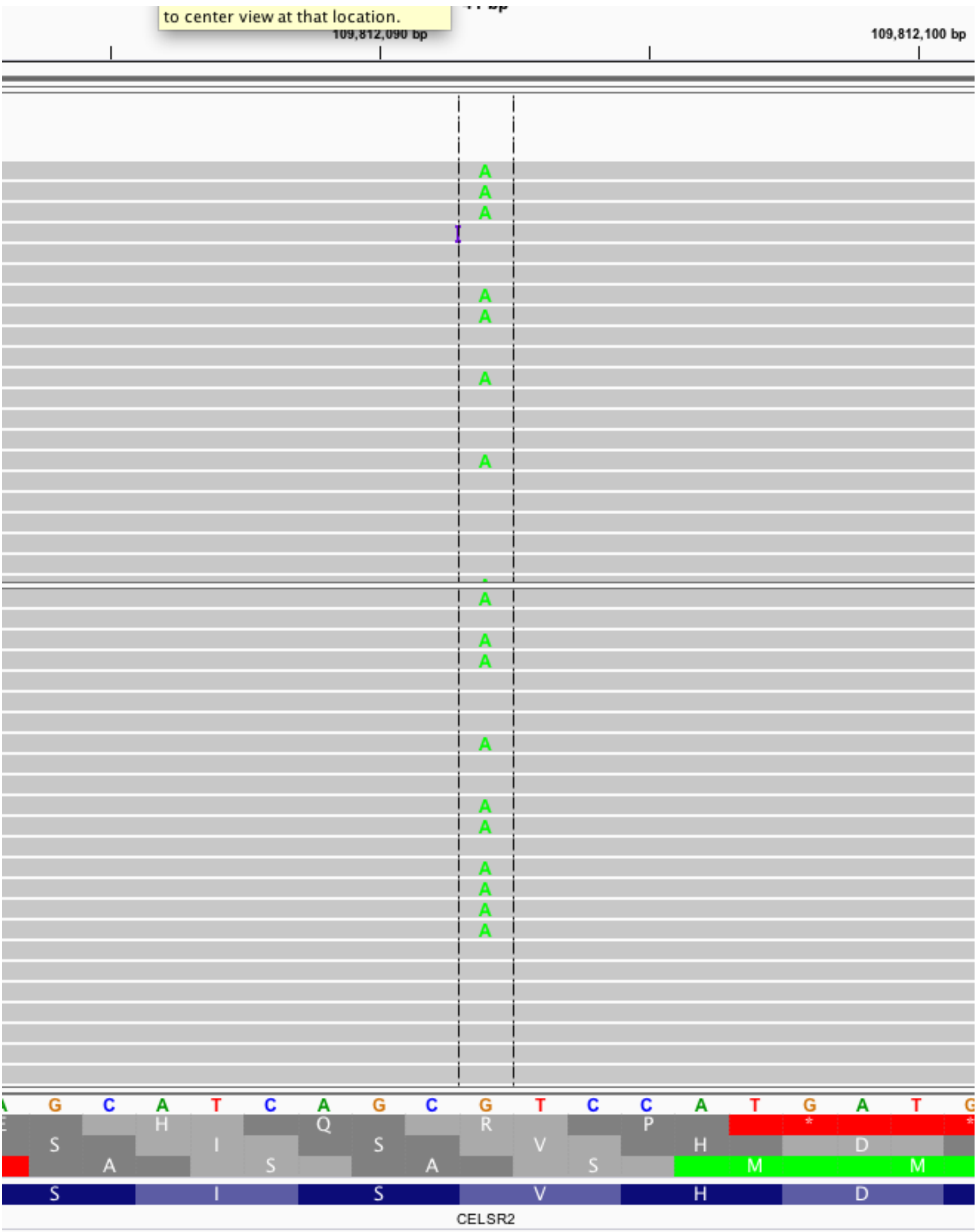

Supplement: S3 Fig — (software.broadinstitute.org/software/igv/), Figure shows the three putative rare CELSR2 variants shared by both sequenced individuals (upper: II:I, lower: II:III). Panel A shows a putative 1:109805856, C>T variant, panel B shows a putative 1:109805857, C>G variant. Both reside within the same region of low quality sequence and were deemed to be false positive variant calls. Panel C shows the 1:109812092, G>A variant, confirmed by Sanger sequencing to be a true variant. (PDF) [file pone.0189591.s003.pdf]

SUPPLEMENTARY FIGURE 4

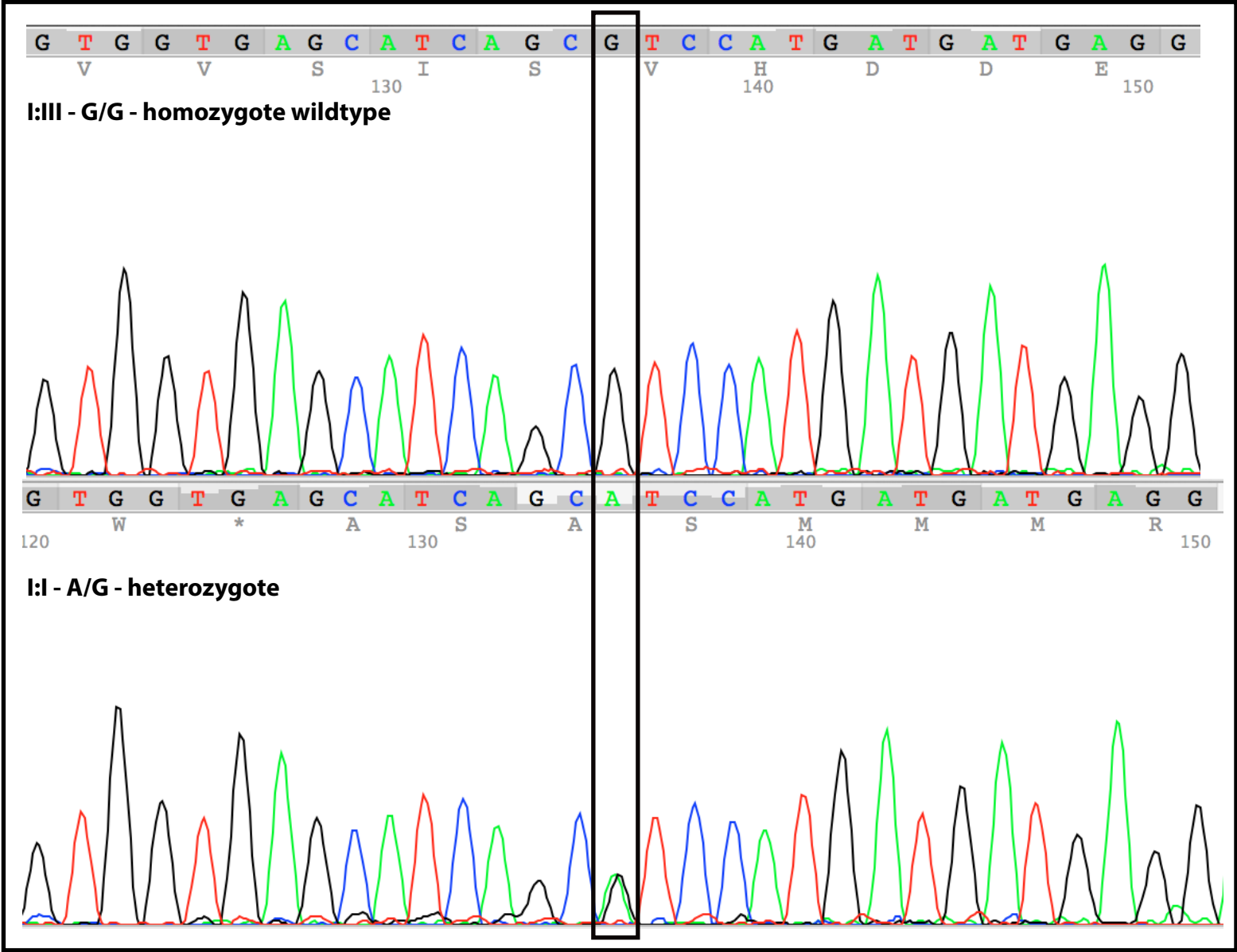

Supplement: S4 Fig — CELSR2 1:109812092, G>A variant shown by Sanger sequencing. Upper panel shows the wildtype G/G genotype (in I:III), lower panel shows an A/G heterozygote (I:I). (PDF) [file pone.0189591.s004.pdf]

# SUPPLEMENTARY FIGURE 6

Text

Plotted SNPs

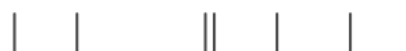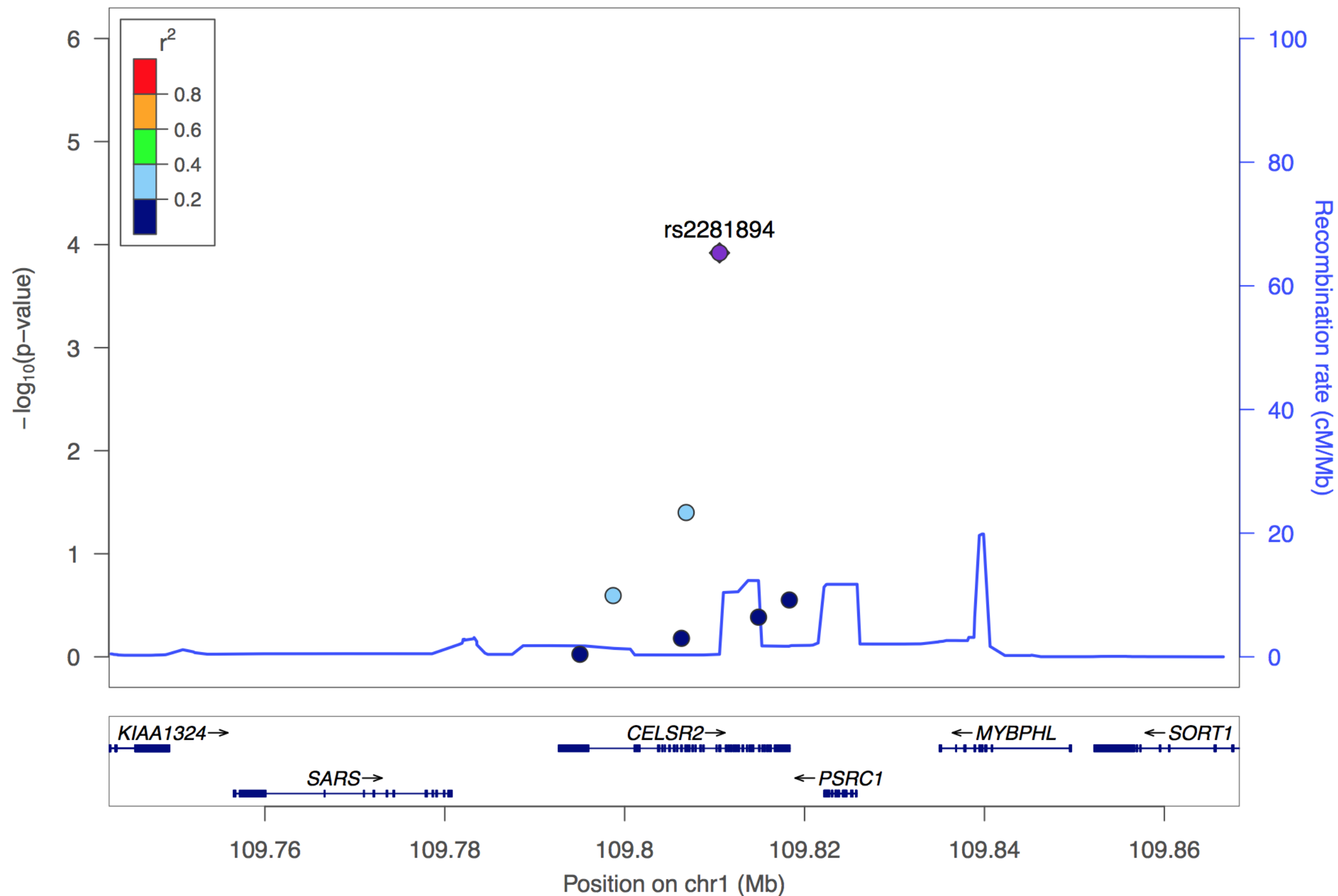

Supplement: S6 Fig — Association of tagging variants in CELSR2 with idiopathic scoliosis in a Swedish case-control dataset. The plot is produced using LocusZoom, available at locuszoom.org. The x-axis shows the position of the variants on the chromosome (in Mb), and relative to CELSR2 and neighbouring genes. The left y-axis shows the -log of the association. The most strongly associated variant, rs2281894, is marked with a diamond; the colour of the other markers (circles) is determined by their linkage disequilibrium (LD) with rs228194 (based on hg19/1000Genomes Nov2014 EUR). The right y-axis shows the recombination rate in the region as a light blue line. (PDF) [file pone.0189591.s006.pdf]
