# Supplementary material for: CELSR2 is a candidate susceptibility gene in idiopathic scoliosis
Source: PLoS One. 2017 Dec 14;12(12):e0189591. doi: 10.1371/journal.pone.0189591 (PMC5730153; doi:10.1371/journal.pone.0189591)
Supplement: S5 Fig — Conservation of the CELSR2 protein between humans (HUMAN), mice (MOUSE), rat (RAT), dog (CANLF), CAVPO (guinea pig), OTOGA (small-eared galago), BOVIN (bovine), AILME (giant panda), and rabbit (RABIT). The location of the human V2287 is marked in red. (PDF) [file pone.0189591.s005.pdf]

SUPPLEMENTARY FIGURE 5

```
Q9HCU4 CELR2_HUMAN   2270 LRVPKRPIINTPVVSISVHDDEELLPRALDKPVTVQFRLLETEERTKPICVFWNHSILVS 2329
Q9R0M0 CELR2_MOUSE   2271 LRVPKRPVINTPAVSISVHDDEELLPRALDKPVTVQFRLLETEERTKPICVFWNHSILVS 2330
Q9QYP2 CELR2_RAT     1495 LRVPKRPVINTPVVSISVHDDEELLPRALDKPVTVQFRLLETEERTKPICVFWNHSILVS 1554
E2R4F0 E2R4F0_CANLF  2273 LRVPKRPVINTPVVSISVHDDEELLPRALDKPVTVQFRLLETEERTKPICVFWNHSILVS 2332
H0VTZ9 H0VTZ9_CAVPO  2273 LRVPKRPVINTPVVSISVHDDEELLPRALDKPVTVQFRLLETEERTKPICVFWNHSILVS 2332
H0X9A5 H0X9A5_OTOGA  2268 LRVPKRPVINTPVVSVSVHDDEELLPRALDKPVTVQFRLLETEERTKPICVFWNHSILVS 2327
E1BJ11 E1BJ11_BOVIN  2265 LRVPKRPVINTPVVSISVHDDEELLPRALDKPVTVQFRLLETEERTKPICVFWNHSILVS 2324
G1LDF3 G1LDF3_AILME  2272 LRVPKRPVINTPVVSISVHDDEELLPRALDKPVTVQFRLLETEERTKPICVFWNHSILVS 2331
G1TA45 G1TA45_RABIT  2264 LRVPKRPVINTPVVSISVHDDDELLPRSLDKPVTVQFRLLETEERTKPICVFWNHSILVS 2323
                          *******:****.**:*****:*****:********************************
```
