# Supplementary material for: CELSR2 is a candidate susceptibility gene in idiopathic scoliosis
Source: PLoS One. 2017 Dec 14;12(12):e0189591. doi: 10.1371/journal.pone.0189591 (PMC5730153; doi:10.1371/journal.pone.0189591)
Supplement: S1 Table — Phenotypic information on the family members included in the linkage/exome sequencing analysis. Individual numbering is as in Fig 1. (PDF) [file pone.0189591.s007.pdf]

SUPPLEMENTARY TABLE 1

Individual	Sex	Age at x-ray (years)	Largest Cobb (degree)	Apex vertebrae
I:I	Female	22	33	L1
II:I	Male	33	14	L2
II:II	Female	15	45	T9
II:III	Female	20	33/33	T7/L1
II:IV	Female	19	29	T8
II:V	Female	15	28	T9
II:VI	Female	13	29	T9
II:VII	Female	12	no scoliosis	-
I:II	Male	59	no scoliosis	-
I:III	Female	60	no scoliosis	-
II:VIII	Male	24	no scoliosis	-
I:IV	Female	53	no scoliosis	-
II:IX	Female	34	32	T10
II:X	Female	32	no scoliosis	-
II:XI	Female	28	26	T8
