# Supplementary material for: CELSR2 is a candidate susceptibility gene in idiopathic scoliosis
Source: PLoS One. 2017 Dec 14;12(12):e0189591. doi: 10.1371/journal.pone.0189591 (PMC5730153; doi:10.1371/journal.pone.0189591)
Supplement: S2 Table — Chromosome 1 Merlin most probable haplotypes flow (haplotypes coded as A, B, C etc, and colour coded for simplicity). Haplotypes are produced using Merlin, according to the most likely pattern of gene flow (—best) and using the—horizontal flag. (PDF) [file pone.0189591.s008.pdf]

SUPPLEMENTARY TABLE 2

Sample	Phenotype	Predicted haplo	1 RS12403322	2 RS17131049	3 RS10782817	4 RS970457	5 RS634904	6 RS680308	7 RS641965	8 RS2292191	9 RS4907093	10 RS10782526	11 RS1325273	12 RS12057637	13 RS1770691	14 RS709767	15 RS709790	16 RS2735592	17 RS17590006	18 RS6661365	19 RS10493768	20 RS1880209	21 RS12746325	22 RS608460	23 RS11161721	24 RS695051	25 RS11161735	26 RS10873770	27 RS263463	28 RS263492	29 RS12409961	30 RS10158460	31 RS11161973	32 RS17456070	33 RS751	34 RS1618898	35 RS12132154	36 RS17130163	37 RS11801253	38 RS10923030	39 RS2390439	40 RS12731063	41 RS1760206	42 RS7555039	43 RS10922605	44 RS4658359	45 RS359941	46 RS10922690	47 RS7552852	48 RS17130997	49 RS7518543	50 RS699539	51 RS6689845	52 RS4658186	53 RS605146
2	no sample, unknown	A	A	A	A	A	A	A	A	A	A	A	A	A	A	A	A	A	A	A	A	A	A	A	A	A	A	A	A	A	A	A	A	A	A	A	A	A	A	A	A	A	A	A	A	A	A	A	A	A	A	A	A	A	A
2	no sample, unknown	B	B	B	B	B	B	B	B	B	B	B	B	B	B	B	B	B	B	B	B	B	B	B	B	B	B	B	B	B	B	B	B	B	B	B	B	B	B	B	B	B	B	B	B	B	B	B	B	B	B	B	B	B	B
10	no sample, unknown	C	C	C	C	C	C	C	C	C	C	C	C	C	C	C	C	C	C	C	C	C	C	C	C	C	C	C	C	C	C	C	C	C	C	C	C	C	C	C	C	C	C	C	C	C	C	C	C	C	C	C	C	C	C
10	no sample, unknown	D	D	D	D	D	D	D	D	D	D	D	D	D	D	D	D	D	D	D	D	D	D	D	D	D	D	D	D	D	D	D	D	D	D	D	D	D	D	D	D	D	D	D	D	D	D	D	D	D	D	D	D	D	D
11	no sample, unknown	E	E	E	E	E	E	E	E	E	E	E	E	E	E	E	E	E	E	E	E	E	E	E	E	E	E	E	E	E	E	E	E	E	E	E	E	E	E	E	E	E	E	E	E	E	E	E	E	E	E	E	E	E	E
11	no sample, unknown	F	F	F	F	F	F	F	F	F	F	F	F	F	F	F	F	F	F	F	F	F	F	F	F	F	F	F	F	F	F	F	F	F	F	F	F	F	F	F	F	F	F	F	F	F	F	F	F	F	F	F	F	F	F
16	no sample,inmarried	G	G	G	G	G	G	G	G	G	G	G	G	G	G	G	G	G	G	G	G	G	G	G	G	G	G	G	G	G	G	G	G	G	G	G	G	G	G	G	G	G	G	G	G	G	G	G	G	G	G	G	G	G	G
16	no sample,inmarried	H	H	H	H	H	H	H	H	H	H	H	H	H	H	H	H	H	H	H	H	H	H	H	H	H	H	H	H	H	H	H	H	H	H	H	H	H	H	H	H	H	H	H	H	H	H	H	H	H	H	H	H	H	H
20	no sample,inmarried	I	I	I	I	I	I	I	I	I	I	I	I	I	I	I	I	I	I	I	I	I	I	I	I	I	I	I	I	I	I	I	I	I	I	I	I	I	I	I	I	I	I	I	I	I	I	I	I	I	I	I	I	I	I
20	no sample,inmarried	J	J	J	J	J	J	J	J	J	J	J	J	J	J	J	J	J	J	J	J	J	J	J	J	J	J	J	J	J	J	J	J	J	J	J	J	J	J	J	J	J	J	J	J	J	J	J	J	J	J	J	J	J	J
I:II	unaffected, carrier	E	E	E	E	E	E	E	E	E	E	E	E	E	E	E	E	E	E	E	E	E	E	E	E	E	E	E	E	E	E	E	E	E	E	E	E	E	E	E	E	E	E	E	E	E	E	E	E	E	E	E	E	E	E
I:II	unaffected, carrier	C/D	C	C	C	C	C	C	C	C	C	C	C	C	C	C	C	C	C	C	C	C	C	C	C	C	C	C	C	C	C	C	C	C	C	C	C	C	C	C	C	C	C	C	C	C	C	C	C	C	C	C	C	C	C
I:III	unaffected	F	F	F	F	F	F	F	F	F	F	F	F	F	F	F	F	F	F	F	F	F	F	F	F	F	F	F	F	F	F	F	F	F	F	F	F	F	F	F	F	F	F	F	F	F	F	F	F	F	F	F	F	F	F
I:III	unaffected	C	C	C	C	C	C	C	C	C	C	C	C	C	C	C	C	C	C	C	C	C	C	C	C	C	C	C	C	C	C	C	C	C	C	C	C	C	C	C	C	C	C	C	C	C	C	C	C	C	C	C	C	C	C
I:IV	unaffected, carrier	E	E	E	E	E	E	E	E	E	E	E	E	E	E	E	E	E	E	E	E	E	E	E	E	E	E	E	E	E	E	E	E	E	E	E	E	E	E	E	E	E	E	E	E	E	E	E	E	E	E	E	E	E	E
I:IV	unaffected, carrier	C/D/C	C	C	C	C	C	C	C	C	C	C	C	C	C	C	C	C	C	C	C	C	C	C	C	C	C	C	C	C	C	C	C	C	C	D	D	D	D	D	D	D	D	D	D	D	D	D	D	D	D	D	D	D	D
II:IX	affected	E	E	E	E	E	E	E	E	E	E	E	E	E	E	E	E	E	E	E	E	E	E	E	E	E	E	E	E	E	E	E	E	E	E	E	E	E	E	E	E	E	E	E	E	E	E	E	E	E	E	E	E	E	E
II:IX	affected	G/H	G	G	G	G	G	G	G	G	G	G	G	G	G	G	G	G	G	G	G	G	G	G	G	G	G	G	G	G	G	G	G	G	G	G	G	G	G	G	G	G	G	G	G	G	G	G	G	G	G	G	G	G	G
I:I	affected	E	E	E	E	E	E	E	E	E	E	E	E	E	E	E	E	E	E	E	E	E	E	E	E	E	E	E	E	E	E	E	E	E	E	E	E	E	E	E	E	E	E	E	E	E	E	E	E	E	E	E	E	E	E
I:I	affected	D	D	D	D	D	D	D	D	D	D	D	D	D	D	D	D	D	D	D	D	D	D	D	D	D	D	D	D	D	D	D	D	D	D	D	D	D	D	D	D	D	D	D	D	D	D	D	D	D	D	D	D	D	D
II:II	affected	E	E	E	E	E	E	E	E	E	E	E	E	E	E	E	E	E	E	E	E	E	E	E	E	E	E	E	E	E	E	E	E	E	E	E	E	E	E	E	E	E	E	E	E	E	E	E	E	E	E	E	E	E	E
II:II	affected	A	A	A	A	A	A	A	A	A	A	A	A	A	A	A	A	A	A	A	A	A	A	A	A	A	A	A	A	A	A	A	A	A	A	A	A	A	A	A	A	A	A	A	A	A	A	A	A	A	A	A	A	A	A
II:VI	affected	E	E	E	E	E	E	E	E	E	E	E	E	E	E	E	E	E	E	E	E	E	E	E	E	E	E	E	E	E	E	E	E	E	E	E	E	E	E	E	E	E	E	E	E	E	E	E	E	E	E	E	E	E	E
II:VI	affected	A	A	A	A	A	A	A	A	A	A	A	A	A	A	A	A	A	A	A	A	A	A	A	A	A	A	A	A	A	A	A	A	A	A	A	A	A	A	A	A	A	A	A	A	A	A	A	A	A	A	A	A	A	A
II:VIII	unaffected	F	F	F	F	F	F	F	F	F	F	F	F	F	F	F	F	F	F	F	F	F	F	F	F	F	F	F	F	F	F	F	F	F	F	F	F	F	F	F	F	F	F	F	F	F	F	F	F	F	F	F	F	F	F
II:VIII	unaffected	I	I	I	I	I	I	I	I	I	I	I	I	I	I	I	I	I	I	I	I	I	I	I	I	I	I	I	I	I	I	I	I	I	I	I	I	I	I	I	I	I	I	I	I	I	I	I	I	I	I	I	I	I	I
II:X	affected	E	E	E	E	E	E	E	E	E	E	E	E	E	E	E	E	E	E	E	E	E	E	E	E	E	E	E	E	E	E	E	E	E	E	E	E	E	E	E	E	E	E	E	E	E	E	E	E	E	E	E	E	E	E
II:X	affected	G	G	G	G	G	G	G	G	G	G	G	G	G	G	G	G	G	G	G	G	G	G	G	G	G	G	G	G	G	G	G	G	G	G	G	G	G	G	G	G	G	G	G	G	G	G	G	G	G	G	G	G	G	G
II:XI	unaffected	E/D/C	E	E	E	E	E	E	E	E	E	E	E	E	E	E	E	E	E	E	E	E	E	E	E	E	E	E	E	E	E	E	E	E	E	E	E	E	E	E	E	E	E	E	E	E	E	E	E	E	E	E	E	E	E
II:XI	unaffected	G	G	G	G	G	G	G	G	G	G	G	G	G	G	G	G	G	G	G	G	G	G	G	G	G	G	G	G	G	G	G	G	G	G	G	G	G	G	G	G	G	G	G	G	G	G	G	G	G	G	G	G	G	G
II:IV	affected	D/E/D	D	D	D	D	D	D	D	E	E	E	E	E	E	E	E	E	E	E	E	E	E	E	E	E	E	E	E	E	E	E	E	E	E	E	E	E	E	E	E	E	E	E	E	E	E	E	E	E	E	E	E	E	E
II:IV	affected	B	B	B	B	B	B	B	B	B	B	B	B	B	B	B	B	B	B	B	B	B	B	B	B	B	B	B	B	B	B	B	B	B	B	B	B	B	B	B	B	B	B	B	B	B	B	B	B	B	B	B	B	B	B
II:VII	unaffected, carrier	E	E	E	E	E	E	E	E	E	E	E	E	E	E	E	E	E	E	E	E	E	E	E	E	E	E	E	E	E	E	E	E	E	E	E	E	E	E	E	E	E	E	E	E	E	E	E	E	E	E	E	E	E	E
II:VII	unaffected, carrier	B	B	B	B	B	B	B	B	B	B	B	B	B	B	B	B	B	B	B	B	B	B	B	B	B	B	B	B	B	B	B	B	B	B	B	B	B	B	B	B	B	B	B	B	B	B	B	B	B	B	B	B	B	B
II:I	affected	E	E	E	E	E	E	E	E	E	E	E	E	E	E	E	E	E	E	E	E	E	E	E	E	E	E	E	E	E	E	E	E	E	E	E	E	E	E	E	E	E	E	E	E	E	E	E	E	E	E	E	E	E	E
II:I	affected	B	B	B	B	B	B	B	B	B	B	B	B	B	B	B	B	B	B	B	B	B	B	B	B	B	B	B	B	B	B	B	B	B	B	B	B	B	B	B	B	B	B	B	B	B	B	B	B	B	B	B	B	B	B
II:III	affected	E	E	E	E	E	E	E	E	E	E	E	E	E	E	E	E	E	E	E	E	E	E	E	E	E	E	E	E	E	E	E	E	E	E	E	E	E	E	E	E	E	E	E	E	E	E	E	E	E	E	E	E	E	E
II:III	affected	A	A	A	A	A	A	A	A	A	A	A	A	A	A	A	A	A	A	A	A	A	A	A	A	A	A	A	A	A	A	A	A	A	A	A	A	A	A	A	A	A	A	A	A	A	A	A	A	A	A	A	A	A	A
II:V	affected	E	E	E	E	E	E	E	E	E	E	E	E	E	E	E	E	E	E	E	E	E	E	E	E	E	E	E	E	E	E	E	E	E	E	E	E	E	E	E	E	E	E	E	E	E	E	E	E	E	E	E	E	E	E
II:V	affected	A	A	A	A	A	A	A	A	A	A	A	A	A	A	A	A	A	A	A	A	A	A	A	A	A	A	A	A	A	A	A	A	A	A	A	A	A	A	A	A	A	A	A	A	A	A	A	A	A	A	A	A	A	A

54	55	56	57	58	59	60	61	62	63	64	65	66	67	68	69	70	71	72	73	74	75	76	77	78	79	80	81	82	83	84	85	86	87	88	89	90	91	92	93	94	95	96	97	98	99	100	101	102	103	104	105	106	107	108	109	110	111	112
RS17485896	RS11164909	RS10874794	RS12123436	RS284143	RS10874981	RS563930	RS549938	RS237435	RS236316	RS12022530	RS718873	RS743113	RS4847196	RS570878	RS11165135	RS841675	RS841685	RS17411335	RS1245481	RS2802112	RS7534566	RS859040	RS933107	RS10874924	RS10127720	RS6593693	RS616243	RS1222069	RS12022566	RS6677997	RS12740489	RS6681958	RS17432093	RS12124488	RS641805	RS6684671	RS1577897	RS12123121	RS11165881	RS2297779	RS12756513	RS4554755	RS6679864	RS10875133	RS1155644	RS11165976	RS11166016	RS12759704	RS6692269	RS3850458	RS863686	RS1456169	RS17648010	RS17405754	RS7513463	RS834993	RS620281	RS7519706
A	A	A	A	A	A	A	A	A	A	A	A	A	A	A	A	A	A	A	A	A	A	A	A	A	A	A	A	A	A	A	A	A	A	A	A	A	A	A	A	A	A	A	A	A	A	A	A	A	A	A	A	A	A	A	A	A	A	A
B	B	B	B	B	B	B	B	B	B	B	B	B	B	B	B	B	B	B	B	B	B	B	B	B	B	B	B	B	B	B	B	B	B	B	B	B	B	B	B	B	B	B	B	B	B	B	B	B	B	B	B	B	B	B	B	B	B	B
C	C	C	C	C	C	C	C	C	C	C	C	C	C	C	C	C	C	C	C	C	C	C	C	C	C	C	C	C	C	C	C	C	C	C	C	C	C	C	C	C	C	C	C	C	C	C	C	C	C	C	C	C	C	C	C	C	C	C
D	D	D	D	D	D	D	D	D	D	D	D	D	D	D	D	D	D	D	D	D	D	D	D	D	D	D	D	D	D	D	D	D	D	D	D	D	D	D	D	D	D	D	D	D	D	D	D	D	D	D	D	D	D	D	D	D	D	D
E	E	E	E	E	E	E	E	E	E	E	E	E	E	E	E	E	E	E	E	E	E	E	E	E	E	E	E	E	E	E	E	E	E	E	E	E	E	E	E	E	E	E	E	E	E	E	E	E	E	E	E	E	E	E	E	E	E	E
F	F	F	F	F	F	F	F	F	F	F	F	F	F	F	F	F	F	F	F	F	F	F	F	F	F	F	F	F	F	F	F	F	F	F	F	F	F	F	F	F	F	F	F	F	F	F	F	F	F	F	F	F	F	F	F	F	F	F
G	G	G	G	G	G	G	G	G	G	G	G	G	G	G	G	G	G	G	G	G	G	G	G	G	G	G	G	G	G	G	G	G	G	G	G	G	G	G	G	G	G	G	G	G	G	G	G	G	G	G	G	G	G	G	G	G	G	G
H	H	H	H	H	H	H	H	H	H	H	H	H	H	H	H	H	H	H	H	H	H	H	H	H	H	H	H	H	H	H	H	H	H	H	H	H	H	H	H	H	H	H	H	H	H	H	H	H	H	H	H	H	H	H	H	H	H	H
I	I	I	I	I	I	I	I	I	I	I	I	I	I	I	I	I	I	I	I	I	I	I	I	I	I	I	I	I	I	I	I	I	I	I	I	I	I	I	I	I	I	I	I	I	I	I	I	I	I	I	I	I	I	I	I	I	I	I
J	J	J	J	J	J	J	J	J	J	J	J	J	J	J	J	J	J	J	J	J	J	J	J	J	J	J	J	J	J	J	J	J	J	J	J	J	J	J	J	J	J	J	J	J	J	J	J	J	J	J	J	J	J	J	J	J	J	J
E	E	E	E	E	E	E	E	E	E	E	E	E	E	E	E	E	E	E	E	E	E	E	E	E	E	E	E	E	E	E	E	E	E	E	E	E	E	E	E	E	E	E	E	E	E	E	E	E	E	E	E	E	E	E	E	E	E	E
C	C	C	C	C	C	C	C	C	C	C	C	C	C	C	C	C	C	C	C	C	C	C	C	C	C	C	C	C	C	C	C	C	C	C	C	C	C	C	C	C	C	C	C	C	C	C	C	C	C	C	C	C	C	C	C	C	C	C
F	F	F	F	F	F	F	F	F	F	F	F	F	F	F	F	F	F	F	F	F	F	F	F	F	F	F	F	F	F	F	F	F	F	F	F	F	F	F	F	F	F	F	F	F	F	F	F	F	F	F	F	F	F	F	F	F	F	F
C	C	C	C	C	C	C	C	C	C	C	C	C	C	C	C	C	C	C	C	C	C	C	C	C	C	C	C	C	C	C	C	C	C	C	C	C	C	C	C	C	C	C	C	C	C	C	C	C	C	C	C	C	C	C	C	C	C	C
E	E	E	E	E	E	E	E	E	E	E	E	E	E	E	E	E	E	E	E	E	E	E	E	E	E	E	E	E	E	E	E	E	E	E	E	E	E	E	E	E	E	E	E	E	E	E	E	E	E	E	E	E	E	E	E	E	E	E
D	D	D	D	D	D	D	D	D	D	D	D	D	D	D	C	C	C	C	C	C	C	C	C	C	C	C	C	C	C	C	C	C	C	C	C	C	C	C	C	C	C	C	C	C	C	C	C	C	C	C	C	C	C	C	C	C	C	C
E	E	E	E	E	E	E	E	E	E	E	E	E	E	E	E	E	E	E	E	E	E	E	E	E	E	E	E	E	E	E	E	E	E	E	E	E	E	E	E	E	E	E	E	E	E	E	E	E	E	E	E	E	E	E	E	E	E	E
G	G	G	G	G	G	G	G	G	G	G	G	G	G	G	G	G	G	G	G	G	G	G	G	G	G	G	G	G	G	G	G	G	G	G	G	G	G	G	G	G	G	G	G	G	G	G	G	G	G	G	G	G	G	G	G	G	G	G
E	E	E	E	E	E	E	E	E	E	E	E	E	E	E	E	E	E	E	E	E	E	E	E	E	E	E	E	E	E	E	E	E	E	E	E	E	E	E	E	E	E	E	E	E	E	E	E	E	E	E	E	E	E	E	E	E	E	E
D	D	D	D	D	D	D	D	D	D	D	D	D	D	D	D	D	D	D	D	D	D	D	D	D	D	D	D	D	D	D	D	D	D	D	D	D	D	D	D	D	D	D	D	D	D	D	D	D	D	D	D	D	D	D	D	D	D	D
E	E	E	E	E	E	E	E	E	E	E	E	E	E	E	E	E	E	E	E	E	E	E	E	E	E	E	E	E	E	E	E	E	E	E	E	E	E	E	E	E	E	E	E	E	E	E	E	E	E	E	E	E	E	E	E	E	E	E
A	A	A	A	A	A	A	A	A	A	A	A	A	A	A	A	A	A	A	A	A	A	A	A	A	A	A	A	A	A	A	A	A	A	A	A	A	A	A	A	A	A	A	A	A	A	A	A	A	A	A	A	A	A	A	A	A	A	A
E	E	E	E	E	E	E	E	E	E	E	E	E	E	E	E	E	E	E	E	E	E	E	E	E	E	E	E	E	E	E	E	E	E	E	E	E	E	E	E	E	E	E	E	E	E	E	E	E	E	E	E	E	E	E	E	E	E	E
A	A	A	A	A	A	A	A	A	A	A	A	A	A	A	A	A	A	A	A	A	A	A	A	A	A	A	A	A	A	A	A	A	A	A	A	A	A	A	A	A	A	A	A	A	A	A	A	A	A	A	A	A	A	A	A	A	A	A
F	F	F	F	F	F	F	F	F	F	F	F	F	F	F	F	F	F	F	F	F	F	F	F	F	F	F	F	F	F	F	F	F	F	F	F	F	F	F	F	F	F	F	F	F	F	F	F	F	F	F	F	F	F	F	F	F	F	F
I	I	I	I	I	I	I	I	I	I	I	I	I	I	I	I	I	I	I	I	I	I	I	I	I	I	I	I	I	I	I	I	I	I	I	I	I	I	I	I	I	I	I	I	I	I	I	I	I	I	I	I	I	I	I	I	I	I	I
E	E	E	E	E	E	E	E	E	E	E	E	E	E	E	E	E	E	E	E	E	E	E	E	E	E	E	E	E	E	E	E	E	E	E	E	E	E	E	E	E	E	E	E	E	E	E	E	E	E	E	E	E	E	E	E	E	E	E
G	G	G	G	G	G	G	G	G	G	G	G	G	G	G	G	G	G	G	G	G	G	G	G	G	G	G	G	G	G	G	G	G	G	G	G	G	G	G	G	G	G	G	G	G	G	G	G	G	G	G	G	G	G	G	G	G	G	G
E	E	E	E	E	E	E	E	E	E	E	E	E	D	D	C	C	C	C	C	C	C	C	C	C	C	C	C	C	C	C	C	C	C	C	C	C	C	C	C	C	C	C	C	C	C	C	C	C	C	C	C	C	C	C	C	C	C	C
G	G	G	G	G	G	G	G	G	G	G	G	G	G	G	G	G	G	G	G	G	G	G	G	G	G	G	G	G	G	G	G	G	G	G	G	G	G	G	G	G	G	G	G	G	G	G	G	G	G	G	G	G	G	G	G	G	G	G
E	E	E	E	E	E	E	E	E	E	E	E	E	E	E	E	E	E	E	E	E	E	E	E	E	E	E	E	E	E	E	E	E	E	E	E	E	E	E	E	E	E	E	E	E	E	E	E	E	E	E	E	E	E	E	E	E	E	E
B	B	B	B	B	B	B	B	B	B	B	B	B	B	B	B	B	B	B	B	B	B	B	B	B	B	B	B	B	B	B	B	B	B	B	B	B	B	B	B	B	B	B	B	B	B	B	B	B	B	B	B	B	B	B	B	B	B	B
E	E	E	E	E	E	E	E	E	E	E	E	E	E	E	E	E	E	E	E	E	E	E	E	E	E	E	E	E	E	E	E	E	E	E	E	E	E	E	E	E	E	E	E	E	E	E	E	E	E	E	E	E	E	E	E	E	E	E
B	B	B	B	B	B	B	B	B	B	B	B	B	B	B	B	B	B	B	B	B	B	B	B	B	B	B	B	B	B	B	B	B	B	B	B	B	B	B	B	B	B	B	B	B	B	B	B	B	B	B	B	B	B	B	B	B	B	B
E	E	E	E	E	E	E	E	E	E	E	E	E	E	E	E	E	E	E	E	E	E	E	E	E	E	E	E	E	E	E	E	E	E	E	E	E	E	E	E	E	E	E	E	E	E	E	E	E	E	E	E	E	E	E	E	E	E	E
B	B	B	B	B	B	B	B	B	B	B	B	B	B	B	B	B	B	B	B	B	B	B	B	B	B	B	B	B	B	B	B	B	B	B	B	B	B	B	B	B	B	B	B	B	B	B	B	B	B	B	B	B	B	B	B	B	B	B
E	E	E	E	E	E	E	E	E	E	E	E	E	E	E	E	E	E	E	E	E	E	E	E	E	E	E	E	E	E	E	E	E	E	E	E	E	E	E	E	E	E	E	E	E	E	E	E	E	E	E	E	E	E	E	E	E	E	E
A	A	A	A	A	A	A	A	A	A	A	A	A	A	A	A	A	A	A	A	A	A	A	A	A	A	A	A	A	A	A	A	A	A	A	A	A	A	A	A	A	A	A	A	A	A	A	A	A	A	A	A	A	A	A	A	A	A	A
E	E	E	E	E	E	E	E	E	E	E	E	E	E	E	E	E	E	E	E	E	E	E	E	E	E	E	E	E	E	E	E	E	E	E	E	E	E	E	E	E	E	E	E	E	E	E	E	E	E	E	E	E	E	E	E	E	E	E
A	A	A	A	A	A	A	A	A	A	A	A	A	A	A	A	A	A	A	A	A	A	A	A	A	A	A	A	A	A	A	A	A	A	A	A	A	A	A	A	A	A	A	A	A	A	A	A	A	A	A	A	A	A	A	A	A	A	A

113	114	115	116	117	118	119	120	121	122	123	124	125	126	127	128	129	130	131	132	133	134	135	136	137	138
RS482466	RS478859	RS7516666	RS17414954	RS1887101	RS10218565	RS1041312	RS4341378	RS6676516	RS4907932	RS6577246	RS3131386	RS1484308	RS4908187	RS9324336	RS4907956	RS11164337	RS12407943	RS950061	RS12723721	RS6577353	RS2050592	RS12132911	RS12144221	RS11184169	RS12076721
A	A	A	A	A	A	A	A	A	A	A	A	A	A	A	A	A	A	A	A	A	A	A	A	A	A
B	B	B	B	B	B	B	B	B	B	B	B	B	B	B	B	B	B	B	B	B	B	B	B	B	B
C	C	C	C	C	C	C	C	C	C	C	C	C	C	C	C	C	C	C	C	C	C	C	C	C	C
D	D	D	D	D	D	D	D	D	D	D	D	D	D	D	D	D	D	D	D	D	D	D	D	D	D
E	E	E	E	E	E	E	E	E	E	E	E	E	E	E	E	E	E	E	E	E	E	E	E	E	E
F	F	F	F	F	F	F	F	F	F	F	F	F	F	F	F	F	F	F	F	F	F	F	F	F	F
G	G	G	G	G	G	G	G	G	G	G	G	G	G	G	G	G	G	G	G	G	G	G	G	G	G
H	H	H	H	H	H	H	H	H	H	H	H	H	H	H	H	H	H	H	H	H	H	H	H	H	H
I	I	I	I	I	I	I	I	I	I	I	I	I	I	I	I	I	I	I	I	I	I	I	I	I	I
J	J	J	J	J	J	J	J	J	J	J	J	J	J	J	J	J	J	J	J	J	J	J	J	J	J
E	E	E	E	E	E	E	E	E	E	E	E	E	E	E	E	E	E	E	E	E	E	E	E	E	E
C	C	C	C	C	C	C	C	C	C	C	C	C	C	C	C	C	C	C	C	C	C	C	C	C	C
F	F	F	F	F	F	F	F	F	F	F	F	F	F	F	F	F	F	F	F	F	F	F	F	F	F
C	C	C	C	C	C	C	C	C	C	C	C	C	C	C	C	C	C	C	C	C	C	C	C	C	C
E	E	E	E	E	E	E	E	E	E	E	E	E	E	E	E	E	E	E	E	E	E	E	E	E	E
C	C	C	C	C	C	C	C	C	C	C	C	C	C	C	C	C	C	C	C	C	C	C	C	C	C
E	E	E	E	E	E	E	E	E	E	E	E	E	E	E	E	E	E	E	E	E	E	E	E	E	E
G	G	G	G	G	G	G	G	G	G	H	H	H	H	H	H	H	H	H	H	H	H	H	H	H	H
E	E	E	E	E	E	E	E	E	E	E	E	E	E	E	E	E	E	E	E	E	E	E	E	E	E
D	D	D	D	D	D	D	D	D	D	D	D	D	D	D	D	D	D	D	D	D	D	D	D	D	D
E	E	E	E	E	E	E	E	E	E	E	E	E	E	E	E	E	E	E	E	E	E	E	E	E	E
A	A	A	A	A	A	A	A	A	A	A	A	A	A	A	A	A	A	A	A	A	A	A	A	A	A
E	E	E	E	E	E	E	E	E	E	E	E	E	E	E	E	E	E	E	E	E	E	E	E	E	E
A	A	A	A	A	A	A	A	A	A	A	A	A	A	A	A	A	A	A	A	A	A	A	A	A	A
F	F	F	F	F	F	F	F	F	F	F	F	F	F	F	F	F	F	F	F	F	F	F	F	F	F
I	I	I	I	I	I	I	I	I	I	I	I	I	I	I	I	I	I	I	I	I	I	I	I	I	I
E	E	E	E	E	E	E	E	E	E	E	E	E	E	E	E	E	E	E	E	E	E	E	E	E	E
G	G	G	G	G	G	G	G	G	G	G	G	G	G	G	G	G	G	G	G	G	G	G	G	G	G
C	C	C	C	C	C	C	C	C	C	C	C	C	C	C	C	C	C	C	C	C	C	C	C	C	C
G	G	G	G	G	G	G	G	G	G	G	G	G	G	G	G	G	G	G	G	G	G	G	G	G	G
E	E	E	E	E	E	E	E	E	E	E	E	E	E	E	E	E	E	E	E	E	E	E	E	E	E
B	B	B	B	B	B	B	B	B	B	B	B	B	B	B	B	B	B	B	B	B	B	B	B	B	B
E	E	E	E	E	E	E	E	E	E	E	E	E	E	E	E	E	E	E	E	E	E	E	E	E	E
B	B	B	B	B	B	B	B	B	B	B	B	B	B	B	B	B	B	B	B	B	B	B	B	B	B
E	E	E	E	E	E	E	E	E	E	E	E	E	E	E	E	E	E	E	E	E	E	E	E	E	E
B	B	B	B	B	B	B	B	B	B	B	B	B	B	B	B	B	B	B	B	B	B	B	B	B	B
E	E	E	E	E	E	E	E	E	E	E	E	E	E	E	E	E	E	E	E	E	E	E	E	E	E
A	A	A	A	A	A	A	A	A	A	A	A	A	A	A	A	A	A	A	A	A	A	A	A	A	A
E	E	E	E	E	E	E	E	E	E	E	E	E	E	E	E	E	E	E	E	E	E	E	E	E	E
A	A	A	A	A	A	A	A	A	A	A	A	A	A	A	A	A	A	A	A	A	A	A	A	A	A

139	140	141	142	143	144	145	146	147	148	149	150	151	152	153	154	155	156	157	158	159	160	161	162	163	164
RS6583061	RS12741933	RS10881264	RS2842217	RS11184717	RS3850613	RS11184747	RS6702428	RS4915125	RS6677772	RS6583000	RS10494056	RS9628993	RS3125673	RS746603	RS920835	RS633869	RS7537311	RS7541272	RS17019855	RS11808011	RS11185183	RS1020812	RS11185272	RS7522260	RS10776762
A	A	A	A	A	A	A	A	A	A	A	A	A	A	A	A	A	A	A	A	A	A	A	A	A	A
B	B	B	B	B	B	B	B	B	B	B	B	B	B	B	B	B	B	B	B	B	B	B	B	B	B
C	C	C	C	C	C	C	C	C	C	C	C	C	C	C	C	C	C	C	C	C	C	C	C	C	C
D	D	D	D	D	D	D	D	D	D	D	D	D	D	D	D	D	D	D	D	D	D	D	D	D	D
E	E	E	E	E	E	E	E	E	E	E	E	E	E	E	E	E	E	E	E	E	E	E	E	E	E
F	F	F	F	F	F	F	F	F	F	F	F	F	F	F	F	F	F	F	F	F	F	F	F	F	F
G	G	G	G	G	G	G	G	G	G	G	G	G	G	G	G	G	G	G	G	G	G	G	G	G	G
H	H	H	H	H	H	H	H	H	H	H	H	H	H	H	H	H	H	H	H	H	H	H	H	H	H
I	I	I	I	I	I	I	I	I	I	I	I	I	I	I	I	I	I	I	I	I	I	I	I	I	I
J	J	J	J	J	J	J	J	J	J	J	J	J	J	J	J	J	J	J	J	J	J	J	J	J	J
E	E	E	E	E	E	E	E	E	E	E	E	E	E	E	E	E	E	E	E	E	E	E	E	E	E
C	C	C	C	C	C	C	C	C	C	C	C	C	C	C	C	C	C	C	C	C	C	C	C	C	C
F	F	F	F	F	F	F	F	F	F	F	F	F	F	F	F	F	F	F	F	F	F	F	F	F	F
C	C	C	C	C	C	C	C	C	C	C	C	C	C	C	C	C	C	C	C	C	C	C	C	C	C
E	E	E	E	E	E	E	E	E	E	E	E	E	E	E	E	E	E	E	E	E	E	E	E	E	E
C	C	C	C	C	C	C	C	C	C	C	C	C	C	C	C	C	C	C	C	C	C	C	C	C	C
E	E	E	E	E	E	E	E	E	E	E	E	E	E	E	E	E	E	E	E	E	E	E	E	E	E
H	H	H	H	H	H	H	H	H	H	H	H	H	H	H	H	H	H	H	H	H	H	H	H	H	H
E	E	E	E	E	E	E	E	E	E	E	E	E	E	E	E	E	E	E	E	E	E	E	E	E	E
D	D	D	D	D	D	D	D	D	D	D	D	D	D	D	D	D	D	D	D	D	D	D	D	D	D
E	E	E	E	E	E	E	E	E	E	E	E	E	E	E	E	E	E	E	E	E	E	E	E	E	E
A	A	A	A	A	A	A	A	A	A	A	A	A	A	A	A	A	A	A	A	A	A	A	A	A	A
E	E	E	E	E	E	E	E	E	E	E	E	E	E	E	E	E	E	E	E	E	E	E	E	E	E
A	A	A	A	A	A	A	A	A	A	A	A	A	A	A	A	A	A	A	A	A	A	A	A	A	A
F	F	F	F	F	F	F	F	F	F	F	F	F	F	F	F	F	F	F	F	F	F	F	F	F	F
I	I	I	I	I	I	I	I	I	I	I	I	I	I	I	I	I	I	I	I	I	I	I	I	I	I
E	E	E	E	E	E	E	E	E	E	E	E	E	E	E	E	E	E	E	E	E	E	E	E	E	E
G	G	G	G	G	G	G	G	G	G	G	G	G	G	G	G	G	G	G	G	G	G	G	G	G	G
C	C	C	C	C	C	C	C	C	C	C	C	C	C	C	C	C	C	C	C	C	C	C	C	C	C
G	G	G	G	G	G	G	G	G	G	G	G	G	G	G	G	G	G	G	G	G	G	G	G	G	G
E	E	E	E	E	E	E	E	E	E	E	E	E	E	E	E	E	E	E	E	E	E	E	E	E	E
B	B	B	B	B	B	B	B	B	B	B	B	B	B	B	B	B	B	B	B	B	B	B	B	B	B
E	E	E	E	E	E	E	E	E	E	E	E	E	E	E	E	E	E	E	E	E	E	E	E	E	E
B	B	B	B	B	B	B	B	B	B	B	B	B	B	B	B	B	B	B	B	B	B	B	B	B	B
E	E	E	E	E	E	E	E	E	E	E	E	E	E	E	E	E	E	E	E	E	E	E	E	E	E
B	B	B	B	B	B	B	B	B	B	B	B	B	B	B	B	B	B	B	B	B	B	B	B	B	B
E	E	E	E	E	E	E	E	E	E	E	E	E	E	E	E	E	E	E	E	E	E	E	E	E	E
A	A	A	A	A	A	A	A	A	A	A	A	A	A	A	A	A	A	A	A	A	A	A	A	A	A
E	E	E	E	E	E	E	E	E	E	E	E	E	E	E	E	E	E	E	E	E	E	E	E	E	E
A	A	A	A	A	A	A	A	A	A	A	A	A	A	A	A	A	A	A	A	A	A	A	A	A	A

165	166	167	168	169	170	171	172	173	174	175	176	177	178	179	180	181	182	183	184	185	186	187	188	189	190	191	192	193	194	195	196	197	198	199	200	201	202	203	204	205
RS12035019	RS12032662	RS649539	RS12239846	RS669697	RS17645143	RS407102	RS655315	RS943640	RS2938616	RS9429768	RS2784140	RS2603590	RS17025859	RS17629893	RS343597	RS6676798	RS1418426	RS4838884	RS694180	RS610261	RS4323713	RS2786152	RS7513936	RS1361135	RS2786967	RS2789535	RS2364815	RS2786991	RS197412	RS1373291	RS17029132	RS269110	RS155645	RS351360	RS351370	RS12144044	RS946218	RS11102603	RS773609	RS773568
A	A	A	A	A	A	A	A	A	A	A	A	A	A	A	A	A	A	A	A	A	A	A	A	A	A	A	A	A	A	A	A	A	A	A	A	A	A	A	A	A
B	B	B	B	B	B	B	B	B	B	B	B	B	B	B	B	B	B	B	B	B	B	B	B	B	B	B	B	B	B	B	B	B	B	B	B	B	B	B	B	B
C	C	C	C	C	C	C	C	C	C	C	C	C	C	C	C	C	C	C	C	C	C	C	C	C	C	C	C	C	C	C	C	C	C	C	C	C	C	C	C	C
D	D	D	D	D	D	D	D	D	D	D	D	D	D	D	D	D	D	D	D	D	D	D	D	D	D	D	D	D	D	D	D	D	D	D	D	D	D	D	D	D
E	E	E	E	E	E	E	E	E	E	E	E	E	E	E	E	E	E	E	E	E	E	E	E	E	E	E	E	E	E	E	E	E	E	E	E	E	E	E	E	E
F	F	F	F	F	F	F	F	F	F	F	F	F	F	F	F	F	F	F	F	F	F	F	F	F	F	F	F	F	F	F	F	F	F	F	F	F	F	F	F	F
G	G	G	G	G	G	G	G	G	G	G	G	G	G	G	G	G	G	G	G	G	G	G	G	G	G	G	G	G	G	G	G	G	G	G	G	G	G	G	G	G
H	H	H	H	H	H	H	H	H	H	H	H	H	H	H	H	H	H	H	H	H	H	H	H	H	H	H	H	H	H	H	H	H	H	H	H	H	H	H	H	H
I	I	I	I	I	I	I	I	I	I	I	I	I	I	I	I	I	I	I	I	I	I	I	I	I	I	I	I	I	I	I	I	I	I	I	I	I	I	I	I	I
J	J	J	J	J	J	J	J	J	J	J	J	J	J	J	J	J	J	J	J	J	J	J	J	J	J	J	J	J	J	J	J	J	J	J	J	J	J	J	J	J
E	E	E	E	E	E	E	E	E	E	E	E	E	E	E	E	E	E	E	E	E	E	E	E	E	E	E	E	E	E	E	E	E	E	E	E	E	E	E	E	E
C	C	C	C	C	C	C	C	C	C	C	C	C	C	C	C	C	C	C	C	C	C	C	C	C	C	C	C	C	C	C	C	C	C	C	C	C	D	D	D	D
F	F	F	F	F	F	F	F	F	F	F	F	F	F	F	F	F	F	F	F	F	F	F	F	F	F	F	F	F	F	F	F	F	F	F	F	F	F	F	F	F
C	C	C	C	C	C	C	C	C	C	C	C	C	C	C	C	C	C	C	C	C	C	C	C	C	C	C	C	C	C	C	C	C	C	C	C	C	C	C	C	C
E	E	E	E	E	E	E	E	E	E	E	E	E	E	E	E	E	E	E	E	E	E	E	E	E	E	E	E	E	E	E	E	E	E	E	E	E	E	E	E	E
C	C	C	C	C	C	C	C	C	C	C	C	C	C	C	C	C	C	C	C	C	C	C	C	C	C	C	C	C	C	C	C	C	C	C	C	C	C	C	C	C
E	E	E	E	E	E	E	E	E	E	E	E	E	E	E	E	E	E	E	E	E	E	E	E	E	E	E	E	E	E	E	E	E	E	E	E	E	E	E	E	E
H	H	H	H	H	H	H	H	H	H	H	H	H	H	H	H	H	H	H	H	H	H	H	H	H	H	H	H	H	H	H	H	H	H	H	H	H	H	H	H	H
E	E	E	E	E	E	E	E	E	E	E	E	E	E	E	E	E	E	E	E	E	E	E	E	E	E	E	E	E	E	E	E	E	E	E	E	E	E	E	E	E
D	D	D	D	D	D	D	D	D	D	D	D	D	D	D	D	D	D	D	D	D	D	D	D	D	D	D	D	D	D	D	D	D	D	D	D	D	D	D	D	D
E	E	E	E	E	E	E	E	E	E	E	E	E	E	E	E	E	E	E	E	E	E	E	E	E	E	E	E	E	E	E	E	E	E	E	E	E	E	E	E	E
A	A	A	A	A	A	A	A	A	A	A	A	A	A	A	A	A	A	A	A	A	A	A	A	A	A	A	A	A	A	A	A	A	A	A	A	A	A	A	A	A
E	E	E	E	E	E	E	E	E	E	E	E	E	E	E	E	E	E	E	E	E	E	E	E	E	E	E	E	E	E	E	E	E	E	E	E	E	E	E	E	E
A	A	A	A	A	A	A	A	A	A	A	A	A	A	A	A	A	A	A	A	A	A	A	A	A	A	A	A	A	A	A	A	A	A	A	A	A	A	A	A	A
F	F	F	F	F	F	F	F	F	F	F	F	F	F	F	F	F	F	F	F	F	F	F	F	F	F	F	F	F	F	F	F	F	F	F	F	F	F	F	F	F
I	I	I	I	I	I	I	I	I	I	I	I	I	I	I	I	I	I	I	I	I	I	I	I	I	I	I	I	I	I	I	I	I	I	I	I	I	I	I	I	I
E	E	E	E	E	E	E	E	E	E	E	E	E	E	E	E	E	E	E	E	E	E	E	E	E	E	E	E	E	E	E	E	E	E	E	E	E	E	E	E	E
G	G	G	G	G	G	G	G	G	G	G	G	G	G	G	G	G	G	G	G	G	G	G	G	G	G	G	G	G	G	G	G	G	G	G	G	G	G	G	G	G
C	C	C	C	C	C	C	C	C	C	C	C	C	C	C	C	C	C	C	C	C	C	C	C	C	C	C	C	C	C	C	C	C	C	C	C	C	C	C	C	C
G	G	G	G	G	G	G	G	G	G	G	G	G	G	G	G	G	G	G	G	G	G	G	G	G	G	G	G	G	G	G	G	G	G	G	G	G	G	G	G	G
E	E	E	E	E	E	E	E	E	E	E	E	E	E	E	E	E	E	E	E	E	E	E	E	E	E	E	E	E	E	E	E	E	E	E	E	E	E	E	E	D
B	B	B	B	B	B	B	B	B	B	B	B	B	B	B	B	B	B	B	B	B	B	B	B	B	B	B	B	B	B	B	B	B	B	B	B	B	B	B	B	B
E	E	E	E	E	E	E	E	E	E	E	E	E	E	E	E	E	E	E	E	E	E	E	E	E	E	E	E	E	E	E	E	E	E	E	E	E	E	E	E	E
B	B	B	B	B	B	B	B	B	B	B	B	B	B	B	B	B	B	B	B	B	B	B	B	B	B	B	B	B	B	B	B	B	B	B	B	B	B	B	B	B
E	E	E	E	E	E	E	E	E	E	E	E	E	E	E	E	E	E	E	E	E	E	E	E	E	E	E	E	E	E	E	E	E	E	E	E	E	E	E	E	E
B	B	B	B	B	B	B	B	B	B	B	B	B	B	B	B	B	B	B	B	B	B	B	B	B	B	B	B	B	B	B	B	B	B	B	B	B	B	B	B	B
E	E	E	E	E	E	E	E	E	E	E	E	E	E	E	E	E	E	E	E	E	E	E	E	E	E	E	E	E	E	E	E	E	E	E	E	E	E	E	E	E
A	A	A	A	A	A	A	A	A	A	A	A	A	A	A	A	A	A	A	A	A	A	A	A	A	A	A	A	A	A	A	A	A	A	A	A	A	A	A	A	A
E	E	E	E	E	E	E	E	E	E	E	E	E	E	E	E	E	E	E	E	E	E	E	E	E	E	E	E	E	E	E	E	E	E	E	E	E	E	E	E	E
A	A	A	A	A	A	A	A	A	A	A	A	A	A	A	A	A	A	A	A	A	A	A	A	A	A	A	A	A	A	A	A	A	A	A	A	A	A	A	A	A
